# The microstructures of promoted RANEY® nickel alloys and catalysts

**DOI:** 10.1039/d6ra05868j

**Published:** 2026-11-04

**Authors:** Thomas A. Manz, Seqwana T. Pryor, Kevin P. Trumble, Zhufang Liu, David R. Gaskell, Dave Roberts, Carle Hager, Sourav K. Sengupta, Theodore A. Koch, W. Nicholas Delgass

**Affiliations:** a School of Chemical Engineering, Purdue University West Lafayette IN 47907 USA tmanz@nmsu.edu; b DuPont Wilmington DE 19803 USA; c School of Materials Engineering, Purdue University West Lafayette IN 47907 USA; d Electron Microscopy Center, Purdue University West Lafayette IN 47907 USA

## Abstract

RANEY® nickel catalysts are widely used in both academia and industry to hydrogenate nitro compounds, carbonyls, alkenes, alkynes, nitriles, and vegetable oils. However, there has been a gap in knowledge concerning the microstructures of these materials, particularly when they are promoted with Fe and/or Cr. In this work, we studied the following RANEY® nickel alloys and catalysts: (i) unpromoted, (ii) multiple levels of Fe promotion, (iii) multiple levels of Cr promotion, and (iv) doubly-promoted with Fe and Cr. Microstructures and phase compositions of initial alloys were characterized using backscattered scanning electron microscopy (SEM), electron microprobe, optical microscopy, and X-ray diffraction, which showed the microstructure is composed of several phases: Al_3_Ni_2_, Al_3_Ni, promoter-rich phase(s) (which are absent in the unpromoted alloy), and a eutectic phase. In these alloys, the Fe and Cr promoters concentrated into promoter-rich phases, and we report the compositions of these phases. After crushing the alloys into powder and leaching in aqueous sodium hydroxide, the catalysts were washed and characterized. Nitrogen adsorption at 77 K yielded each catalyst's total surface area and pore size distribution. Carbon monoxide and hydrogen gas adsorption yielded each catalyst's metallic surface area. For some catalysts, bulk elemental composition was quantified using atomic absorption spectroscopy, and the surface compositions and oxidation states were studied using X-ray photoelectron spectroscopy (XPS). XPS results showed that Cr is oxidized to Cr(+III) during the leaching procedure to form a compound that increases the total surface area (while having negligible effect on the metallic surface area) and also leads to smaller pore diameters.

## Abstract 2

This article uses many characterization techniques to achieve new insights into effects of Fe and Cr promotion on microstructures of RANEY® nickel alloys and catalysts, which have widespread importance as hydrogenation catalysts.

## Graphical abstract

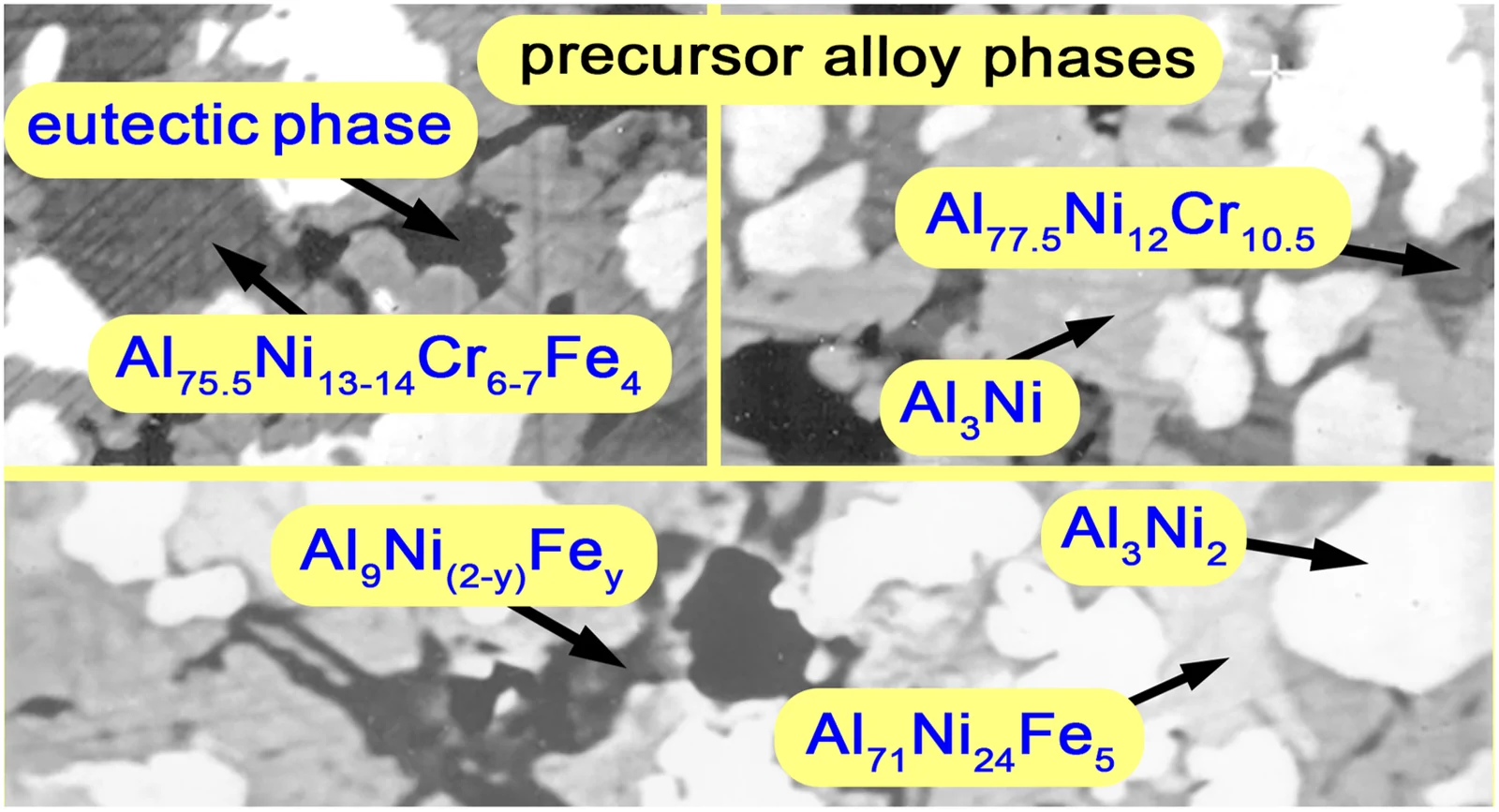

## 1. Introduction

### 1.1 The Al–Ni phase diagram and unpromoted RANEY® alloys

RANEY® nickel catalysts are formed by leaching an alloy containing metallic nickel in aqueous acid or base solution to oxidatively remove a reactive metal leaving behind a microporous sponge nickel material.^1–6^ For example, Ni–Al alloys can be leached in concentrated sodium hydroxide aqueous solutions to oxidatively dissolve the Al *via* the following leaching reaction:^10,32,53,58,121–123^1
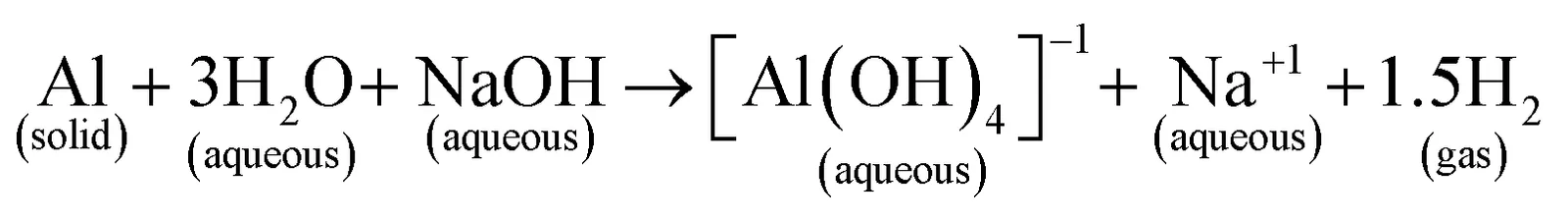


Unpromoted RANEY® Ni–Al alloys are primary composed of three phases: NiAl_3_, eutectic, and Ni_2_Al_3_.^7–10^ All three of these phases can be leached. However, the more nickel-rich NiAl phase cannot be leached as shown by the following experiment. We ordered a crushed powder of NiAl from a chemical supplier and placed it in 30 wt% sodium hydroxide aqueous solution. No hydrogen evolution occurred. From this experiment, we concluded that NiAl should generally be avoided in the RANEY® Ni–Al alloys, because it is unleachable ‘dead weight’.

The composition and cooling profile determine the phase structure and therefore the properties of the alloy. Two commercial variations of the composition are 42 and 50 wt% nickel, with the remainder aluminum. As indicated by the Al–Ni phase diagram in Fig. 1 (reproduced from ref. 11), at equilibrium for temperatures below the eutectic temperature (640 °C), both of these alloy compositions will contain a mixture of NiAl_3_, eutectic, and Ni_2_Al_3_ phases. It is also possible for the 50 wt% nickel alloy to contain some residual meta-stable (*i.e.*, non-equilibrium) NiAl phase; however, the peritectic reaction in which NiAl reacts with the melt to produce Ni_2_Al_3_ often effectively goes to completion so that no appreciable amounts of NiAl phase remain.^7,12^ Peritectic refers to an isothermal reversible reaction in which a liquid phase reacts with a solid phase during cooling to produce a second solid phase.

**Fig. 1:**
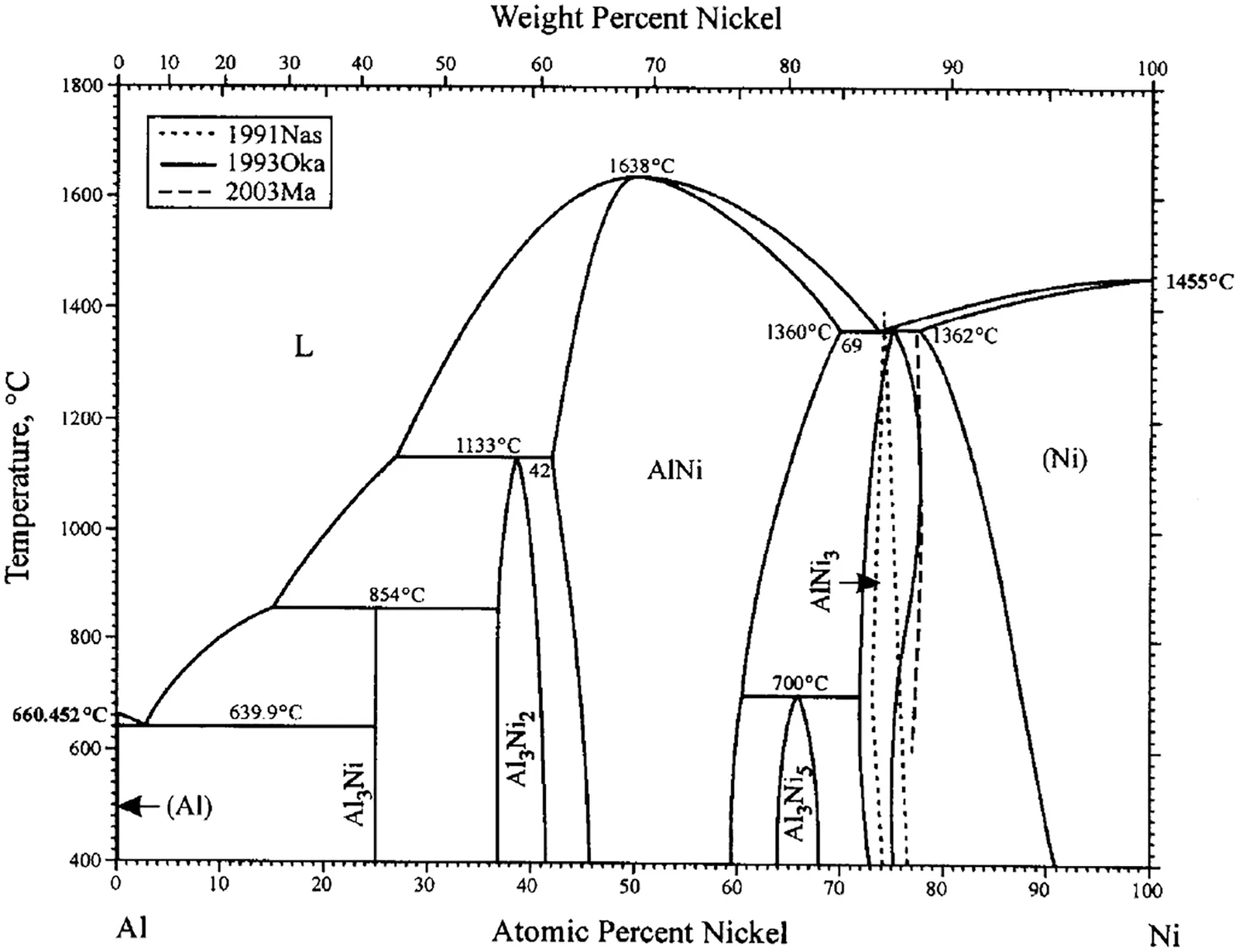
The Al–Ni phase diagram. Reproduced with permission from H. Okamoto, *J. Phase Equilib. Diffus.*, 2004, **25**, 394. Copyright 2004 ASM International.

During heating, when the temperature reaches 660 °C, the aluminum melts and begins to dissolve the solid nickel. For the 42 wt% Ni alloy, all of the nickel will be dissolved when the temperature reaches 1100 °C. For the 50 wt% Ni alloy, the temperature must reach 1340 °C before all of the nickel dissolves. Generally, the temperature is held above these melting temperatures for some time to allow for complete dissolution of the nickel.

As the liquid is cooled, the first phase to form should be NiAl for the 50 wt% alloy. Below a temperature of 1133 °C, the NiAl phase undergoes a peritectic reaction with the liquid phase to form the Ni_2_Al_3_ phase. In the 42 wt% Ni alloy, NiAl is not formed, and Ni_2_Al_3_ is the first phase to form. It forms just below the melting temperature. Further cooling of either of these alloys produces NiAl_3_ by a peritectic reaction between the liquid and solid N_2_Al_3_ at 854 °C. This reaction is slow, because solid NiAl_3_ forms between the two phases and prevents further contact.

Below the peritectic temperature of 854 °C, solid NiAl_3_ precipitates from the melt and the melt becomes enriched in aluminum. Finally, at the eutectic point (640 °C), the eutectic solidifies all at once without further change in composition. (Eutectic refers to an alloy composition that has the lowest possible constant melting point.) The eutectic is approximately 6 wt% Ni and comprises thin rods or lamellae (plates) of NiAl_3_ dispersed within an Al matrix.

The grain size can be controlled by the cooling rate. Arc melting, melt spinning, and quenching are three techniques which can be used to rapidly cool the alloy and produce small grains.^63,99^ Large grains can be produced by a slow cooling rate.

Once formed, the solid alloy may be reheated (*i.e.*, annealed) just below the peritectic temperature of 854 °C. This causes the sluggish peritectic reaction to proceed without substantial change in the overall grain size. The peritectic reaction increases the amount of NiAl_3_ at the expense of the other two phases.

In summary, the following three factors can be used to control grain size and phase distribution in the alloy: (1) composition, (2) cooling rate, and (3) annealing.^63^ Hu *et al.* investigated the effects of rapid quenching of the 50 wt% Ni, 50 wt% Al alloy on the microstructure of the resultant leached catalyst, and some differences were found compared to the material that was not rapidly quenched.^8^

### 1.2 Prior studies of alloy and catalyst microstructures

Here, we briefly mention some prior studies on RANEY® nickel alloy and catalyst microstructures. Several article series explored various alloy compositions and applied a wide variety of techniques to characterize the alloys or leached catalysts: magnetic measurements, thermal programmed desorption (TPD), X-ray diffraction (XRD), electron microscopy and electron microprobe, gas adsorption, impedance measurements, X-ray photoelectron spectroscopy (XPS), neutron inelastic spectroscopy, *etc.*

Anderson and co-workers published an extensive multi-part series of journal articles on the structure of RANEY® nickel.^9,13–19^ Using microprobe analysis, they followed the progress of catalyst leaching and determined the NiAl_3_ phases leaches more quickly than the Ni_2_Al_3_ phase.^9,13^ They also studied magnetic properties of the activated catalyts.^14,15^ They also measured CO, H_2_, and liquid N_2_ adsorption properties of the catalysts.^18,19^

Petro and co-workers published an extensive multipart series on the complex structure of nickel skeleton catalysts derived from various alloy types (*e.g.*, Ni–Al, Ni–Si, Ni–Mg, Ni–Zn), some of which produced non-pyrophoric catalysts.^20–30^ These were patented,^142^ and their magnetic, thermodesorption, catalytic, and stability properties were extensively studied.

Fasman and co-workers published several journal articles investigating diverse alloy compositions (including a wide variety of different promoter elements), leaching conditions, and storage conditions.^12,31–35^ They studied the formation of various surface oxides during leaching that remained on the catalyst's surface. Ref. 35 reviews these studies that spanned more than 40 years.

Fouilloux and co-workers published many articles investigating the pore structure, adsorbed hydrogen, structure of adsorbed water, and catalytic activity of promoted and unpromoted RANEY® nickel catalysts.^36–47^ Using neutron scattering, they studied the diffusion of hydrogen atoms on the catalyst's surface.^43,46^ They studied the catalyst's pore structure and texture using impedance measurements and other techniques.^38,39,47^ They also found that the catalyst is poisoned by thiourea.^45^

The systematic studies cited in the prior paragraphs were published during the 1960's to 1990's time period. Even before that time period, some of the hydrogen produced during leaching that remained bound to nickel atoms in the activated catalyst had attracted the attention of researchers.^48–50^ Also, earlier studies by Adkins and co-workers had defined various members of the W-series of RANEY® nickel hydrogenation catalysts based on specific leaching conditions.^51,52^

More recently, several studies published since 2000 provided important new details regarding the microstructures of RANEY® nickel alloys and catalysts. Rodella *et al.* conducted a textural and structural analyses of an industrial RANEY® nickel alloy and resulting catalysts characterized by XRD, scanning and transmission electron microscopies, XPS, cryogenic nitrogen adsorption, and small-angle X-ray scattering (SAXS).^53^ Sun *et al.* reviewed applications of RANEY® nickel for biomass conversion including a discussion of catalyst structure.^54^ Recent advances in computing power enabled large-scale computational modeling studies into the catalyst structure and leaching process.^55–58^

### 1.3 Catalyst promotion

There is a long history of adding various chemical elements or molecules in small quantities as ‘promoters’ to RANEY® nickel alloys and catalysts to improve their catalytic performance.^4,37,59–62^ Montgomery published a study on the effects of various transition metal promoters in RANEY® nickel catalysts for reducing various functional groups in molecules.^60^ Catalysts promoted with Cr and Mo were found to have high activities for hydrogenating organic compounds.^60^

In this study, we focus on promotion with Fe and/or Cr. Several prior studies investigated RANEY® nickel alloys and/or catalysts promoted with Fe and/or Cr.^32,33,35,63–65,137–140^ A 1975 patent by Chabert describes a RANEY® nickel catalyst doubly promoted with Fe and Cr for the hydrogenation of nitriles to amines.^66^

Kordulis *et al.* studied the microstructures of Cr-doped RANEY® nickel precursor alloys and activated catalysts.^136^ Using XPS, they observed that Cr was completely oxidized and accumulated on the catalyst's surface.^136^

Hoffer *et al.* conducted an extensive study of a Fe, Cr doubly-promoted RANEY® nickel alloy and catalyst using XRD, cryogenic N_2_ adsorption, H_2_ adsorption and thermal programmed desorption, XPS, high-resolution transmission electron microscopy (HRTEM), bulk and surface elemental analyses, and hydrogenation reactions.^67^ They observed that the Fe, Cr doubly-promoted catalyst had a higher total surface area than the unpromoted catalyst, and the Fe, Cr doubly-promoted catalyst also retained a higher percentage of its reactivity after several recycles than the unpromoted catalyst.^67^

Several studies also investigated iron promotion in mechanically-produced (*via* ball milling) sponge nickel alloys.^68–70,141^ Active catalysts were produced by leaching those mechanically-alloyed materials.^68–70,141^

Bonnier *et al.* studied catalysts produced by leaching a Al_3_Ni_(2−*x*)_Cr_*x*_ precursor alloy and found a correlation between its catalytic properties and the amount of Cr(+III) on the catalyst's surface.^36,71^ Moroz *et al.* found that a RANEY® nickel catalyst derived from a Fe-promoted alloy contained a Ni–Fe solid solution, while a RANEY® nickel catalyst derived from a Cr-promoted alloy contained highly dispersed surface Cr_2_O_3_ moiety.^72^ On the other hand, Hoffer *et al.*’s XPS results showed both Cr(+III) and Fe(+III) oxidized forms on the surface of a Fe, Cr doubly-promoted RANEY® nickel catalyst.^67^

Considering the role of surface chromium oxide or chromium hydroxide in the chromium-promoted catalysts, Masson and co-workers developed a post-treatment in which an initially unpromoted RANEY® nickel catalyst is soaked in an aqueous solution containing Cr(+III) ions to deposit chromium hydroxide on the catalyst's surface.^36^ They compared the microstructure and catalytic performance of this chromium-promoted catalyst to one prepared by leaching the Al–Ni–Cr alloy.^36,73–75^ They found that Cr promotion increases the selectivity for hydrogenating the double bond of acetophenone compared to side reactions that hydrogenate its aromatic ring.^75^

Our present study more fully characterizes how Fe promotion, Cr promotion and Fe, Cr double promotion impact the microstructures of the RANEY® nickel alloys and catalysts. Most importantly, we identify several of the promoter-rich phases in these alloys. This provides a new understanding of these materials.

## 2. Alloy synthesis and characterization

### 2.1 Unpromoted Ni–Al alloy made by arc melting

A 50 wt% nickel alloy was prepared by arc melting 6.4755 g Ni and 6.4659 g Al. In this technique, an electric arc was used to heat aluminum to the melting point under a glass dome filled with an inert gas. The base of the dome was a water-cooled copper plate. Once the aluminum was melted, pieces of nickel were slowly added to the melt. Mixing was good, because the electric current stirred the melt. Once all of the nickel pieces were added, the current was turned off. The sample solidified rapidly as heat diffused to the water-cooled hearth.

A sample was prepared for optical microscopy using the following procedure. First, an alloy ingot was cut with a diamond saw to produce a flat surface. During this process, the blade was lubricated by dripping oil onto it. Next, the flat surface of the sample was sanded on 240, 320, 400, and 600 wet grit papers. The sample was rinsed in water and rotated 90° between each step. Once all scratches were removed perpendicular to the sanding direction, the sample was rotated and moved to the next grit. After sanding, the sample was polished on an 800 grit wheel, a diamond wheel (4–8 micron), and finally an alumina wheel (0.05 micron). The sample was gently rotated during polishing. Sample preparation was complete when the surface appeared mirror-like and free of visible scratches. Once the sample was polished, photos of its microstructure were taken using a reflective optical microscope equipped with a Polaroid camera.

The remaining portion of the billet was mounted in a 1 inch bakelite press. The sample was placed face-down in the press and covered with about 1.5 cm of bakelite powder. The press was turned on and heated to cure the bakelite. After curing, the sample was removed and polished in a manner similar to the above procedure. Backscattered scanning electron microscopy (SEM) and electron microprobe studies were done on the bakelite mounted sample.

Fig. 2 shows the phase structure of the 50 wt% Ni alloy prepared by arc melting. This sample had a small grain size, because it was cooled quickly. Electron microprobe analysis showed that the three phases present were Ni_2_Al_3_, NiAl_3_, and eutectic. As shown in Fig. 2, the first phase to form was Ni_2_Al_3_. As the melt was cooled further, the Ni_2_Al_3_ phase reacted with the liquid phase by a peritectic reaction to form NiAl_3_. Also, some NiAl_3_ formed *via* direct solidification from the melt. NiAl_3_ is the phase surrounding Ni_2_Al_3_ in the optical micrograph shown in Fig. 2. The eutectic phase formed last and filled the space between the larger grains of NiAl_3_.

**Fig. 2:**
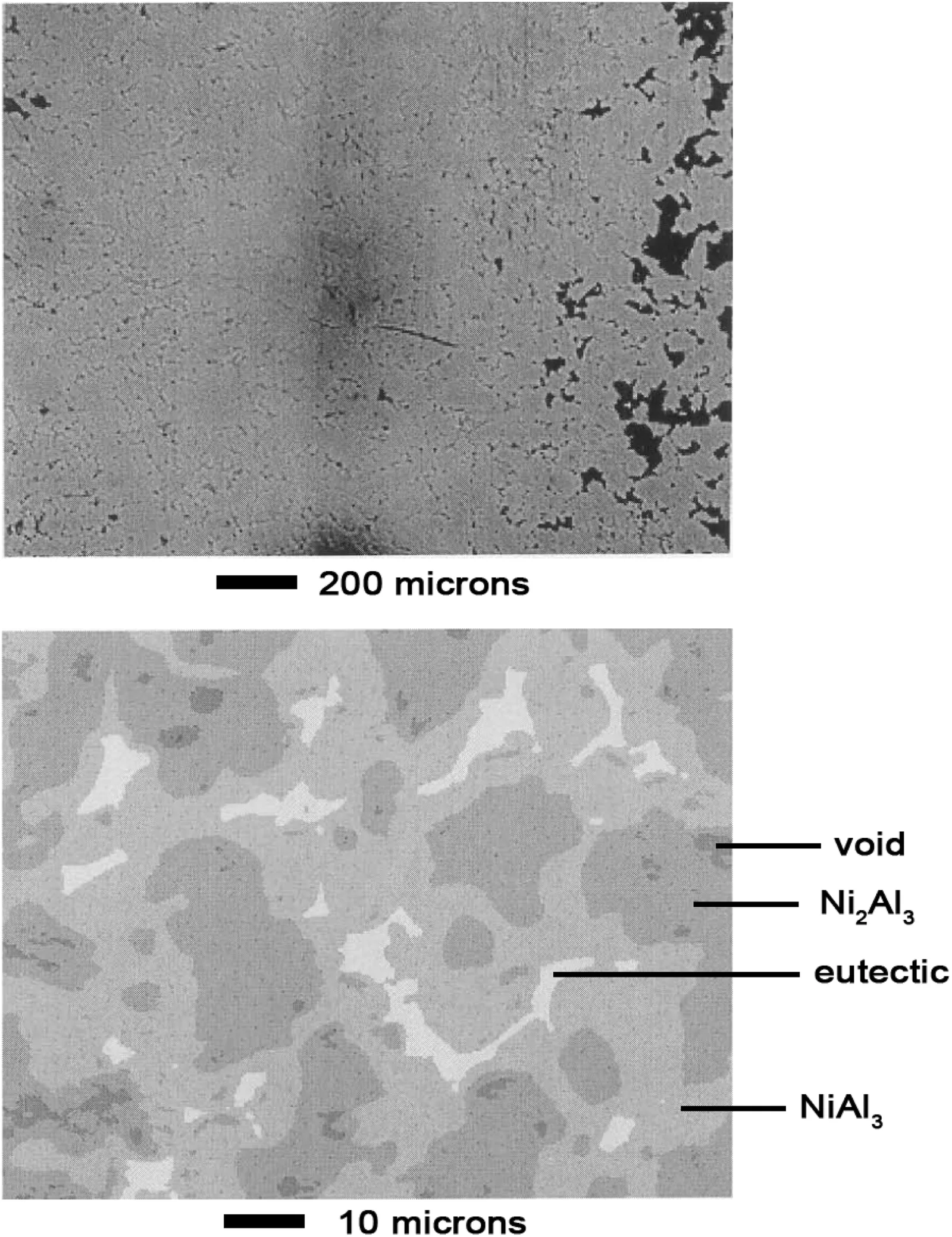
Optical micrographs of the arc melted Ni–Al alloy before leaching. The bottom panel was taken at higher magnification than the top panel. The Ni_2_Al_3_, NiAl_3_, and eutectic phases are labeled. A void is also labeled.

A 3 wt% NaOH aqueous solution was used to etch the surface, making the phase structure visible. As the surface was leached, it became darker. The aluminum-rich eutectic phase was the first to be attacked by alkali. After just 1 minute, the eutectic phase was dissolved, leaving an empty space between the grains as shown in Fig. 3.

**Fig. 3:**
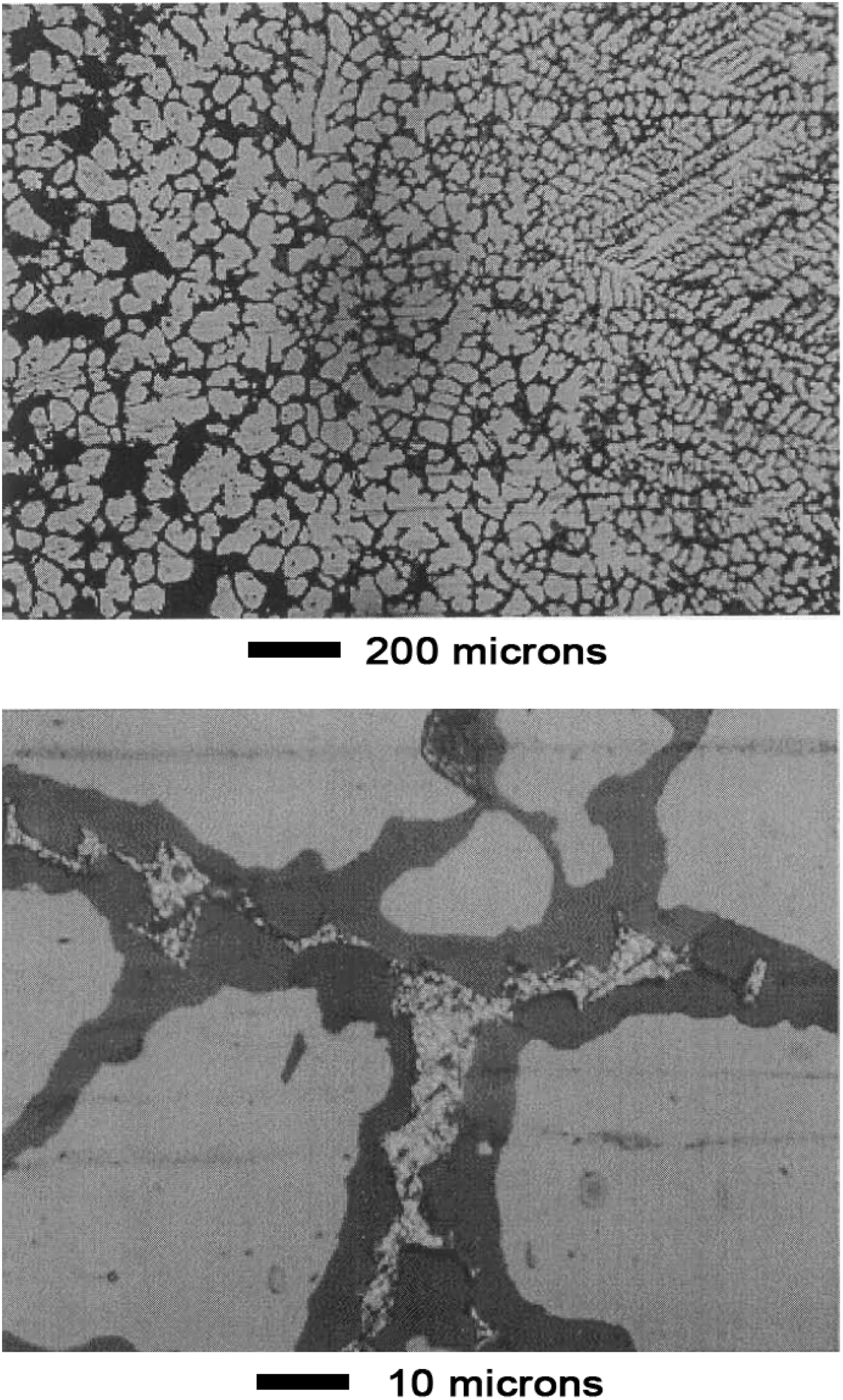
Optical micrographs of the arc melted Ni–Al alloy after leaching 1 minute in 3 wt% NaOH aqueous solution. The bottom panel was taken at higher magnification than the top panel.

The NiAl_3_ phase was leached easily and appeared dark after just 1 minute. After 16 minutes, it developed a broken-up ‘barnacle-like’ structure. Small dark particles were formed from the NiAl_3_ phase and clumped outside of the Ni_2_Al_3_ phase as shown in Fig. 4. As shown in Fig. 4, the Ni_2_Al_3_ phase was the most difficult phase to leach.

**Fig. 4:**
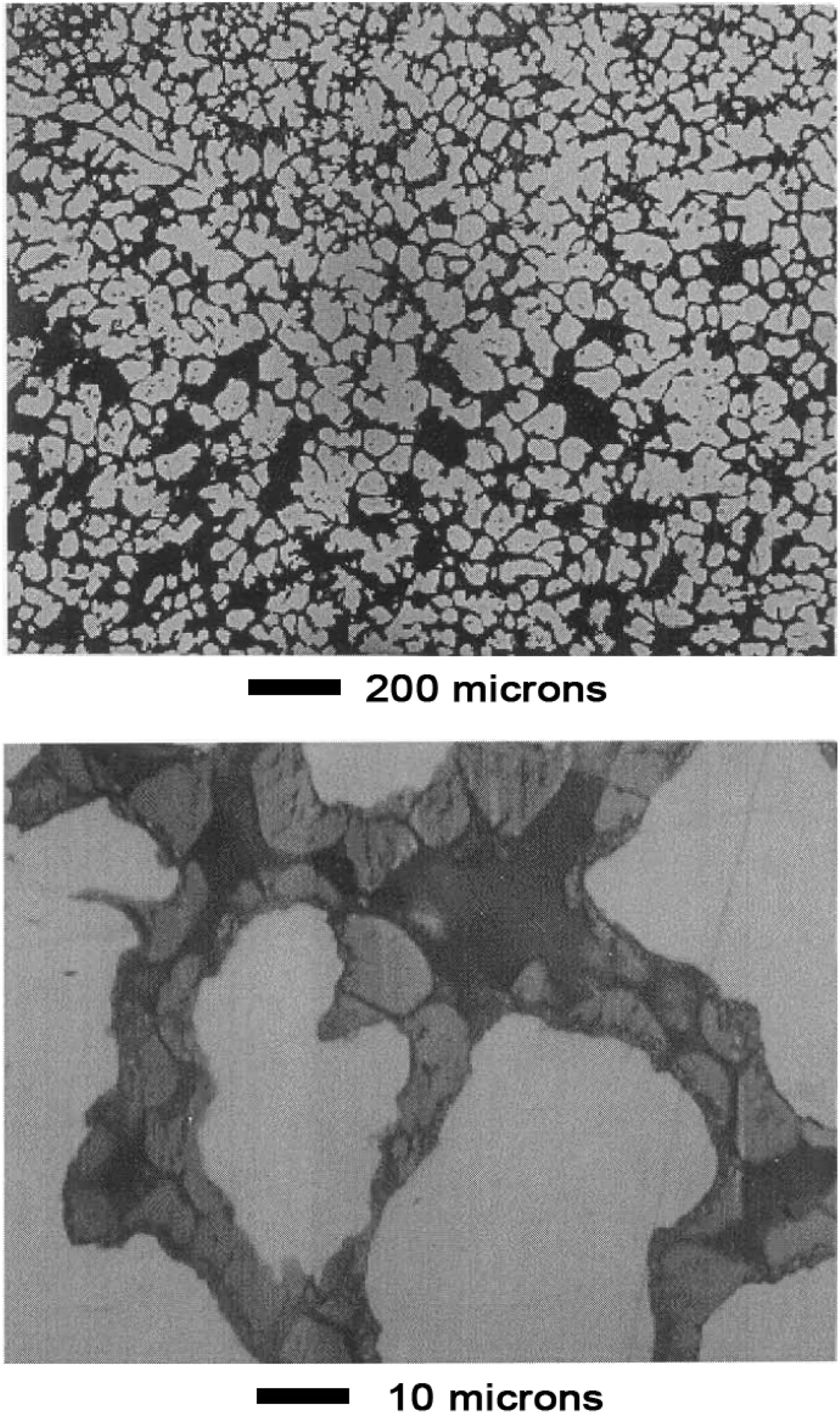
Optical micrographs of the arc melted Ni–Al alloy after leaching 16 minutes in 3 wt% NaOH aqueous solution. The bottom panel was taken at higher magnification than the top panel.

### 2.2 Unpromoted and promoted Ni–Al alloys made by induction furnace

Precursor alloys were prepared in an induction furnace in the School of Materials Engineering at Purdue University. The starting alloys contained 42 wt% nickel. The synthesis began by accurately weighing the amounts of each element (99.99% pure). Unpromoted and chromium, iron, and chromium–iron promoted nickel–aluminum alloys were prepared as shown in Table 1.

**Table 1:** Summary of precursor alloy charges. The compositions are given in weight percent

Sample heat	Composition	Charge (grams)			
		Ni	Al	Fe	Cr
#1 (2% Cr)	42/56/2	126.24	168.08	0	6.25
#2 (1% Cr)	42/57/1	126.37	171.32	0	3.50
#3 (1.5% Fe, Cr)	42/55/1.5/1.5	126.31	165.06	4.70	4.77
#4 (4% Cr)	42/54/4	252.8	325	0	24.1
#5 (unpromoted)	42/58	175.79	242	0	0
#6 (1% Fe)	42/57/1	125.45	171.13	3.21	0
#7 (4% Fe)	42/54/4	126.98	162.29	12.59	0
#8 (2% Fe)	42/56/2	127.05	168.08	6.83	0

Before alloy synthesis, the aluminum was cleaned in a 20 wt% NaOH aqueous solution to remove the surface oxide. The nickel was sand blasted and washed in *aqua regia* solution to remove hard surface contaminants. The charge was slowly heated in a graphite crucible in the induction furnace under flowing inert (*e.g.*, argon) gas to prevent oxidation. The charge was held for approximately 2 hours above the alloy's melting point to allow sufficient time for the nickel (and Fe and Cr promoters when present) to dissolve into the molten aluminum. The melt was then cooled in the furnace to solidify. Finally, the hot solid ingot was quenched in water to room temperature.

The crystalline alloys were characterized using X-ray diffraction (XRD). XRD patterns of the alloys were measured by a Siemens D500 diffractometer using a Cu tube X-ray source (*λ* = 1.5406 Å). Powdered samples were dusted on double-sided sticky tape and mounted on a glass microscope slide and placed in the diffractometer. The patterns were recorded over a range of 2*θ* angles from 5° to 105° and compared to reference X-ray powder files. SI Section S1 contains the XRD spectra of these eight alloys. Peaks corresponding to Ni_2_Al_3_, NiAl_3_, and Al were labeled. However, this labeling does not have a one-to-one correspondence with the phases shown in the optical and SEM micrographs. Specifically, the eutectic phase is a mixture of Al (high percentage) and NiAl_3_ (small percentage). Also, some peaks for the promoter-rich phases (discussed in Section 3 below) either overlapped with peaks from the Ni_2_Al_3_, NiAl_3_, or Al phases or else were not labeled in these XRD spectra.

Samples were prepared for optical microscopy and electron microprobe analysis using the same procedure described in the previous section. SI Section S2 contains optical micrographs for these eight Purdue alloys.

Backscattered SEM micrographs and electron microprobe results are presented in Fig. 5 (unpromoted alloy), Fig. 6 (1 wt% Cr alloy), Fig. 7 (2 wt% Cr alloy), Fig. 8 (4 wt% Cr alloy), Fig. 9 (1 wt% Fe alloy), Fig. 10 (2 wt% Fe alloy), Fig. 11 (4 wt% Fe alloy), and Fig. 12 (Fe–Cr doubly-promoted alloy). Electron microprobe was performed on multiple spots within each phase, and the average composition ± standard deviation within the same phase is listed. A 15.0 kV accelerating voltage was used for the electron microprobe analysis.

**Fig. 5:**
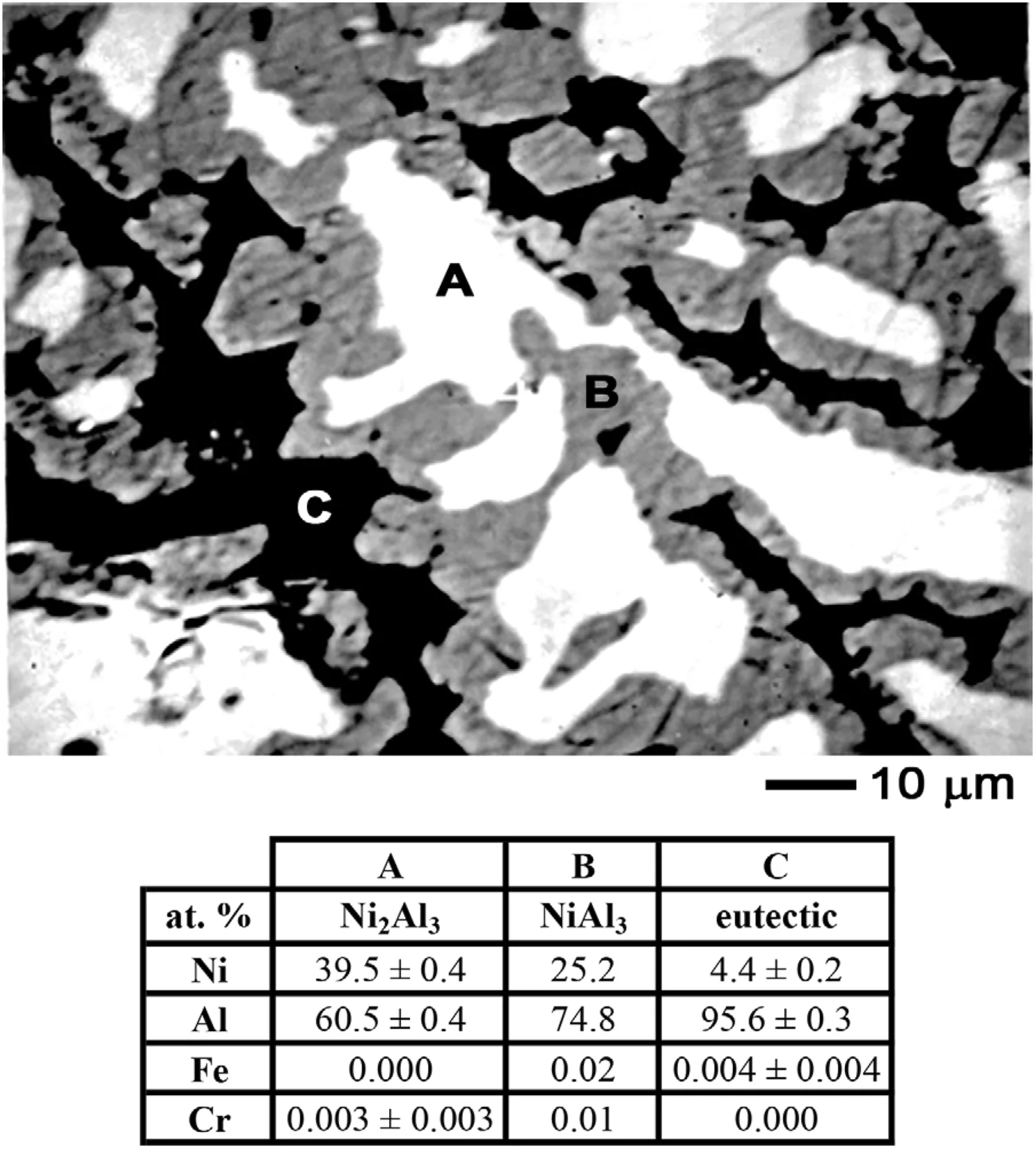
Backscattered SEM micrograph and electron microprobe results for the unpromoted Ni–Al RANEY® alloy. The phase compositions are given in atomic percentage. Standard deviations are not available for the NiAl_3_ phase, because only one location was analyzed for this phase.

**Fig. 6:**
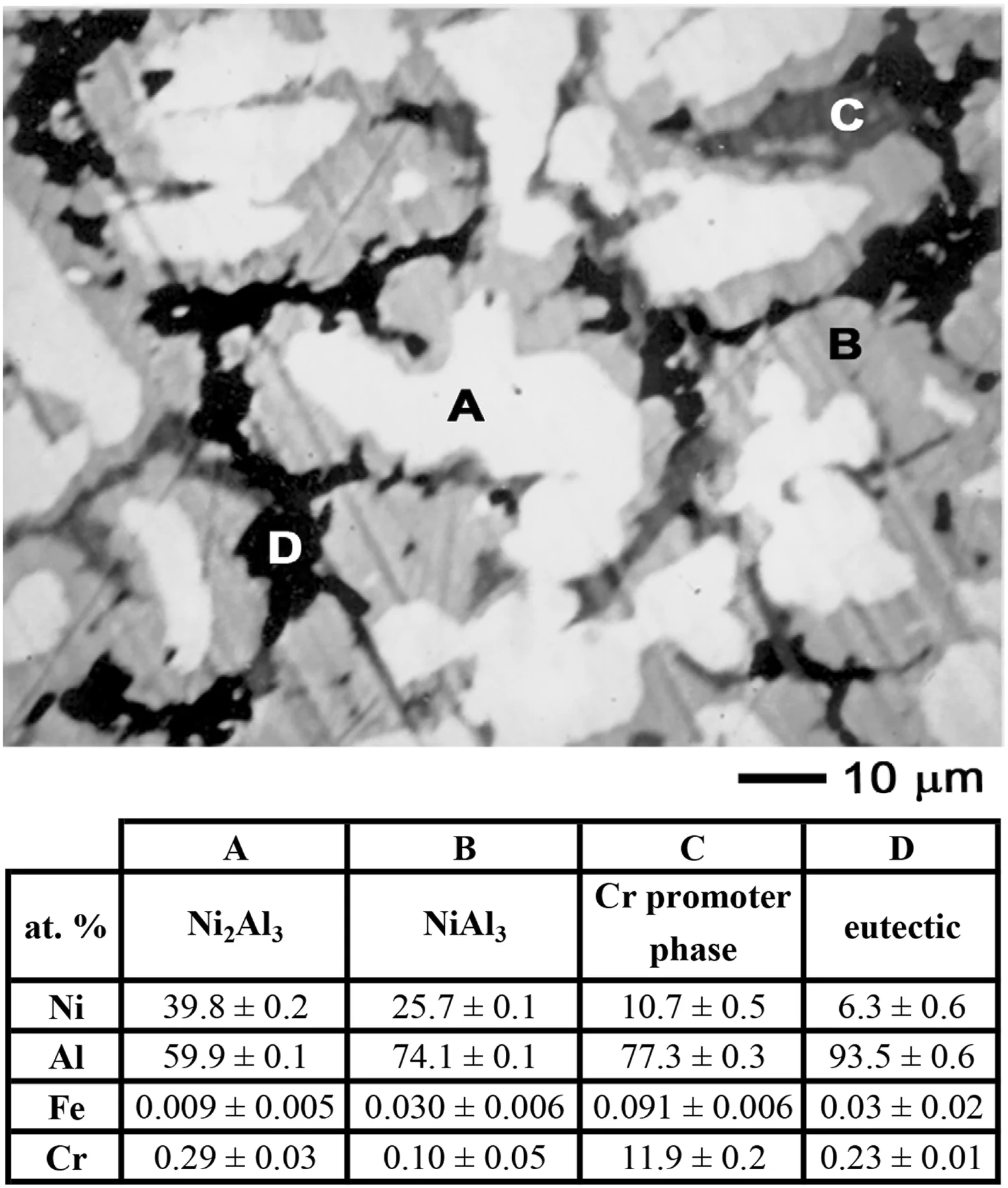
Backscattered SEM micrograph and electron microprobe results for the 1 wt% Cr promoted Ni–Al RANEY® alloy. The phase compositions are given in atomic percentage.

**Fig. 7:**
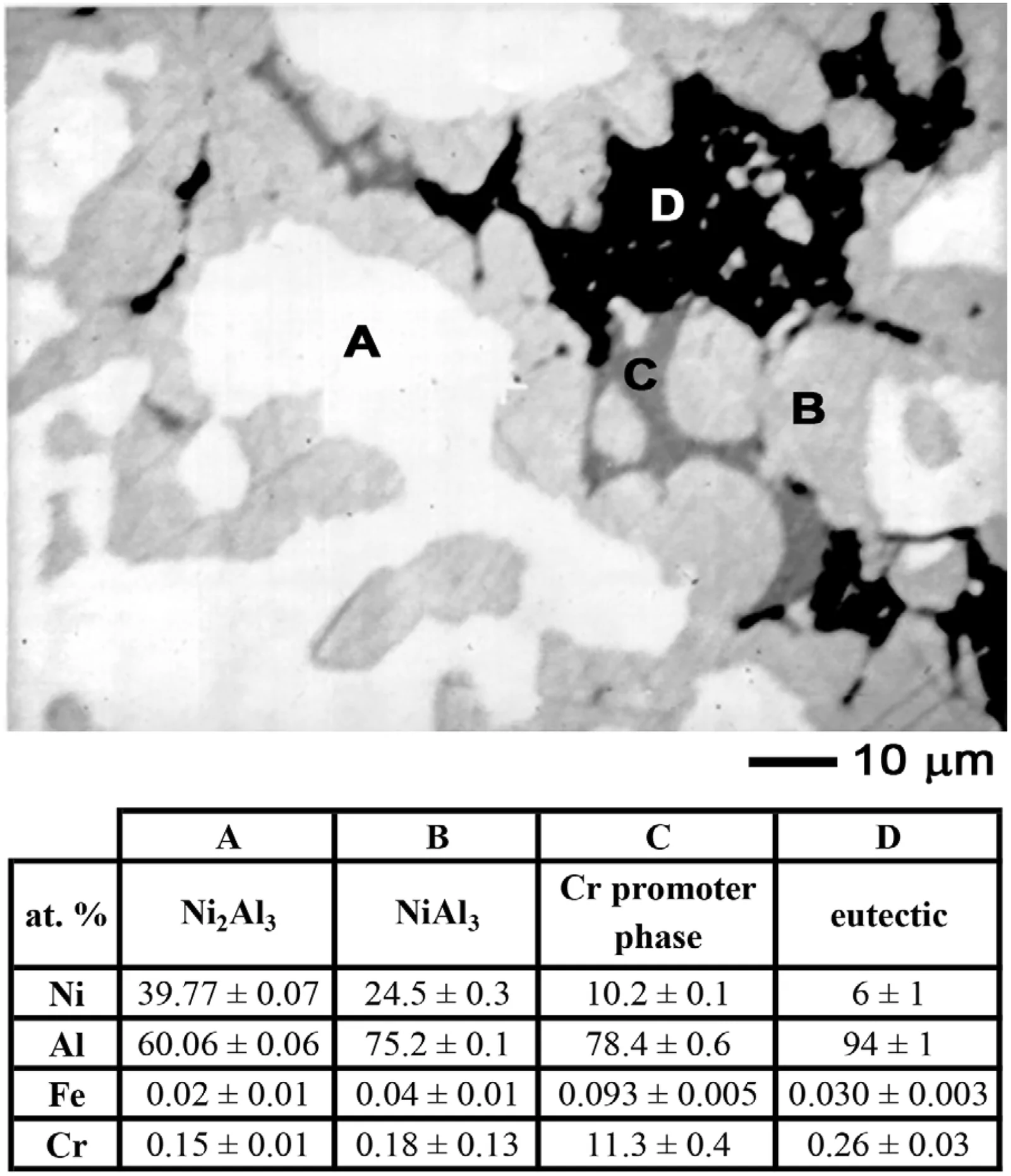
Backscattered SEM micrograph and electron microprobe results for the 2 wt% Cr promoted Ni–Al RANEY® alloy. The phase compositions are given in atomic percentage.

**Fig. 8:**
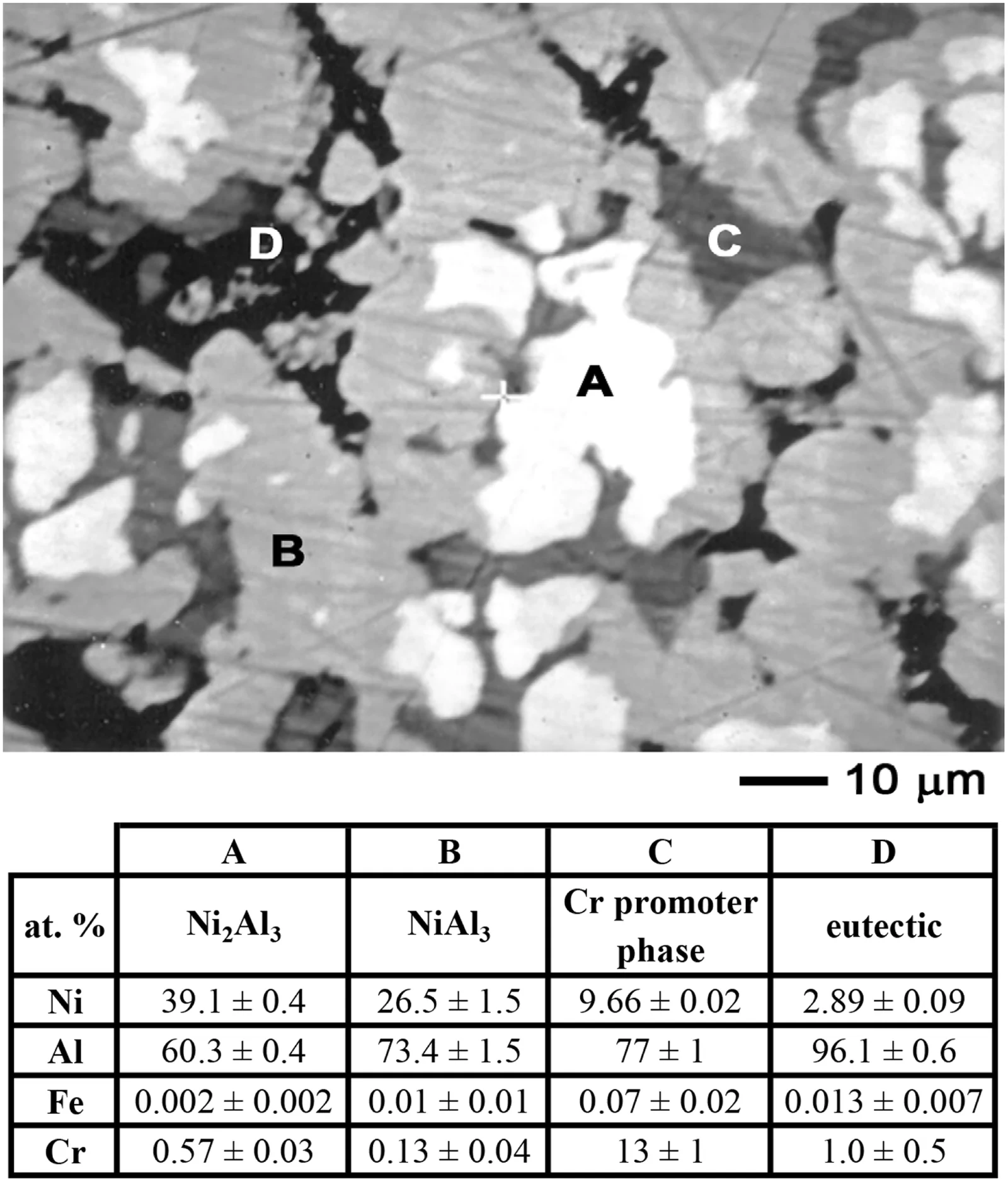
Backscattered SEM micrograph and electron microprobe results for the 4 wt% Cr promoted Ni–Al RANEY® alloy. The phase compositions are given in atomic percentage.

**Fig. 9:**
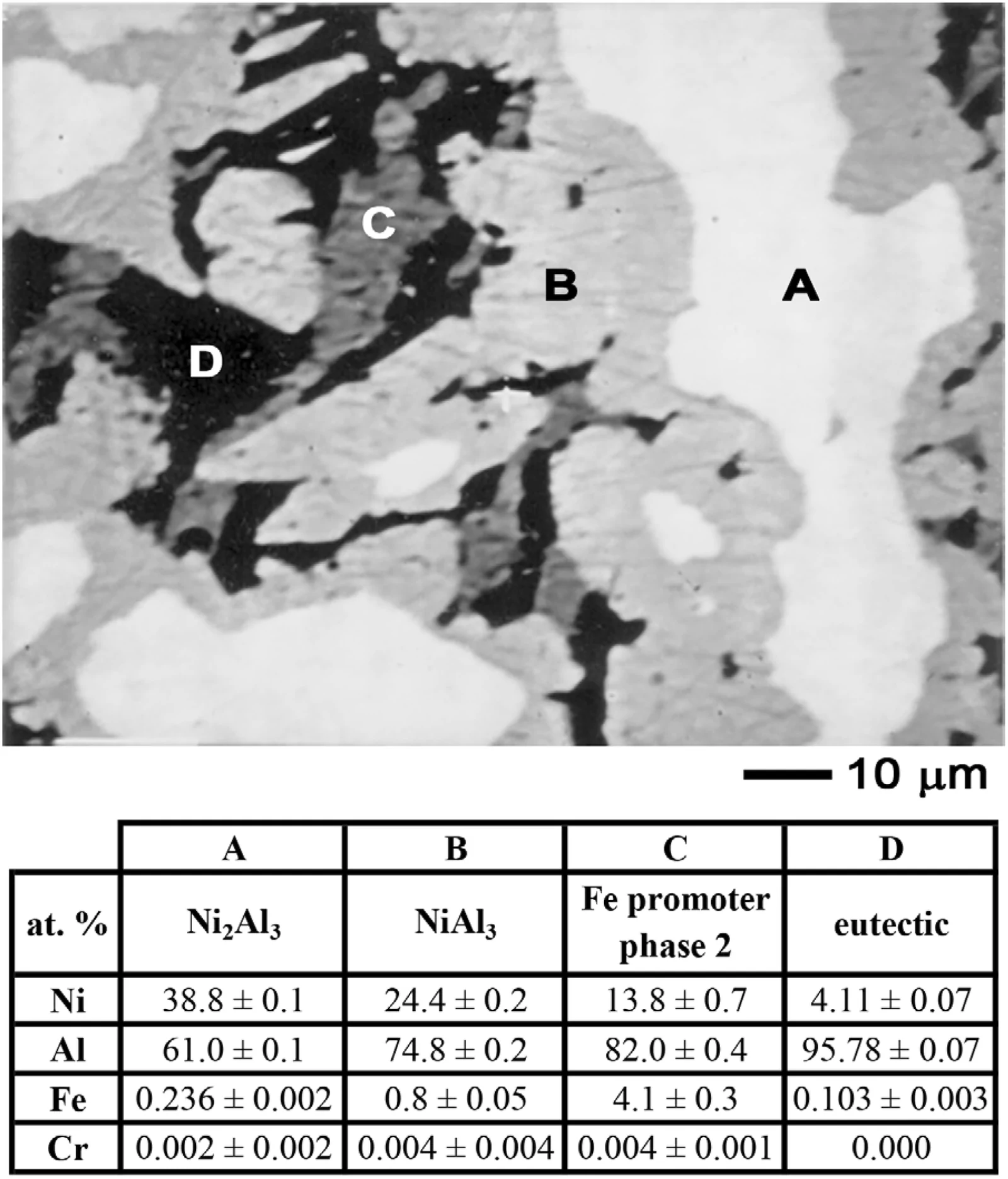
Backscattered SEM micrograph and electron microprobe results for the 1 wt% Fe promoted Ni–Al RANEY® alloy. The phase compositions are given in atomic percentage.

**Fig. 10:**
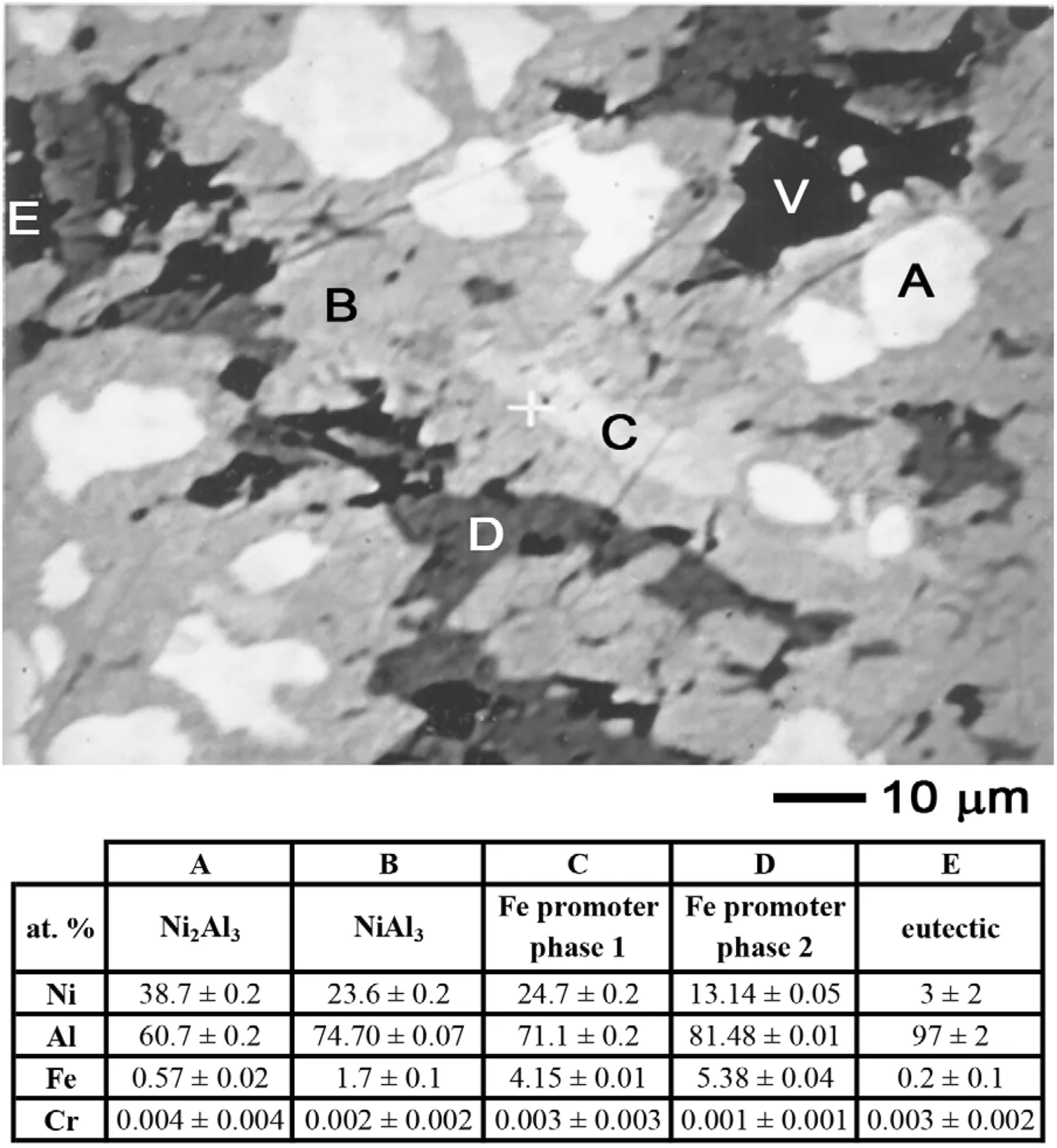
Backscattered SEM micrograph and electron microprobe results for the 2 wt% Fe promoted Ni–Al RANEY® alloy. The phase compositions are given in atomic percentage. The region labeled ‘V’ is a void space. The relatively large standard deviation for the eutectic phase composition was due to this phase being present in small or narrow pockets and also because the eutectic phase is a composite of NiAl_3_ nanorods embedded in Al continuum.

**Fig. 11:**
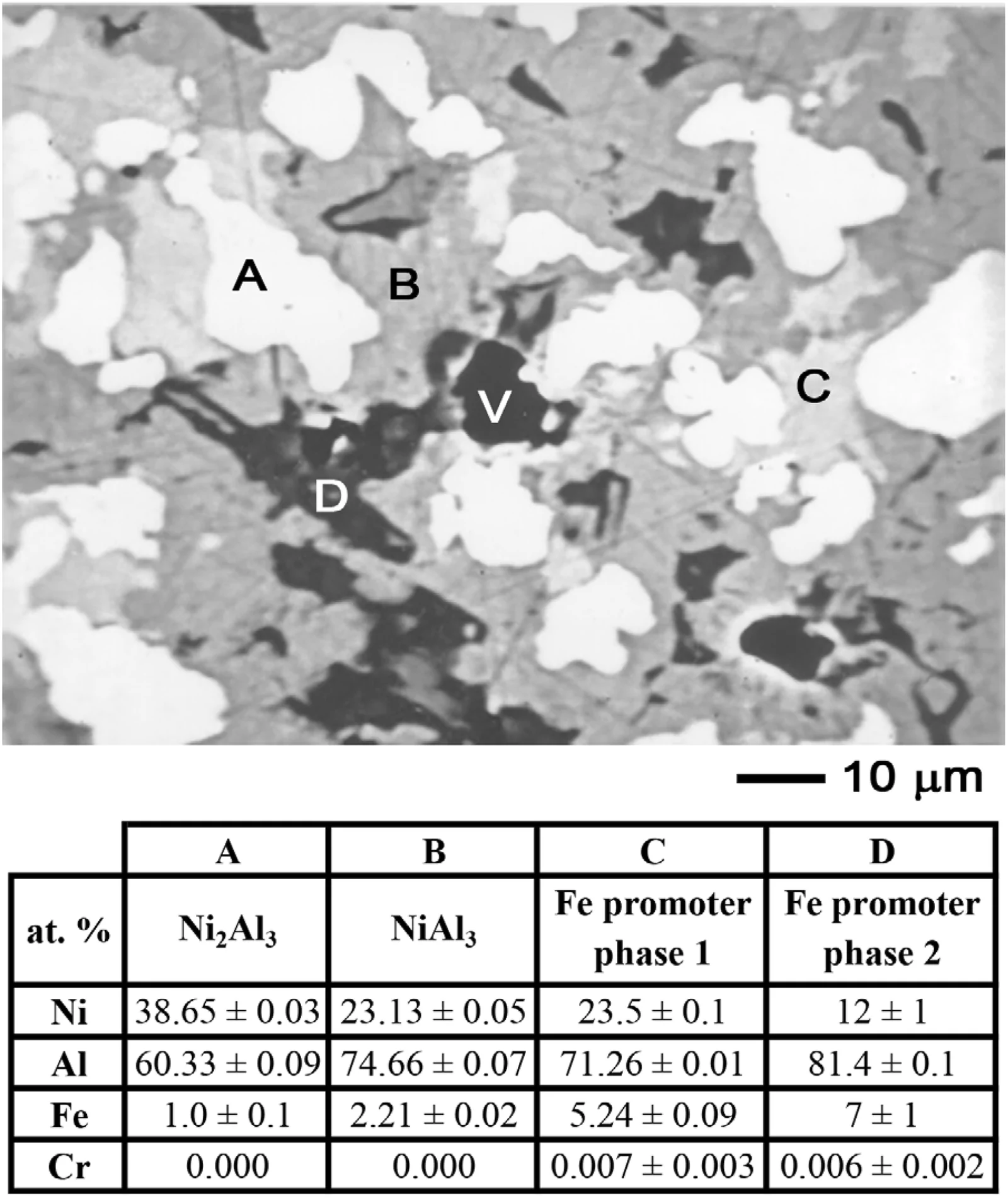
Backscattered SEM micrograph and electron microprobe results for the 4 wt% Fe promoted Ni–Al RANEY® alloy. The phase compositions are given in atomic percentage. This alloy may have also contained small amounts of eutectic phase (dark black regions), whose composition was not reliably analyzed due to its small volume and being percolated with the promoter phase. The region labeled ‘V’ is a void space.

**Fig. 12:**
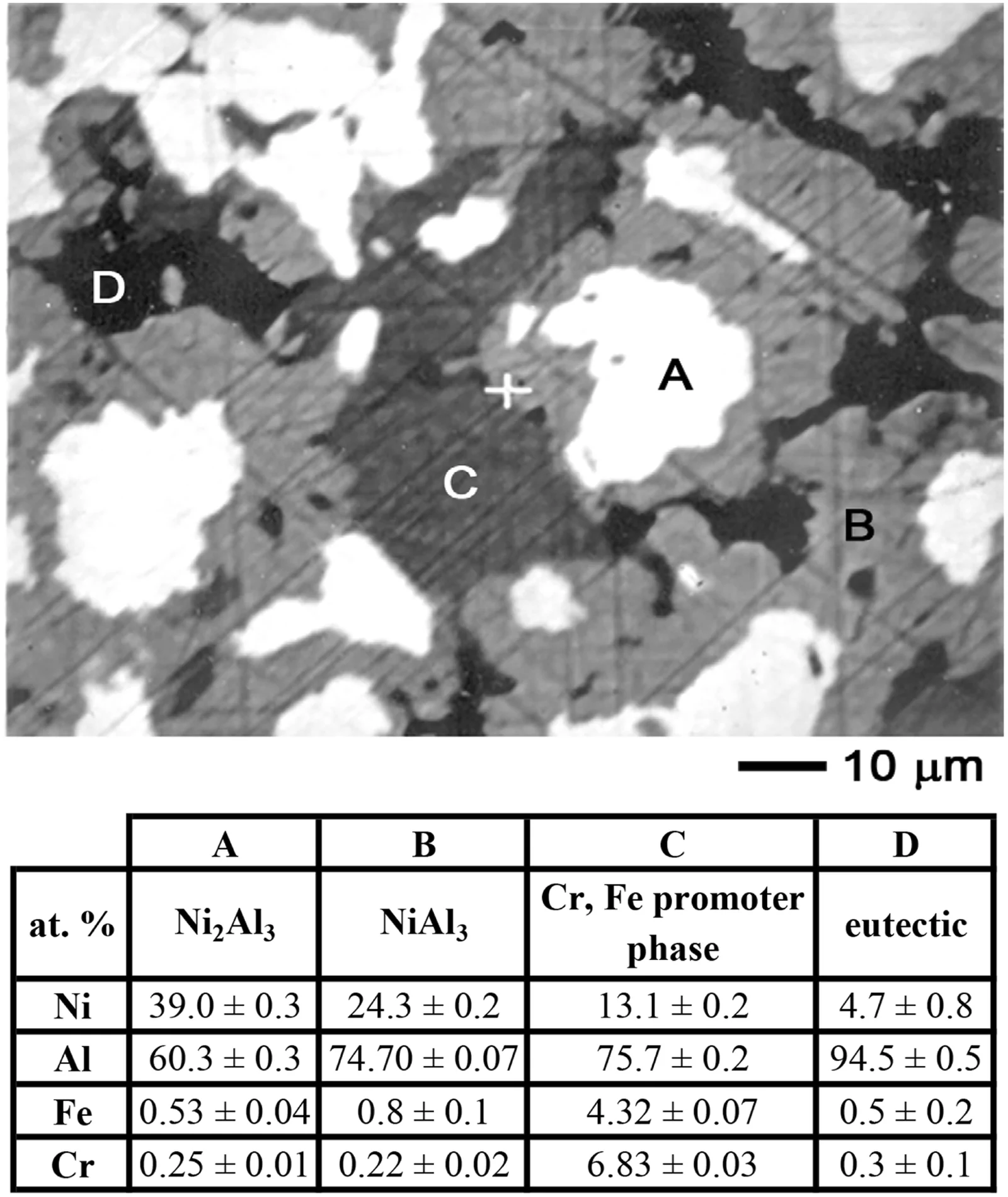
Backscattered SEM micrograph and electron microprobe results for the 1.5 wt% Fe, 1.5 wt% Cr doubly-promoted Ni–Al RANEY® alloy. The phase compositions are given in atomic percentage.

Examining these micrographs and electron microprobe data, the following trends are evident. The unpromoted alloy contained only Ni_2_Al_3_, NiAl_3_, and eutectic phases.

Alloys promoted with chromium only contained four phases: Ni_2_Al_3_, NiAl_3_, a ‘Cr promoter phase’, and eutectic. The ‘Cr promoter phase’ contained 11–14 at% Cr.

The Fe, Cr doubly-promoted alloy also contained four phases with the ‘Cr, Fe promoter phase’ containing 4.3 at% Fe and 6.8 at% Cr. The combined (Fe + Cr) at% in this promoter phase is practically identical to the Cr at% in the ‘Cr promoter phase’ of the Cr-only promoted alloys.

The 1 wt% Fe promoted alloy also contained four phases with the ‘Fe promoter phase 2’ containing 4 at% Fe and practically no Cr. The 2 wt% Fe promoted alloy contained five phases: Ni_2_Al_3_, NiAl_3_, ‘Fe promoter phase 1’ containing ∼4 at% Fe, ‘Fe promoter phase 2’ containing ∼5 at% Fe, and eutectic. In the 4 wt% Fe promoted alloy, the Ni_2_Al_3_, NiAl_3_, ‘Fe promoter phase 1’ containing ∼5 at% Fe, and ‘Fe promoter phase 2’ containing ∼7 at% Fe were abundant. The 4 wt% Fe promoted alloy may have also contained small amounts of eutectic phase (dark black regions), whose composition was not reliably analyzed due to its small volume and being percolated with the promoter phase.

When increasing the amount of Fe or Cr promoter, the volume fractions of the promoter-rich phases increased.

The grain structure in these micrographs indicate that Ni_2_Al_3_ solidified first, followed by ‘Fe promoter phase 1’ (when present in the Fe-only promoted alloys), followed by NiAl_3_, followed by either ‘Cr promoter phase’ or ‘Cr, Fe promoter phase’ (in the Cr containing alloys), followed by ‘Fe promoter phase 2’ (in the Fe-only promoted alloys), and finally eutectic. This can be inferred from which phases filled the spaces (including crevices) left over from the earlier solidified phases.

### 2.3 Electron microprobe of the commercial-grade Grace RANEY® Ni alloys

Electron microprobe analysis was also performed on the commercial-grade RANEY® nickel alloys that are manufactured by W.R. Grace & Co. RANEY® nickel is registered trademark of W.R. Grace & Co. The unpromoted Grace alloy is approximately 50 wt% Ni and 50 wt% Al. Alloys containing three levels of Fe-only promotion were studied: 0.9 wt% Fe, 2.0 wt% Fe, and 3.9 wt% Fe. The doubly-promoted Grace alloy contained 1.3 wt% Fe and 1.4 wt% Cr. The balance of each of these promoted alloys was approximately 48–50 wt% Ni and approximately 48–50 wt% Al.

Since these alloys were already present in a powdered form, we used a hydraulic press to prepare a pellet. This pellet was then mounted in bakelite and subsequently sanded and polished to produce a smooth surface. Electron microprobe analysis was then performed to analyze the compositions of the various alloy phases as shown in Table 2. However, due to the powdered structure, no optical or SEM micrographs are available.

**Table 2:** Electron microprobe analysis of commercial-grade Grace RANEY® Ni alloys. The compositions are given in atomic percentage. The relatively large standard deviation for the eutectic phase composition was due to this phase being present in small or narrow pockets and also because the eutectic phase is a composite of NiAl_3_ nanorods embedded in Al continuum

Alloy	Phase	Ni	Al	Fe	Cr
Unpromoted	Ni_2_Al_3_	38.8 ± 0.4	61.2 ± 0.4	0.03 ± 0.02	0.004 ± 0.004
Unpromoted	NiAl_3_	25.2 ± 0.3	74.7 ± 0.3	0.12 ± 0.02	0.009 ± 0.001
Unpromoted	Eutectic	7 ± 4	93 ± 4	0.06 ± 0.03	0.02 ± 0.01
0.9 wt% Fe	Ni_2_Al_3_	39.1 ± 0.2	60.8 ± 0.2	0.18 ± 0.02	0.000
0.9 wt% Fe	NiAl_3_	24.8 ± 0.4	74.6 ± 0.1	0.6 ± 0.2	0.004 ± 0.004
0.9 wt% Fe	Fe promoter phase 2	13.8 ± 0.1	82.8 ± 0.4	3.4 ± 0.3	0.006 ± 0.006
0.9 wt% Fe	Eutectic	4 ± 2	95 ± 2	0.13 ± 0.07	0.07 ± 0.02
2 wt% Fe	Ni_2_Al_3_	39.9 ± 0.3	59.7 ± 0.3	0.43 ± 0.02	0.008 ± 0.008
2 wt% Fe	NiAl_3_	24.1 ± 0.6	75.1 ± 0.3	0.8 ± 0.3	0.005 ± 0.004
2 wt% Fe	Fe promoter phase 2	13.3 ± 0.7	81.3 ± 0.1	5.4 ± 0.8	0.001 ± 0.001
2 wt% Fe	Eutectic	4 ± 2	96 ± 2	0.3 ± 0.2	0.12 ± 0.02
3.9 wt% Fe	Ni_2_Al_3_	37.0 ± 0.1	61.85 ± 0.05	1.19 ± 0.05	0.000
3.9 wt% Fe	NiAl_3_	23.2 ± 0.4	75.0 ± 0.2	1.8 ± 0.6	0.008 ± 0.001
3.9 wt% Fe	Fe promoter phase 1	24.5 ± 0.3	70.5 ± 0.3	5.00 ± 0.04	0.007 ± 0.007
3.9 wt% Fe	Fe promoter phase 2	12.4 ± 0.3	81.7 ± 0.4	5.90 ± 0.07	0.003 ± 0.003
Fe,Cr-promoted	Ni_2_Al_3_	37.55 ± 0.07	61.59 ± 0.04	0.60 ± 0.07	0.26 ± 0.04
Fe,Cr-promoted	NiAl_3_	24.6 ± 0.3	74.4 ± 0.3	0.87 ± 0.05	0.070 ± 0.001
Fe, Cr-promoted	Cr, Fe promoter phase	15 ± 1	75.4 ± 0.2	3.6 ± 0.7	6.2 ± 0.9

Examining Table 2, the following trends are apparent. The Grace and Purdue alloys having similar (although not identical) compositions were composed of nearly the same set of phases, with a few minor exceptions. Since the nickel content of the Grace alloy (∼48–50 wt% Ni) was higher than that of the Purdue alloy (42 wt% Ni), this reduced the amount of eutectic phase present in the Grace alloys compared to the Purdue alloys. Consequently, we were not able to obtain reliable electron microprobe results for the extremely small portion of eutectic phase present in the 3.9 wt% Fe and doubly-promoted Grace alloys. This behavior was similar to the 4 wt% Fe Purdue alloy, but differed from that of the doubly-promoted Purdue alloy.

The ‘Fe promoter phase 1’ was observed in the 3.9 wt% Fe Grace alloy, but not in any of the other four Grace alloys. As explained in the preceding section, the ‘Fe promoter phase 1’ was present in the 2 wt% Fe and 4 wt% Fe Purdue alloys, but not in the other Purdue alloys.

The composition of each phase was strikingly similar in the Grace and Purdue alloys. This was true not only for the Ni_2_Al_3_, NiAl_3_, and eutectic phases but also for each of the promoter-rich phases.

## 3. Discussion of the promoter-rich phase structures

### 3.1 Fe promoter-rich phases 1 and 2

The Al–Ni–Fe ternary system was reviewed by Raghavan.^76–79^ Zhang *et al.* used the CALPHAD (CAlculation of PHAse Diagrams) method together with calorimetry experiments and first-principles modeling to study its thermodynamic phase equilibria properties.^80,81^

Of particular interest, Chumak *et al.*^82^ published a liquidus surface projection of the Al–Ni–Fe system between 50 and 100 at% Al, which is reproduced in Fig. 13. In a liquidus surface projection, each point on the diagram corresponds to the composition of the melt (*i.e.*, liquid phase). The diagram is divided into area domains corresponding to which solid phase is the first to solidify from the melt. Lines mark the boundary of liquid compositions for which two solids can precipitate. Arrows mark the direction of decreasing temperature. Binary eutectic (or eutectoid) reactions are denoted by ‘e’ and ternary eutectic (or eutectoid) reactions by ‘E’. Binary peritectic (or peritectoid) reactions are denoted by ‘p’ and ternary peritectic (or peritectoid) reactions by ‘P’. Other multiphase reactions are denoted by ‘U’.

**Fig. 13:**
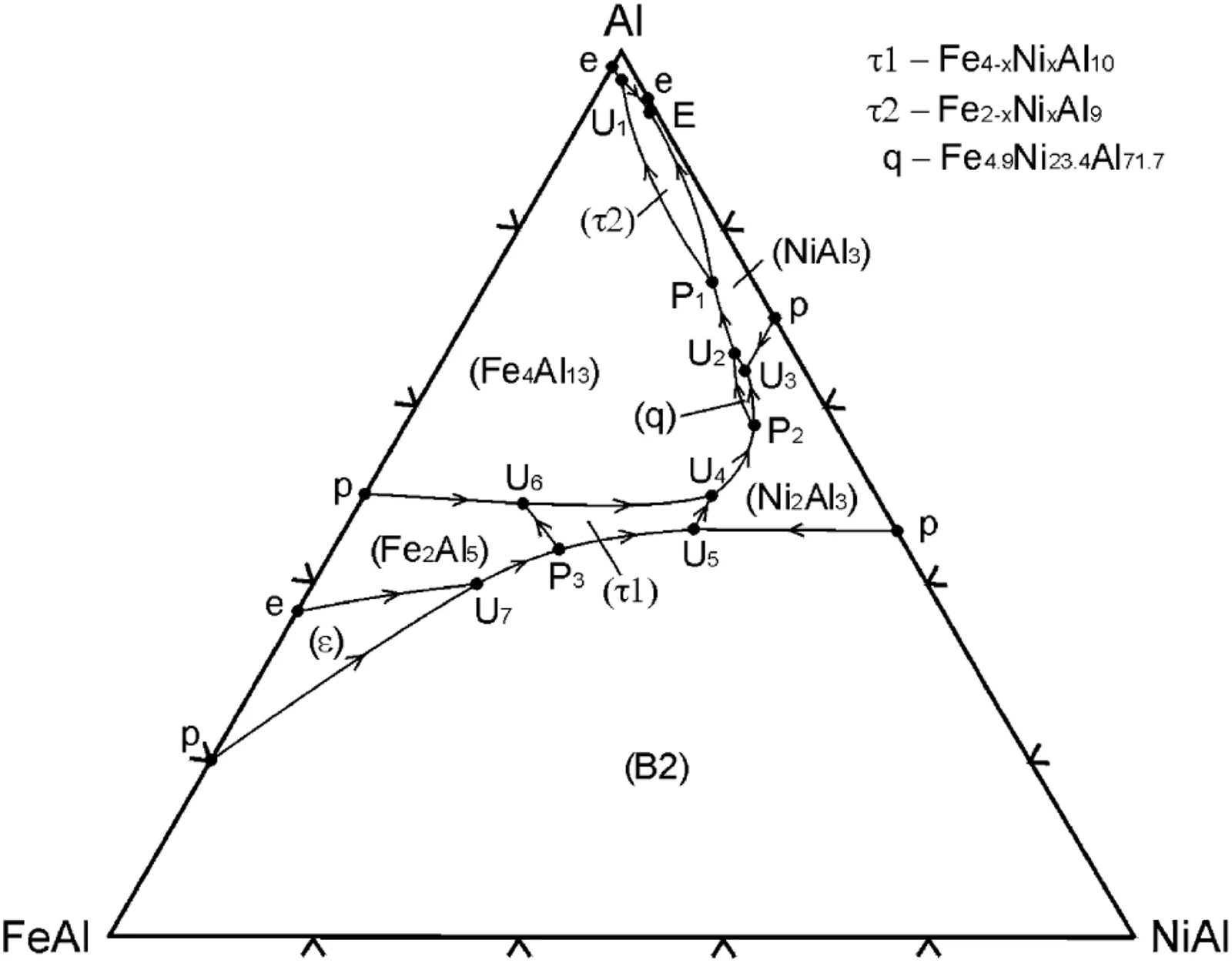
Liquidus surface projection of Al–Ni–Fe system between 50 and 100 at% Al. Please see the main text for a description of how to read this diagram. Reproduced with permission from I. Chumak *et al.*, *Intermetallics*, 2007, **15**, 1416–1424. Copyright 2007 Elsevier.

Our 4 wt% Fe, 42 wt% Ni, 54 wt% Al alloy corresponds to a molar composition of 2.6 at% Fe, 25.6 at% Ni, 71.8 at% Al. By examining Chumak *et al.*’s liquidus surface projection diagram, the following predictions are made for this alloy:

(1) Crystalline Al_3_Ni_2_ is the first phase to precipitate from the melt.

(2) Taking into consideration the relatively low solubility of Fe in Al_3_Ni_2_,^82,83^ the liquid will become enriched in Fe as Al_3_Ni_2_ precipitates. This causes the melt to move in the direction of increasing Fe and decreasing Ni content until it reaches one of the reactions that produces the decagonal quasicrystal phase (marked ‘(q)’ on Chumak's liquidus surface projection diagram). The decagonal quasicrystal phase is stable between approximately 850 °C and 930 °C.^82,83^

(3) Crystalline Al_3_Ni will be produced by the peritectic reaction L + Al_3_Ni_2_ → Al_3_Ni. This occurs around 854 °C (see Fig. 1). Below this temperature, Al_3_Ni can also precipitate directly from the melt.

(4) The Al_9_Ni_2−*y*_Fe_*y*_ crystalline phase is not stable above 809 °C.^82^ Below this temperature, it precipitates from the melt of appropriate composition range.^82^

(5) Finally, the binary (Al/Al_3_Ni at 640 °C as shown in Fig. 1) or ternary (Al/Al_3_Ni/Al_9_Ni_2−*y*_Fe_*y*_ at 634 °C from ref. 64) eutectic will precipitate. As shown in Fig. 13, the binary and ternary eutectics are close to each other in composition and temperature.

First, we discuss evidence that neither ‘Fe promoter phase 1’ nor ‘Fe promoter phase 2’ were the Al_13_Fe_4_ crystalline phase. Chumak *et al.* state that ‘the maximal solubility of Ni in Fe_4_Al_13_ is 11 at%’.^82^ This also agrees with the Al–Ni–Fe phase diagram at 850 °C by Lemmerz *et al.*^83^ For the ternary Al–Ni–Fe system, this means that the Fe content in this phase is always ≥12.5 at%. This minimum Fe content of 12.5 at% would occur if this phase contained 11 at% Ni, and all Ni atoms replaced Fe sites. Neither ‘Fe promoter phase 1’ nor ‘Fe promoter phase 2’ contained Fe concentrations as high as 10 at% let alone 12.5 at%. Accordingly, neither ‘Fe promoter phase 1’ nor ‘Fe promoter phase 2’ were the Al_13_Fe_(4−*x*)_Ni_*x*_ phase.

Why was the Al_13_Fe_(4−*x*)_Ni_*x*_ crystalline phase not observed in our alloys? Apparently, the amount of Fe in the melt was kept sufficiently low so as not to produce the Al_13_Fe_(4−*x*)_Ni_*x*_ phase during solidification. Examining Fig. 13, the Al_13_Fe_(4−*x*)_Ni_*x*_ phase is expected to precipitate at slightly higher Fe concentrations in the melt than are needed to precipitate the ‘q’ (decagonal quasicrystal) and ‘*τ*_2_’ (Al_9_Ni_2−*y*_Fe_*y*_) ternary phases.

We now discuss evidence showing that ‘Fe promoter phase 1’ is the decagonite quasicrystal phase, and ‘Fe promoter phase 2’ is the Al_9_Ni_2−*y*_Fe_*y*_ (*τ*_2_) crystalline phase. The decagonal quasicrystal phase can persist as a metastable phase below 850 °C if the alloy is rapidly cooled (‘quenched’).^82,83^ This quenching causes the phase structure to be frozen. This explains why the decagonal phase could be observed in our Fe-promoted alloys even at room temperature.

In 1989, Tsai *et al.* reported a decagonal quasicrystal phase for a rapidly quenched Al_75_Ni_10_Fe_15_ alloy.^84^ However, that composition was not obtained through phase equilibration. Subsequent synthetic studies provided an equilibrated composition near Al_71–72_Ni_24–23_Fe_5_.^82,83,85^ Subsequently, this decagonal phase with composition Al_71_Ni_24_Fe_5_ was discovered in a natural sample; specifically, a fragment of the Khatyrka meteorite.^86–88^ This new mineral was given the name decagonite.^86,88^

Fig. 14 shows the XRD powder pattern of decagonite. Decagonal quasicrystals have a layered structure that is periodic in the direction perpendicular to the decagonal symmetry. They behave as 2D quasi-crystals within the layers containing decagonal symmetry. Consequently, decagonal quasi-crystals have XRD powder patterns having one large peak corresponding to the axial periodicity and a few small peaks corresponding to the long-range order of the 2D quasi-crystal layers. In spite of lacking strict periodicity within the quasi-crystal layers, the XRD powder patterns have sharp peaks. The reason for this surprising behavior is recounted in several reviews and perspectives on quasi-crystals.^89–91^ Since their discovery in 1984,^92,93^ more than 100 quasi-crystals have been subsequently discovered.^94^

**Fig. 14:**
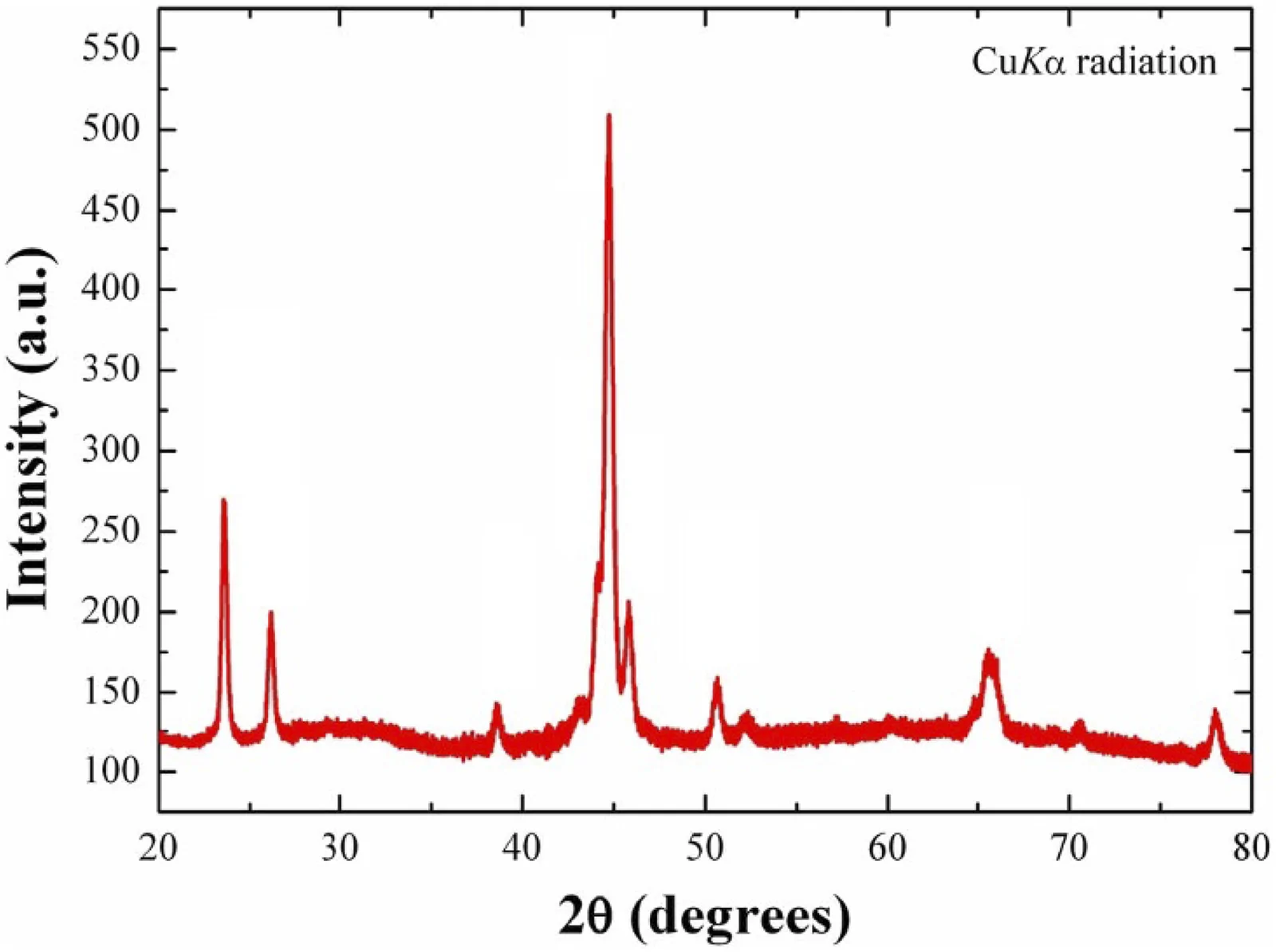
X-ray powder diffraction pattern for natural Al_71_Ni_24_Fe_5_ (aka decagonite). Reproduced with permission from L. Bindi *et al.*, *Sci. Rep.*, 2015, **5**, 9111. Copyright 2015 Nature Portfolio. Licensed under CC-BY 4.0.

At first, one might postulate that it would be easy to formulate a periodic crystal structure that could mimic a quasi-crystal's X-ray diffraction pattern. According to this hypothesis, one could simply replace the quasi-crystal with a periodic crystal having a suitable arrangement of atoms to produce a similar X-ray diffraction pattern. This has been tried by talented chemists, including Linus Pauling.^95,96^ Although this gets one close to the quasi-crystal's X-ray diffraction pattern, these periodic crystals always represent an approximation and do not exactly reproduce the quasi-crystal's X-ray diffraction pattern.^97^ Indeed, not only can such approximants be produced computationally, but they are also found in the real world. For example, the mineral proxidecagonite, which was also found in a fragment of the Khatyrka meteorite, is a periodic crystal of composition Al_34_Ni_9_Fe_2_ that is a periodic approximant to decagonite.^98^

However, there is ample evidence that ‘Fe promoter phase 1’ is the decagonite quasicrystal and not the proxidecagonite periodic crystal. First, the Al–Ni–Fe phase equilibration studies of Lemmerz *et al.*^83^ and Chumak *et al.*^82^ have identified the decagonite quasicrystal as the corresponding equilibrium phase in the 850–930 °C temperature window. Notably, Lemmerz *et al.* provided the corresponding measured XRD powder pattern for the decagonite quasi-crystal, and this closely matches Bindi *et al.*'s XRD powder pattern for the decagonite quasi-crystal found in the Khatyrka meteorite.^83,87^ Second, our electron microprobe analysis results provide phase compositions that align with decagonite rather than with proxidecagonite. Specifically, proxidecagonite contains 75.6 at% Al and 20.0 at% Ni with the rest Fe, while decagonite contains 71 at% Al and 24 at% Ni with the rest Fe. Since our ‘Fe promoter phase 1’ contained 71 at% Al and 24 at% Ni with the rest Fe, it is clearly decagonite and not proxidecagonite.

The Al_9_Ni_2−*y*_Fe_*y*_ (*τ*_2_) crystalline phase has the Co_2_Al_9_-type (Pearson symbol mP22, space group *P*2_1_/*c*) of monoclinic structure. Chumak *et al.* give lattice parameter values of *a* = 6.2406, *b* = 6.2993, *c* = 8.5992 Å, and *β* = 95.129° for the composition Al_9_Ni_1.3_Fe_0.7_.^79,82^ This composition corresponds to 81.8 at% Al, 11.8 at% Ni, and 6.4 at% Fe. As shown in Fig. 11, this is nearly identical to the ‘Fe promoter phase 2’ composition (81.4 at% Al, 12 at% Ni, and 7 at% Fe) we measured for the 4 wt% Fe alloy. As shown in Fig. 9 and 10, the Al content in ‘Fe promoter phase 2’ remained nearly constant, while the Fe content decreases slightly to 5.4 at% (in the 2 wt% Fe alloy) and 4.1 at% (in the 1 wt% Fe alloy). As shown in Table 2, the Grace alloys had similar (albeit not strictly identical) compositions of ‘Fe promoter phase 2’ compared to the Purdue alloys. The Al_9_Ni_2−*y*_Fe_*y*_ promoter-rich phase has been previously reported in RANEY® Al–Ni alloys.^63,99^

Finally, we show our measured XRD powder patterns are consistent with these phase assignments. Fig. 15 shows the XRD powder patterns for Al, Al_3_Ni, Al_3_Ni_2_, and Al_9_Fe_2_ crystals. These were computed as follows. We downloaded the crystal structure geometries from the Materials Project online database: Al (MP # 134), Al_3_Ni (MP # 622209), Al_3_Ni_2_ (MP # 1057), and Al_9_Fe_2_ (MP # 1191778).^100,101^ The XRD powder patterns were then computed in Vesta software for a Cu Kα X-ray radiation source using the default settings.^102^ For the Al-eutectic, Al_3_Ni, and Al_3_Ni_2_ phases, the peak positions were also compared to previously reported experimental XRD powder pattern peak positions and found to be consistent.^7,8,53,67,134^

**Fig. 15:**
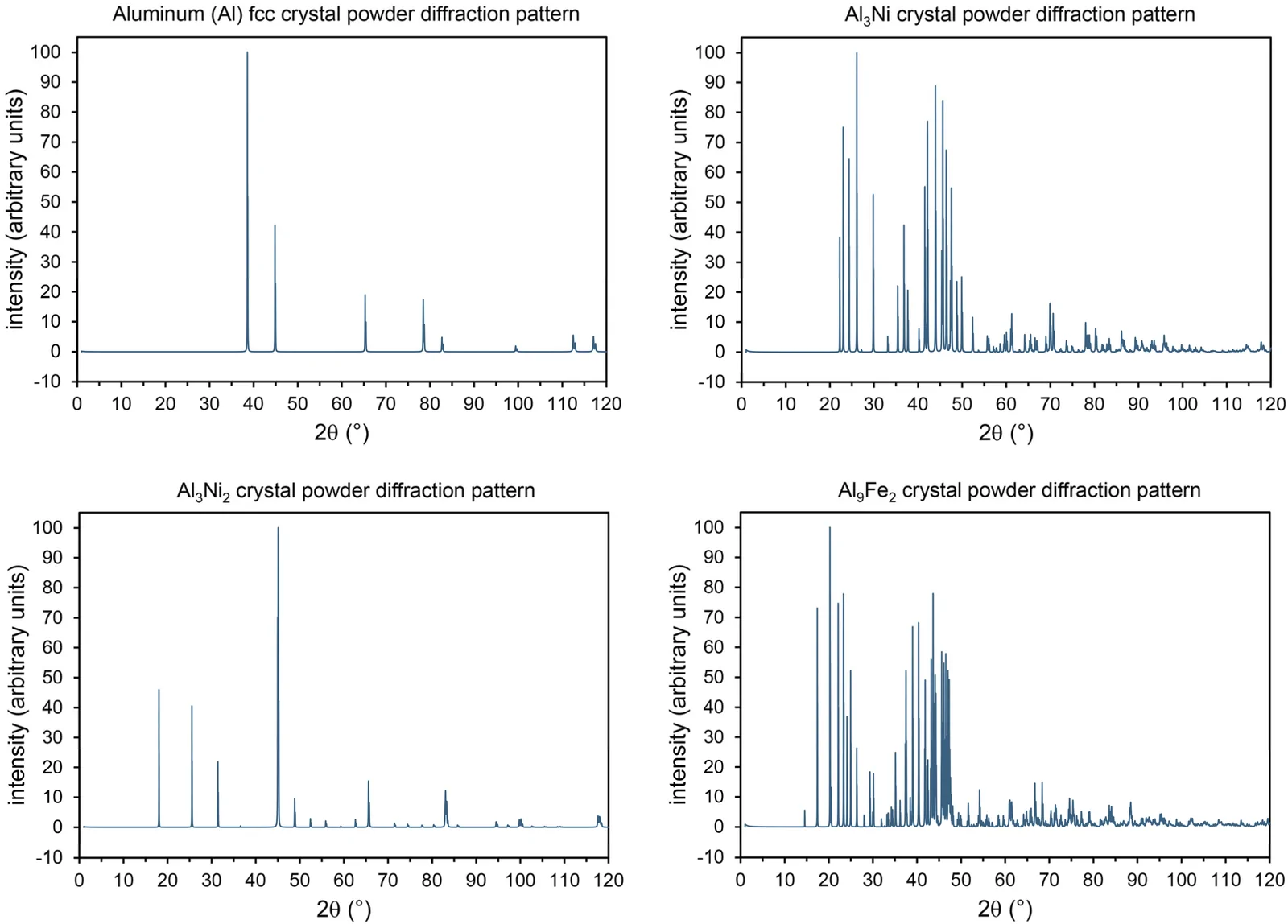
Computed X-ray powder diffraction patterns for Al, Al_3_Ni, Al_3_Ni_2_, and Al_9_Fe_2_ crystal structures. Please see the main text for a description of how these were computed.

Fig. 16 shows the XRD powder patterns of the unpromoted and 4 wt% Fe promoted 42 wt% Ni Purdue alloys. Because the eutectic phase is a mixture of Al (high percentage) and NiAl_3_ (small percentage), the peaks marked ‘Al’ largely correspond to the eutectic. As shown in Fig. 16, there are small visible peaks for the Fe promoter phase 1 and Fe promoter phase 2, which are marked accordingly. The main peaks for the promoter-rich phases were for 2*θ* values near 51° and 23° for the quasi-crystalline phase, and for 39° and 20° for the Al_9_Ni_2−*y*_Fe_*y*_ phase.

**Fig. 16:**
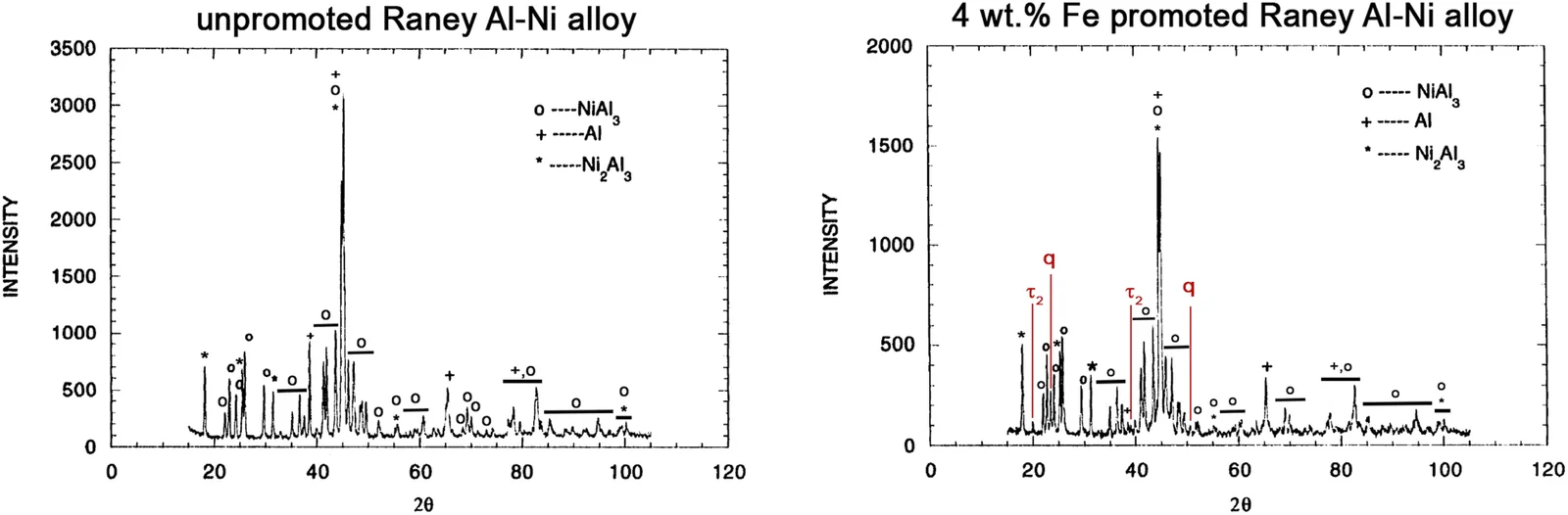
XRD pattern of unpromoted RANEY® Al–Ni alloy (left panel) and 4 wt% Fe promoted alloy (right panel). The corresponding phase(s) for each peak are marked. In the right-hand panel, the Fe promoter-rich phases are marked in red. The quasi-crystalline phase (Al_71–72_Ni_24–23_Fe_5_) is marked q, and the Al_9_Ni_2−*y*_Fe_*y*_ phase is marked *τ*_2_.

### 3.2 Cr promoter-rich phase

Examining Fig. 6–8, the composition of ‘Cr promoter phase’ is approximately Al_77.5_Cr_12_Ni_10.5_. A review of the literature showed that previous studies identified Al–Cr–Ni ternary phases near this composition. These will now be discussed.

Weitzer *et al.*^103^ and Grushko *et al.*^104^ constructed isothermal phase diagrams for the Al–Ni–Cr system around 700 °C. Both of these studies annealed the samples around 700 °C for a long time to equilibrate the phases.^103,104^ They identified a phase (called *τ*_2_ in ref. 103 and *ϕ* in ref. 104) near the above stated composition. To avoid confusion with the unrelated Fe-containing *τ*_2_ phase discussed in the prior section, we will refer to this phase as the *ϕ* phase. Both studies measured the XRD powder pattern for this crystal phase (see Fig. 17), and this XRD powder pattern agreed between these two studies.^103,104^ This confirmed that the same phase was observed in both studies. The lattice parameters for this monoclinic crystal phase are *a* = 13.40 Å, *b* = 12.55 Å, *c* = 12.55 Å, with *β* ≈ 100°, *α* = *γ* = 90°.^104^

**Fig. 17:**
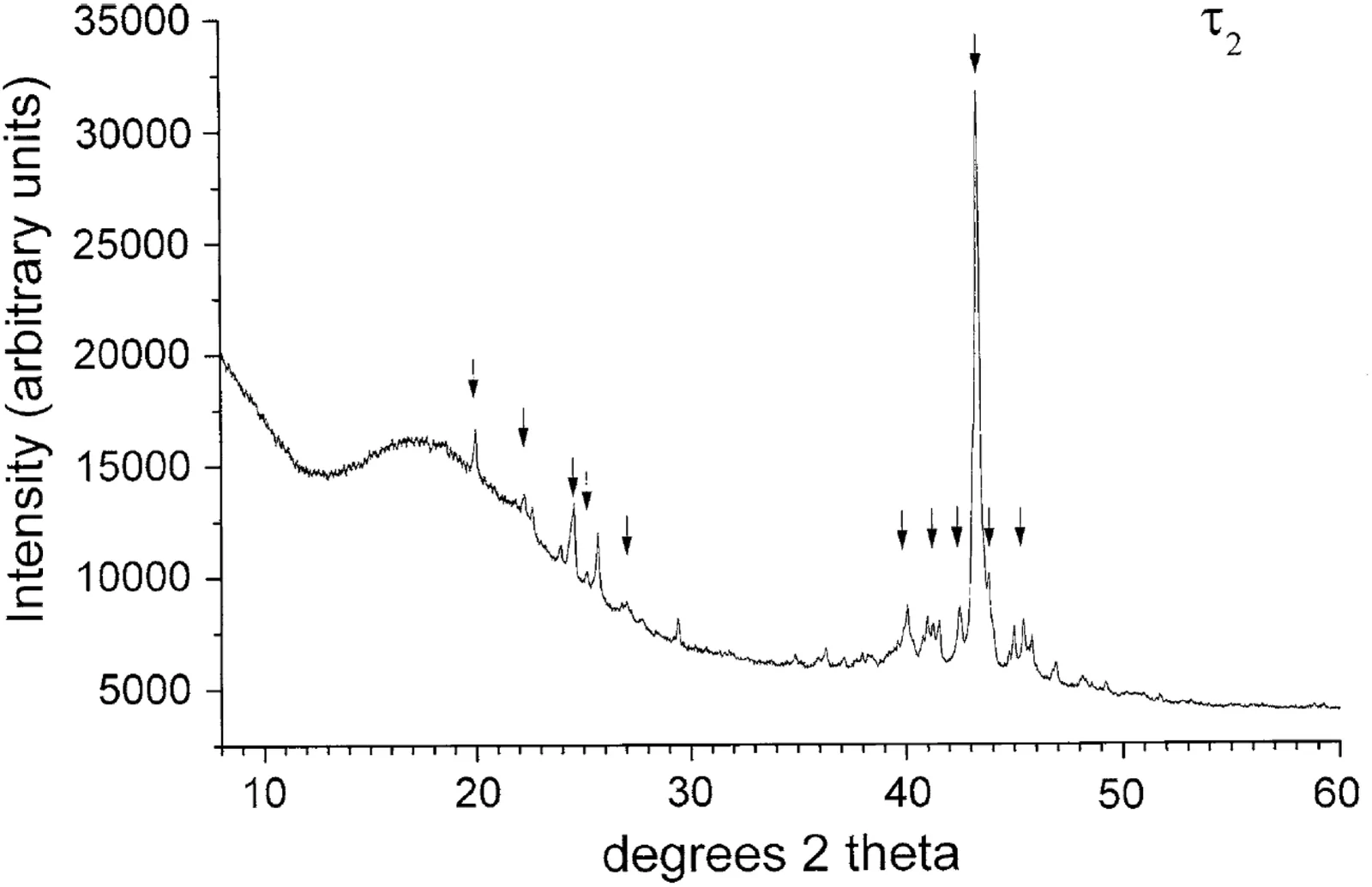
X-ray powder diffraction pattern of alloy Al_80_Cr_10_Ni_10_ annealed at 750 °C showing the rather simple powder pattern of the predominant *ϕ* (aka *τ*_2_) phase (peaks marked by arrows). The remaining peaks are due to minor amounts of Al_3_Ni. Reproduced with permission from F. Weitzer *et al.*, *Metall. Mater. Trans. A*, 2008, **39A**, 2363–2369. Copyright 2008 The Minerals, Metals & Materials Society and ASM International.

A key consideration is that the composition of ‘Cr promoter phase’ remained consistently close to Al_77.5_Cr_12_Ni_10.5_ for the 1 wt%, 2 wt%, and 4 wt% Cr alloys in our study. This pinpoints the relevant phase composition on the ternary Al–Ni–Cr phase diagram. At this composition, only one equilibrium phase is present on the 700 °C (ref. 103 and 104) and 800 °C (ref. 104) ternary Al–Ni–Cr phase diagrams, and that is the *ϕ* phase. Notably, these ternary phase diagrams showed that the *ϕ* phase forms with this specific composition even as it forms across a wide window of bulk alloy compositions. This is possible, because it forms in conjunction with other phases such as Al_3_Ni. Compositions between the *ϕ* phase composition and the Al_3_Ni phase composition segregate into these two phases of defined compositions.

Examining the ternary Al–Ni–Cr phase diagrams reported by Weitzer *et al.*^103^ (at 700 °C), Grushko *et al.*^104^ (at 700 °C and 800 °C), and Grushko *et al.*^135^ (at 900 °C, 1000 °C, and 1150 °C) shows the *ϕ* phase starts to precipitate from the melt between 800 °C and 900 °C. That portion of the phase diagram is liquid at temperatures ≥900 °C.

This is consistent with our optical and SEM micrographs that indicate the ‘Cr promoter phase’ begins to form above the 854 °C peritectic reaction between Al_3_Ni_2_ and the melt that produces the NiAl_3_ phase. This can be inferred directly, because some of the ‘Cr promoter phase’ borders the Al_3_Ni_2_ phase in our samples, and this would not be the case if it first started forming below the peritectic reaction producing the NiAl_3_ phase. (In that case, the NiAl_3_ phase would coat the surface of the Al_3_Ni_2_ phase thereby preventing any subsequently formed phases from bordering the Al_3_Ni_2_ phase in the optical and SEM micrographs.) Close examination of the microstructures of the ‘Cr promoter phase’ in Fig. 6 and 8 shows that this phase sometimes borders the eutectic, Al_3_Ni, and Al_3_Ni_2_ phase. This behavior is different from that of ‘Fe promoter phase 2’ which has eutectic and Al_3_Ni phases on its perimeter but not the Al_3_Ni_2_ phase (see Fig. 9–11). This suggests that ‘Cr promoter phase’ begins to form at a higher temperature than ‘Fe promoter phase 2’. So, we have strong experimental evidence that ‘Cr promoter phase’ formed in our samples at approximately the same temperature as the *ϕ* phase was reported to form in the literature.

According to Grushko *et al.*,^104^ the *ϕ* crystal phase undergoes incongruent melting (*e.g.*, involving a peritectic reaction) around 835 °C.^104^ This is slightly lower than the peritectic temperature of 854 °C (see Fig. 1) forming the Al_3_Ni phase. However, there is a disagreement between the studies of Weitzer *et al.*^103^ and Grushko *et al.*^104^ on this subject. Specifically, Weitzer *et al.* claim there is a triangular region in the 700 °C isothermal section in which the crystal phases *ϕ*, Al_3_Ni, and Al_3_Ni_2_ are co-produced, and an adjacent triangular region where the crystal phases *ϕ*, Al_3_Ni, and *θ*-Al_45_Cr_7_ are co-produced. On the other hand, Grushko *et al.*^104^ claimed that there is a two-phase region in which *ϕ* and Al_3_Ni (but not any Al_3_Ni_2_) are co-produced, and an adjacent triangular region where the crystal phases *ϕ*, Al_3_Ni, and *θ*-Al_45_Cr_7_ are co-produced.

Since we did not observe any *θ*-Al_45_Cr_7_ phase being produced in our Cr-only promoted RANEY® nickel alloys, this implies that the ‘Cr promoter phase’ was produced alongside the Al_3_Ni and the melt (which solidified into the eutectic phase a lower temperature). Al_3_Ni_2_ precipitates at much higher temperatures (see Fig. 1) than ‘Cr promoter phase’. Since both the ‘Cr promoter phase’ and Al_3_Ni sometimes share a border with the Al_3_Ni_2_ phase (see Fig. 8), and Al_3_Ni is formed *via* a peritectic reaction between Al_3_Ni_2_ and the melt, this suggests that at least some of the ‘Cr promoter phase’ forms before all of the Al_3_Ni phase has finished forming.

Earlier studies observed related phases near the same Al–Cr–Ni ternary composition.^64,105^ Rosell-Laclau *et al.* observed a phase called ‘*λ*’ that when annealed had the composition Al_76.8_Cr_12.8_Ni_10.4_.^64^ This phase was described as ‘the orthorhombic polytope of *ϕ*’ with one of the crystal planes being shared between the two crystal structures.^64^ However, Rosell-Laclau *et al.* go on to state that a triclinic superstructure was also observed and that ‘at least two other structures are directly related to this structure of the *λ* phase …’.^64^ They also mention crystal local twinning to form various polytopes of the *ϕ* and *λ* phases.^64^ This observation of various superlattice reflections and local twinning could indicate that their samples were not fully equilibrated during annealing, and this caused various crystal polytopes to be formed. Although Compton *et al.*^106^ tentatively assigned (*i.e.*, without any detailed structural characterization or XRD spectra) some of their results to this ‘*λ*’ phase, the later studies of Grushko *et al.*^104^ and Weitzer *et al.*^103^ did not observe this ‘*λ*’ phase. Instead, Grushko *et al.*^104^ and Weitzer *et al.*^103^ observed the *ϕ* phase at this composition in their annealed samples.

Recently, Amini *et al.* observed a decagonal quasicrystal (d-QC) phase in an alloy of overall composition Al_72_Cr_15_Ni_13_ annealed at 1035 °C after mechanical ball milling, which produced some Al_3_Ni_2_ phase alongside the d-QC phase.^107^ However, this d-QC phase was not produced in the samples annealed at 865 °C and lower temperatures.^107^ This d-QC phase and the *ϕ* crystal phase are ‘structural cousins’, because they have similar (but not identical) XRD powder patterns showing a largest main peak near a *d*-spacing value of 2.077 Å, surrounded by ∼5 peaks in the range 1.8–2.3 Å, plus a pair of peaks near 3.595 and 3.511 Å, *etc.*^103,104,107^ Some of the peak positions for the d-QC and *ϕ* crystal phase are not the same, which shows they are different phases.^103,104,107^ The X-ray Laue photographs show that d-QC is a quasicrystal with decagonal symmetry while the *ϕ* phase is a periodic crystal with translational symmetry.^104,107^ The *ϕ* phase is sometime referred to as a ‘decagonal approximant’.

The above studies show that several phase polytopes exist in the vicinity of the relevant composition of the ‘Cr promoter phase’. The studies of Grushko *et al.*^104^ and Weitzer *et al.*^103^ performed long duration annealing around 700 °C, which showed the ground-state (*i.e.*, low energy phase) at the relevant temperature range (around 700–835 °C) is the *ϕ* phase.

Fig. 18 shows the XRD powder pattern for our 4 wt% Cr promoted Al–Ni alloy. There is a small peak near 42.5° that is consistent with the main peak position (see Fig. 17) for the *ϕ* (aka *τ*_2_) phase. This peak was not present in the unpromoted and Fe-promoted alloys. Besides this small peak near 42.5°, no other new peaks were observed in the Cr-only promoted alloys. Together with the composition analysis discussed above, this indicates the Cr promoter-rich phase is the *ϕ* phase of approximate composition Al_77.5_Cr_12_Ni_10.5_.

**Fig. 18:**
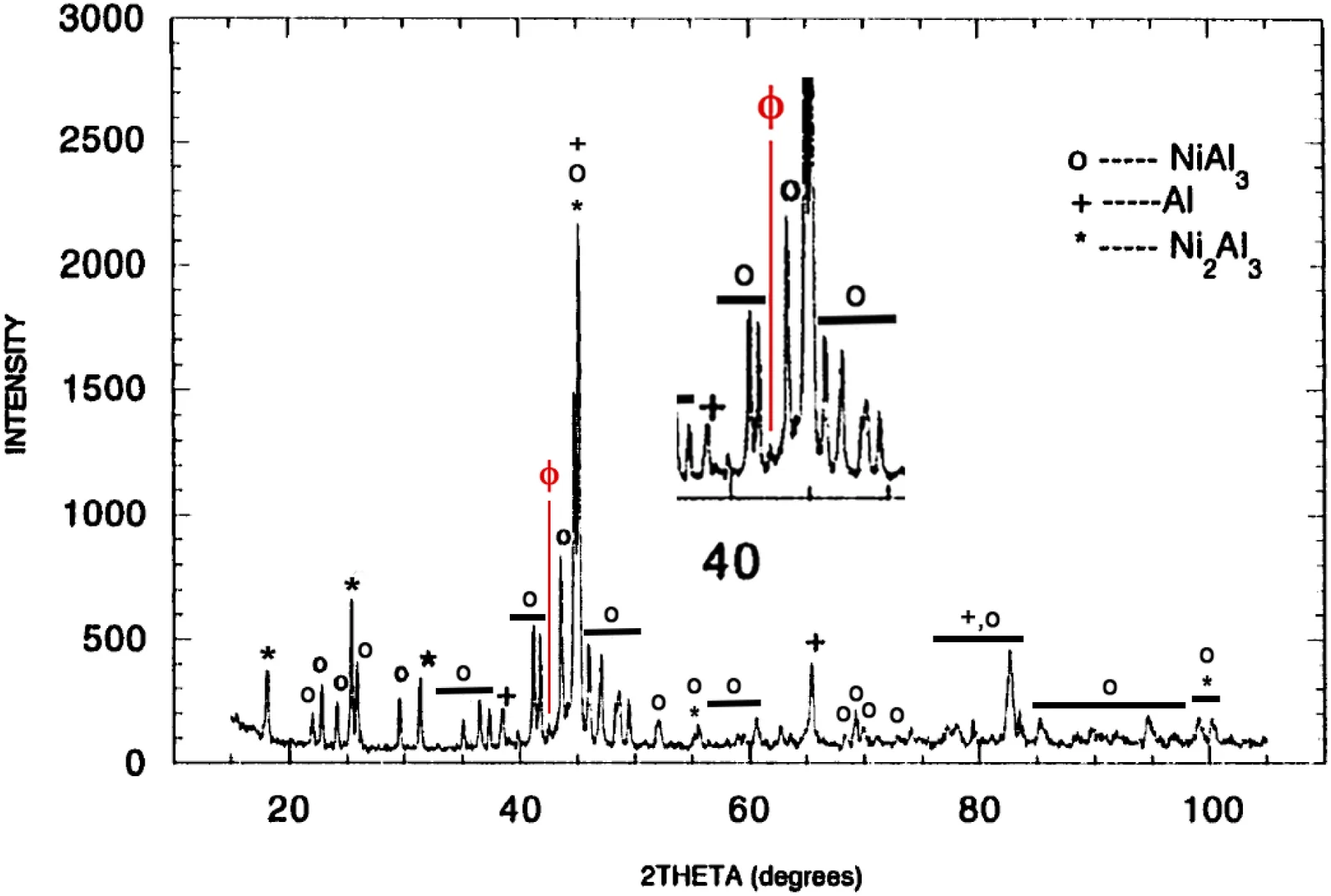
X-ray powder diffraction pattern of our 54 wt% Al, 42 wt% Ni, 4 wt% Cr alloy. The peak positions corresponding to various phases are labeled. The small peak near 42.5° for the Cr promoter-rich *ϕ* phase is marked in red. This region is enlarged in the inset.

This does not necessarily rule out the possibility that ‘Cr promoter phase’ is a non-equilibrium phase. It is possible that the ‘Cr promoter phase’ could be one of the closely related non-equilibrium polytopes described above, such as the ‘*λ*’ phase. These are ‘structural cousins’ of the *ϕ* phase.

### 3.3 Cr, Fe promoter-rich phase

Table 3 summarizes key properties of the promoter-rich phases in these alloys. As described in Sections 3.1 and 3.2 above, the Fe promoter-rich phases 1 and 2 and the Cr promoter-rich phase have been assigned structures. On the other hand, the structure of the ‘Cr, Fe promoter phase’ is not yet fully known.

**Table 3:** Key properties of the promoter-rich phases

Promoter-rich phase	Approximate chemical formula	Assignedstructure	Key structurereferences
Fe promoter phase 1	Al_71_Ni_24_Fe_5_	Decagonite quasicrystal (q)	82–85, and 87
Fe promoter phase 2	Al_9_Ni_(2−_*_y_*_)_Fe_*y*_	*τ*_2_ monoclinic crystal	82
Cr promoter phase	Al_77.5_Cr_12_Ni_10.5_	*ϕ* monoclinic crystal	103 and 104
Cr, Fe promoter phase	Al_75.5_Ni_13–14_Cr_6–7_Fe_4_	Unknown	108

To date, the Al–Ni–Cr–Fe quaternary phase diagram has not been completely mapped across all temperature and composition ranges.^108,109^ Moreover, it is not currently possible for us to make a definitive structural assignment for the ‘Cr, Fe promoter phase’. Although we are not currently equipped to perform such experiments, micro-XRD (which performs X-ray diffraction on a small spot) could potentially be used in future work to collect single-phase XRD data on the ‘Cr, Fe promoter phase’. X-ray crystallography with structural refinement on such micro-XRD data could then potentially be used to generate the 3D molecular structure of the ‘Cr, Fe promoter phase’.

Although the precise 3D molecular structure of the ‘Cr, Fe promoter phase’ is currently unknown to us, it is likely a ‘structural cousin’ of the following phases: ‘Cr promoter phase’ (*ϕ* phase discussed in the previous section), Al_13_Fe_4_ (see ref. 110 and 111), Al_13_Co_4_ (see ref. 112–114 for several different crystal structures), and decagonal quasicrystal (discussed in Sections 3.1 and 3.2 above). The Al_13_Fe_4_ and Al_13_Co_4_ periodic crystal phases have decagonal pseudosymmetry that is an approximant to quasi-crystalline decagonal symmetry.^110–113^ Weitzer *et al.* postulated that the *ϕ* phase may have decagonal pseudosymmetry.^103^ The Al_13_TM_4_ crystal structure tolerates a wide composition range encompassing various combinations of Co, Cr, Fe, Ni substitutions on the transition metal (TM) site.^108,115^ Ostrowska *et al.* performed thermodynamic modeling of Al–Co–Cr–Fe–Ni high entropy alloys.^108^ One of the phases they included in their modeling was Al_13_TM_4_ expressed as (Ni,Cr,Fe)_0.235_(Al)_0.6275_(Al,Va)_0.1375_, where Va denotes a vacancy.^108^

Fig. 19 shows the XRD powder spectrum for the Cr, Fe doubly-promoted Purdue alloy. Small new peaks appear at 21°and 39°, which likely correspond to the ‘Cr, Fe promoter phase’.

**Fig. 19:**
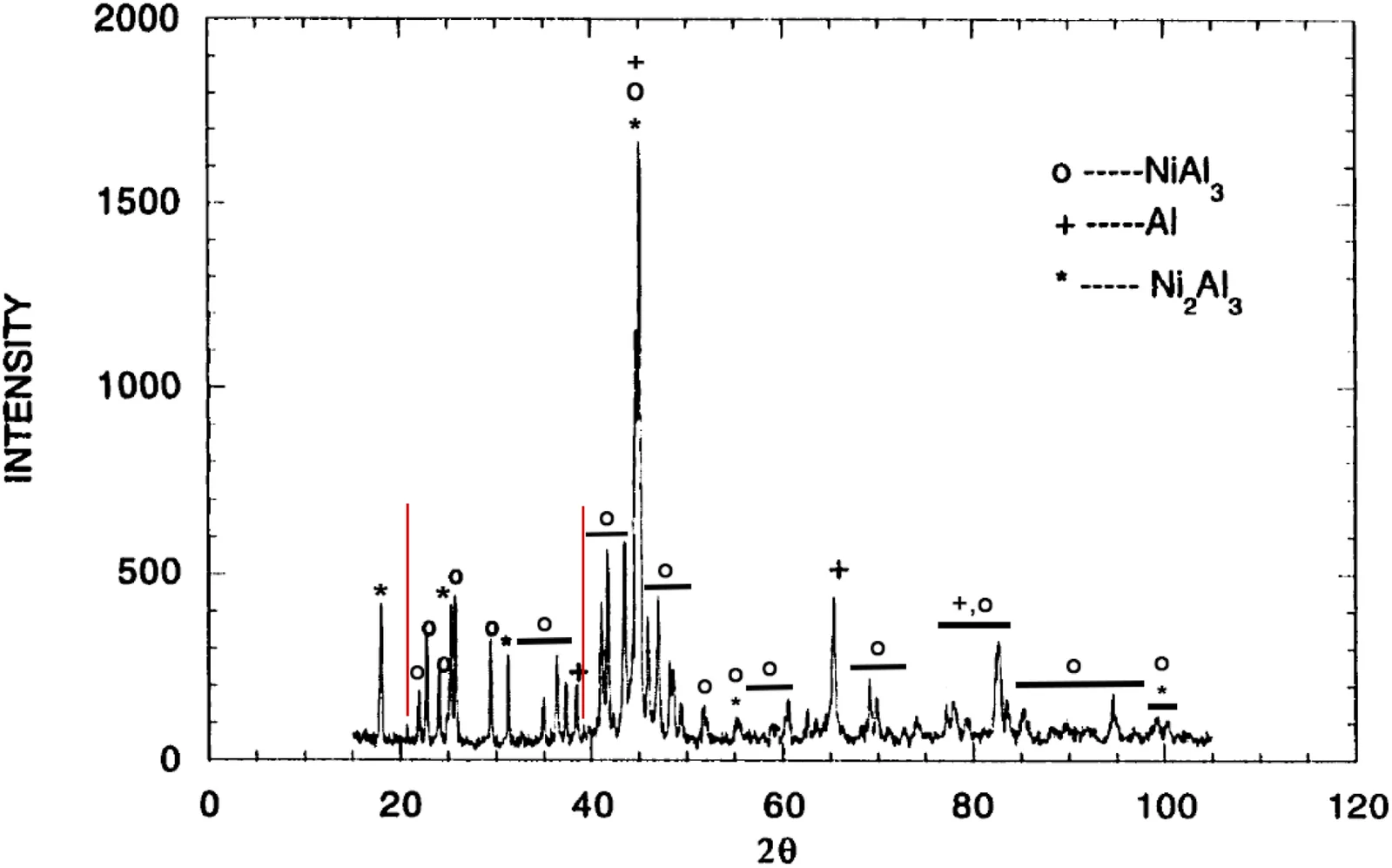
X-ray powder diffraction pattern of our 55 wt% Al, 42 wt% Ni, 1.5 wt% Fe, 1.5 wt% Cr alloy. The peak positions corresponding to various phases are labeled. Small peaks marked in red near 21° and 39° likely correspond to the ‘Cr, Fe promoter phase’.

### 3.4 Comparison to promoter-rich phases previously observed in Fe and/or Cr promoted RANEY® nickel precursor alloys

Using electron microprobe analysis, Kordulis *et al.*^136^ observed a Cr-rich phase in Cr-promoted RANEY® alloys; however, they did not assign any structure to this phase. The elemental composition they measured for this phase was approximately 67 at% Al, 25 at% Cr, and 8 at% Ni, which is not similar to the Cr-rich phase in our study.

Hamar-Thibault *et al.*^36^ observed a Cr-rich phase in Cr-promoted RANEY® alloys. However, they did not assign any structure to this phase. The elemental composition they measured for this phase is approximately similar to that reported in our work.

Hussain *et al.*^139,140^ studied 75 at% Al, 23.5 at% Ni, 1.5 at% Cr alloys made *via* gas-phase ionization. They identified a Al_13_Cr_2_ phase present in their alloys. This is a completely different phase than was present in our samples. Their phase had a different composition and was formed at lower temperatures than the Cr-rich promoter phase in our materials. Specifically, they stated “Elemental mapping and backscattered electron micrographs indicates that Al_13_Cr_2_ phase precipitates late in solidification, probably direct from the melt, during the final stages of Al_3_Ni growth.”^139^ The Al_13_Cr_2_ phase had nickel atoms substituted on some of the Cr sites, and this phase was located between the Al_3_Ni and eutectic phases.

Gros *et al.*^63^ studied (NiM)_2_Al_3_ alloys (M = Cr, Cu, Fe, Ti, Zr, Ta) prepared using three different techniques: (a) casting, (b) annealing for several days at high temperature, and (c) melt-spinning (which produces microcrystalline alloys). They observed a Fe promoter rich phase, which they tentatively attributed to (NiFe)Al_9_. They observed a Cr promoter rich phase, which had a composition of ∼26.1 at% Cr, ∼7.8 at% Ni, ∼66.1 at% Al, which they tentatively attributed to Al_9_(CrNi)_4_.

Warlimont *et al.*^99^ studied rapidly solidified ribbons (20–40 µm thick by 10 mm wide) of Al–Ni–Cr and Al–Ni–Fe alloys produced by the single roller method. In the Al–Ni–Fe alloys, they observed a Al_9_(Ni_*x*_Fe_2−*x*_) phase. Their alloys contained much higher proportion of Cr (*i.e.*, 7–17 at%) than the alloys studied in our work. Accordingly, they observed different Cr promoter rich phases than were observed in our work.

Maksimova *et al.*^32^ observed a FeNiAl_9_ phase present in Fe plus Mo doped RANEY® nickel alloys. They also observed a quaternary Al–Ni–Fe–Mo phase of unknown structure.

## 4. The leaching process and bound hydrogen

Before leaching, the alloys were crushed using a mortar and pestle and sieved to −100 mesh, which corresponds to particle diameters smaller than 150 µm.

Leaching the alloy in aqueous alkaline solution is an exothermic reaction that evolves hydrogen gas. Temperature control is important, especially during the early stages of the procedure when the alloy and alkali are combined. While leaching temperatures ranging from 0 °C to more than 100 °C have been reported in the literature, leaching temperatures of 50–95 °C have been reported most frequently.^17,18^

Using more than the stoichiometric amount (*i.e.*, an excess) of alkali is desirable^10^ to prevent the sodium aluminate from dissociating to form aluminum hydroxide which then gradually converts to a hydrated alumina:

2 Na^+1^ + OH^−1^ + Al(OH)_3_ ↔ Na^+1^ + [Al(OH)_4_]^−1^

3 2Al(OH)_3_ → Al_2_O_3_·3H_2_O

If alumina is allowed to precipitate, it will collect on the nickel surface and block the catalyst's pores.^116^ Most (but not necessarily all) of the precipitated alumina may be dissolved with additional fresh alkali to reactivate the catalyst.^116^

Studies have shown that optimum activity results when the vast majority of the aluminum is extracted during leaching.^117^ However, some aluminum should remain in the catalyst, because this residual aluminum stabilizes the catalyst by inhibiting the enlargement of nickel crystallites and also serves as a continuing source for hydrogen generation during storage to inhibit poisoning by oxygen.^118,119^ Sane *et al.* prepared sponge nickel catalysts from well-defined pure phases and found that the Ni_2_Al_3_ phase retains more residual aluminum after leaching than the leached NiAl_3_ phase does.^120^ Magnetic measurements by Macnab and Anderson^14^ have been interpreted to show that the residual aluminum is present interstitially in the crystal lattice. A combined experimental and Monte Carlo simulation study showed surface segregation of interstitially bonded residual aluminum.^56^

Freel *et al.*^9^ also studied the leaching process by scanning electron microscopy and concluded that the eutectic portion of the alloy is first to dissolve, followed by the NiAl_3_ phase, and finally the Ni_2_Al_3_ phase. Several authors^9,121,122^ found the activated catalyst microstructure retains some of crystallite sizes or orientations of the original alloy when examined at low magnification implying the leaching mechanism is selective dissolution, while at high magnification (1100×) the surface contained many cracks and fissures^9,16,123^ caused by the removal of aluminum.

We leached the promoted and unpromoted nickel–aluminum alloys using the following procedure. The alloy was crushed into a powder and sieved to −100 mesh. In a fume hood, the alloy powder was slowly added to an aqueous solution of 30 wt% NaOH in an oversized beaker. A 70% excess of NaOH solution was used during this procedure. In a fume hood, the beaker containing this alloy-alkali slurry was placed on a hot plate with stirring (using a magnetic stir bar) at 90 °C for 1 hour. After 1 hour of digestion, the sodium aluminate solution was decanted. The catalyst was then thoroughly washed twice in deionized water. Finally, the catalyst was washed with dilute NaOH solution and stored in a vial of 3 wt% aqueous NaOH.

The catalyst retains in its structure some of the hydrogen produced during leaching.^48^ This bound hydrogen contributes to the catalyst's activity and stability. Several investigators have proposed that there are two types of hydrogen in the catalyst: one strongly bound and one weakly bound. Temperature programmed desorption (TPD) studies carried out by Nicolau and Anderson^124^ showed the presence of two peaks in their thermogram, which they concluded was evidence for two types of hydrogen.

We performed temperature programmed desorption (TPD) of hydrogen for the unpromoted RANEY® nickel catalyst. Starting at room temperature (20 °C), hydrogen gas was flowed over the catalyst for five minutes while it was heated to 50 °C. Hydrogen gas was flowed over the catalyst for three hours while it was held at 50 °C. Helium gas was then flowed over the catalyst for 15 minutes at 50 °C. The main part of the TPD experiment was then performed as a 10 °C min^−1^ temperature ramp from 50 °C to 650 °C under a constant flow of helium gas at 35 cm^3^ STP per min. During this process, the desorbed hydrogen was measured as a function of temperature from 50 °C to 650 °C. As shown in SI Section S7, the main peaks were observed at 145 °C and 250 °C. This confirms there are two types of hydrogen present on the catalyst: weakly bound (which desorbs around 145 °C) and strongly bound (which desorbs around 250 °C).

## 5. Bulk composition elemental analysis

Atomic absorption spectroscopy was used to measure the elemental compositions of four of the activated catalysts. Measurements were performed on a Perkins-Elmer 3110 Atomic Absorption Spectrometer equipped with a flame ionizer. Hollow cathode lamps from Fisher Scientific were used for each chemical element and operated at 80% of their maximum capacity.

Approximately 0.2 grams of catalyst were used. The catalyst was first rinsed in distilled and deionized (DDI) water. The catalyst samples were then dissolved in an aqueous solution containing nitric and hydrochloric acids. The samples were then diluted using DDI water to the appropriate concentration range for analysis.

During the measurement process, spectral interferences may occur from the presence of either molecular combustion products that exhibit broad-based absorption or particulate products that scatter radiation. Chemical interference is the second type of interference. The method of standard additions is a technique that can make it possible to work in the presence of a matrix interference without eliminating the interference itself. This technique involves adding aliquots of a standard to the sample, thereby allowing any interferent present in the sample to also affect the standard similarly.

The method of standard additions was used to obtain the results discussed here. Please see Pryor's PhD dissertation for additional procedural details related to sample preparation, the preparation and use of standard solutions, and calibration curves.^125^

Table 4 lists the bulk compositions for four of the activated catalysts measured using atomic absorption spectroscopy. The ‘precursor alloy composition’ is the composition of the alloy prior to leaching. To produce the active catalyst, the alloy was crushed to a powder and leached in concentrated sodium hydroxide aqueous solution. During leaching, some of the Al is oxidized and dissolved while the Ni remains unoxidized. Consequently, the activated catalyst has a higher Ni content than Al content. These measurements provide clear confirmation that most but not all of the Al was removed during leaching. These results also indicate more Al was removed during leaching of the promoted catalysts than that for the unpromoted catalyst. The measurement uncertainty is estimated be approximately ±2.5 wt%. Taking into account this measurement uncertainty, the results are consistent with the leaching process removing most of the Al atoms while most of the Ni, Fe, and Cr atoms are retained in the activated catalyst.

**Table 4:** Bulk compositions of the activated catalysts as measured by atomic absorption spectroscopy. These were made from the Purdue alloys. The measurement uncertainty is estimated to be approximately ±2.5 wt%

	Precursor alloy composition	Activated catalyst composition (wt%)			
		Ni	Al	Fe	Cr
Unpromoted	Ni_42_Al_58_	83	17	—	—
High Cr	Ni_42_Cr_4.0_Al_54_	85	4	—	11
High Fe	Ni_42_Fe_4.0_Al_54_	82	7	11	—
Fe, Cr (doubly promoted)	Ni_42_Cr_1.5_Fe_1.5_Al_55_	83	8	4	5

## 6. Surface areas and pore size distributions

The amount of metallic nickel present at the surface is most often determined from carbon monoxide or hydrogen gas chemisorption.^18,126^ The literature reports values of the nickel surface area in the range from 30 to 90% of the BET total surface area.^18,126,127^ When compared to the bulk composition of the samples, these values could suggest that the surface usually contains less metallic nickel than the bulk. The pore structure of RANEY® nickel has also been previously studied by numerous authors.^19,39,47^

Table 5 lists the surface areas of the catalysts formed by leaching of the Purdue alloys. The total surface areas were computed using the Brunauer–Emmett–Teller (BET^128^) model applied to the nitrogen adsorption isotherm at 77 K. Ref 129 gives the detailed experimental procedure used to measure the nitrogen adsorption isotherm at 77 K. As shown in Table 5, the Fe and Cr promoted catalysts had somewhat larger total surface areas than the unpromoted catalyst. The Cr, Fe doubly-promoted catalyst had the highest total surface area. This trend is reproducible, because our previously published results for the Grace catalysts showed similar results.^127^

**Table 5:** Measured surface areas and pore properties for RANEY® catalysts formed by leaching the Purdue alloys

	BET SA (m^2^ g^−1^)	CO SA (m^2^ g^−1^)	H_2_ SA (m^2^ g^−1^)	Total pore volume (cm^3^ g^−1^)	Average pore diameter (nm)	Classification
Unpromoted	70	27.1	36.7	0.101	5.0	Mesoporous
Low Cr	116.4	46.9	40.0	0.073	1.5	Microporous
High Cr	87.5	47.5	40.2	0.034	1.0	Microporous
Low Fe	103	35.5	36	0.119	3.5	Mesoporous
High Fe	93	37.0	30.2	0.129	4.0	Mesoporous
Cr, Fe promoted	129.6	45.2	38.5	0.092	1.2	Microporous

For each catalyst, the average pore diameter was computed from the total pore volume (V) and total surface area (S.A.) assuming cylindrical pores. For cylindrical pores, the volume per pore is π*Ld*^2^/4 while the area per pore is π*Ld*, where *L* is the cylindrical pore's length and *d* is its diameter. Combining these two equations gives the following formula for the average pore diameter:

4 *d*_avg_ = 4*V*/(SA)

where the BET total surface area is used. The total pore volume was determined from the volume of adsorbed N_2_ at 77 K. Table 5 summarizes the results. The doubly-promoted and Cr-promoted catalysts had much smaller average pore diameters than the unpromoted and Fe-only promoted catalysts. Taking into account the XPS results (see Section 7), this effect is most likely due to the formation of a Cr(+III) compound that increases the catalyst's total surface area while reducing its average pore diameter. Based on the average pore diameter values, the doubly-promoted and Cr-promoted catalysts were classified as microporous (*i.e.*, average pore diameter < 2 nm), while the unpromoted and Fe-only promoted catalysts were classified as mesoporous (average pore diameter 2–50 nm).

For each catalyst, the distribution of pore volume *versus* pore diameter was computed using the Barrett, Joyner, and Halenda (BJH^130^) method. These plots are presented in SI Section S3. These plots showed unimodal distributions. Across the various catalysts, the peak position of the pore volume distribution followed the same trend as average pore diameter discussed in the prior paragraph.

Metallic surface areas were measured using carbon monoxide (CO) chemisorption and hydrogen (H_2_) chemisorption. These measurements were performed on the Micromeritics ASAP adsorption unit following the nitrogen adsorption experiment discussed above. Prior to data collection, the sample was degassed *in situ* at 120 °C for 8–12 hours. A CO adsorption isotherm was measured at 35 °C on the original evacuated sample. Then the sample was evacuated for an additional 30 minutes at 35 °C and a second CO isotherm determined. Each of these two isotherms were fit to a linear adsorption isotherm model, and the difference between the two gave the amount of chemisorbed CO, which was converted into surface area. The chemisorption of hydrogen was measured in a similar way by the determination of two isotherms at 35 °C. The experimentally-measured adsorption isotherms are provided in the SI Sections S4 (for CO) and S5 (for H_2_). Upon completion of the chemisorption measurements, the sample tube was removed from the unit and weighed. The final weight of dry catalyst was used for accurate calculations of the total and metallic surface areas.

As shown in Table 5, the metallic surface area was 30–54% of the total surface area. These results are also consistent with our previously published CO chemisorption results for the Grace catalysts.^127^ Examining Table 5, the metallic surface areas from CO and H_2_ chemisorption were approximately consistent with each other. The largest difference was for the unpromoted catalyst (27.1 m^2^ g^−1^ for CO *versus* 36.7 m^2^ g^−1^ for H_2_).

## 7. X-ray photoelectron spectroscopy (XPS)

Chemisorption is, in principle, sensitive only to the uppermost layer. On the other hand, the thickness of the surface layer analyzed by XPS amounts to about 5 to 10 atomic monolayers. We measured XPS for several of the catalysts.

XPS experiments were performed on a PerkinElmer PHI 5300 ESCA system with a Mg anode operated at 15 kV and 300 W. Catalysts were typically washed in distilled and deionized water five times, then loaded into the XPS sample holder. To protect the sample from air exposure, it was always covered in a layer of water. The wet sample was degassed overnight at room temperature in the XPS introduction chamber, prior to data collection. Ni and Al foils were used to determine binding energies corresponding to the metallic and oxidized states.

Plots of the XPS spectra peaks are contained in Fig. 20 (for Cr) and in SI Section S6 (for Al, Ni, and Fe). The Al_2p_, Ni_2p3/2_, Cr_2p3/2_, and Fe_2p3/2_ core-electron binding energies (eV) are listed in Table 6. The binding energy values indicate that Fe was mostly in the unoxidized metallic state with a trace of Fe(+III). As shown in Fig. 20, Cr was found only in the +III oxidation state. As explained in our prior publication, these XPS results showed that when the catalysts had been stored in 3 wt% sodium hydroxide aqueous solution, the Ni and Al are in the unoxidized metallic state.^127^ Earlier XPS results of Delannay *et al.*^131^ and Kordulis *et al.*^136^ also showed that Ni and Al are unoxidized when the catalyst is not exposed to air or other oxidants. The XPS results of Kordulis *et al.*^136^ showed that Cr is completely oxidized and accumulates on the catalyst's surface.

**Fig. 20:**
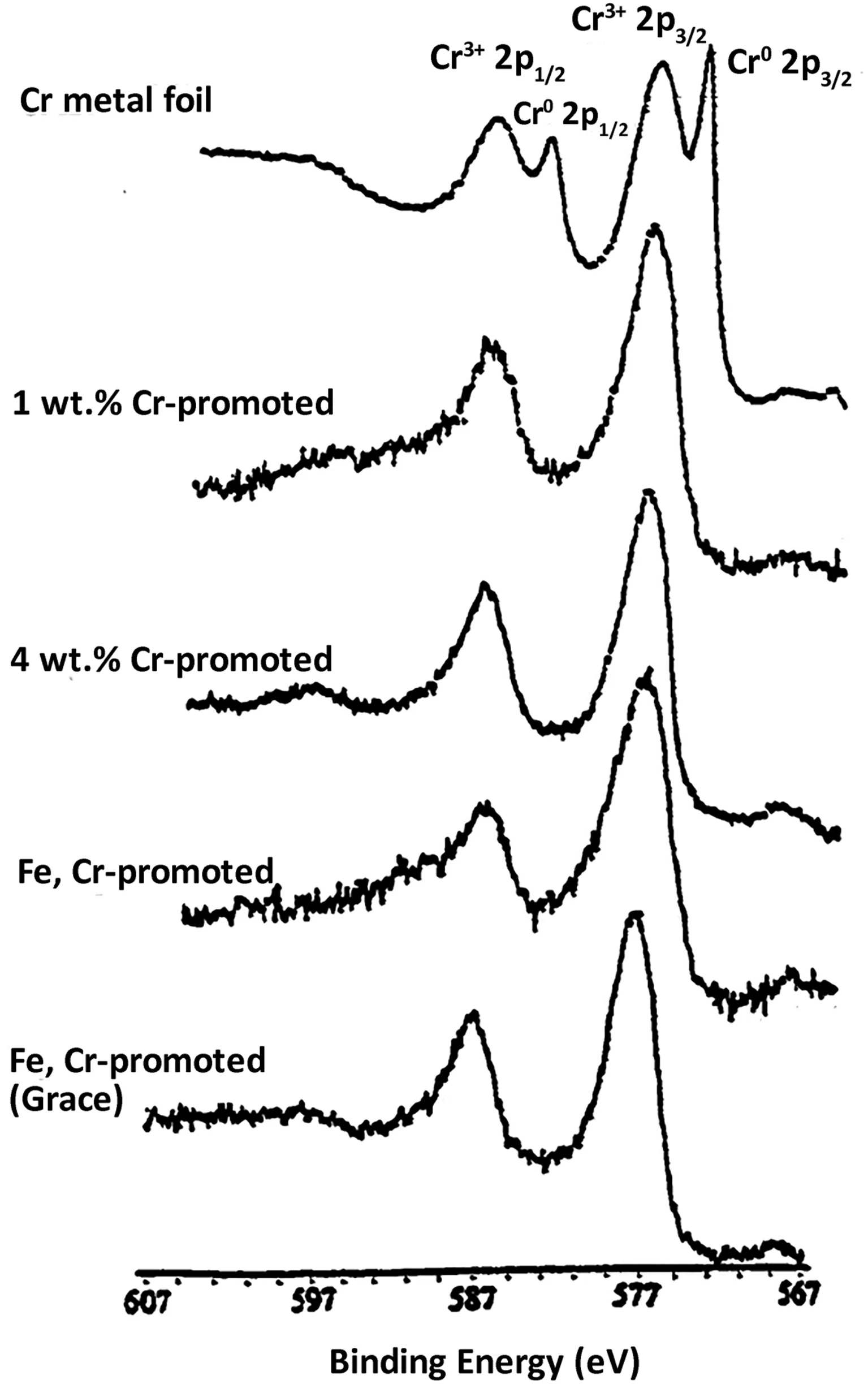
XPS spectra showing the chemical state of chromium for the active catalysts. These results show that Cr is found in the Cr(+III) oxidation state in the active catalysts.

**Table 6:** XPS core-electron binding energies (in eV) for different chemical elements in RANEY® catalysts. Except for the last row, each of these catalysts were prepared from the Purdue precursor alloys. The catalyst in the last row was prepared from the commercial-grade Grace Davison alloy

Catalyst	Al_2p_	Ni_2p3/2_	Cr_2p3/2_^a^	Fe_2p3/2_
Unpromoted	72.5	852.6	—	—
Low Cr	72.4	852.6	577	—
High Cr	72.4	852.6	577.3	—
Low Fe	72.5	852.6	—	707.2
Fe, Cr promoted	72.5	852.6	577.3	707.6/710.4
Fe, Cr promoted (GRACE)	72.7	852.7	577.5	—

a Cr_2p3/2_ peak to oxide species.

Using XPS cross-sections, the Al : Ni, Cr : Ni, and Fe : Ni element atomic ratios were computed. SI Table S1 compares these XPS-measured element ratios to the bulk composition ratios measured by atomic absorption (AA) spectroscopy. Among these results, the strongest and most consistent observation is the surfaces of the Cr and doubly-promoted catalysts were enriched in Cr compared to their bulk compositions. Since the binding energies indicate Cr is in the +III oxidation state, these results show that a Cr(+III) compound concentrated on the catalyst's surface. The results for Al and Fe were more complicated. As reported in the prior literature, Al segregates towards the surface.^132^

The mechanism for Cr enrichment on the catalyst's surface is as follows. During leaching, Al is oxidized by H_2_O in the presence of excess NaOH to form the soluble Na^+^[Al(OH)_4_]^−^ which is removed during catalyst washing. During leaching, Cr is oxidized by H_2_O in the presence of excess NaOH to form insoluble Cr(+III) oxide-hydroxide compounds (*e.g.*, Cr(OH)_3_ or CrO(OH)), which are insoluble.^36,71,74,75^ These precipitate on the catalyst's surface causing the observed Cr(+III) enrichment.^36,71,74,75^

## 8. Conclusions

An unpromoted RANEY® Ni–Al alloy prepared by arc melting had relatively small-sized phase grains (∼10 µm in diameter as shown in Fig. 2) due to the rapid cooling during alloy synthesis. When leached in aqueous sodium hydroxide solution, the eutectic phase was leached first, followed by the NiAl_3_ phase, and finally the Ni_2_Al_3_ phase was the slowest to leach. The NiAl_3_ phase produces small pieces during leaching. This agrees with previous publications.^121,123^

Using an induction furnace, several RANEY® Ni–Al alloys were synthesized that contained different levels of Fe and/or Cr promotion. These alloys were characterized using electron microprobe analysis, backscattered SEM, optical microscopy, and X-ray diffraction.

Four promoter phases were visible in the backscattered SEM images and electron microprobe results. ‘Fe promoter phase 1’ had a composition of approximately Al_71_Ni_24_Fe_5_ and was observed in some of the Fe-only promoted alloys that contained ≥2 wt% Fe. ‘Fe promoter phase 2’ had a composition of approximately Al_9_Ni_(2−*y*)_Fe_*y*_ (where *y* was 0.37 to 0.77) and was observed in all of the Fe-only promoted alloys. ‘Cr promoter phase’ had a composition of approximately Al_77.5_Cr_12_Ni_10.5_ and was observed in all of the Cr-only promoted alloys. ‘Cr, Fe promoter phase’ had a composition of approximately Al_75.5_Ni_13–14_Cr_6–7_Fe_4_.

For each promoter phase, we considered the following factors to propose a structural assignment: (a) the phase's elemental composition in our alloy samples, (b) previously published reports of a phase with similar elemental composition (whose detailed structure was assigned in the literature reports) formed under similar temperature and pressure conditions, and (c) peak positions observed in the XRD powder patterns of our precursor alloys as compared to those reported in the literature for the reference phases. Based on these considerations, we propose that ‘Fe promoter phase 1’ is the decagonite quasicrystal phase. We propose ‘Fe promoter phase 2’ is the Al_9_Ni_(2−*y*)_Fe_*y*_ (where *y* was 0.37 to 0.77) *τ*_2_ crystalline phase, which has been reported in several previous studies.^63,82,99^ We propose ‘Cr promoter phase’ (present in Cr-only promoted alloys) is the *ϕ* phase observed in previous studies of the Al–Ni–Cr phase diagram.^103,104^ The identity of the ‘Cr, Fe promoter phase’ (present in Cr, Fe doubly-promoted alloys) is currently unknown. We encourage additional research to further study these phase assignments.

Before leaching, the alloys were crushed using a mortar and pestle and sieved to −100 mesh. This corresponds to particle diameters smaller than 150 µm.

After leaching these alloys in concentrated sodium hydroxide aqueous solution, the Cr promoter was oxidized to Cr(+III). An insoluble Cr(+III) compound remained on the catalyst's surface. The XPS binding energy values indicated that Fe in the activated catalyst was mostly in the unoxidized metallic state with a trace of Fe(+III). During leaching, most of the Al was oxidized and removed as sodium aluminate, while nickel remained in the metallic unoxidized state. Some residual unoxidized aluminum remained in the active catalyst.

Some of the hydrogen produced during leaching was retained in the active catalyst. In agreement with prior literature,^124^ hydrogen TPD experiments confirmed that there are two types of hydrogen present on the unpromoted catalyst: weakly bound (which desorbs around 145 °C) and strongly bound (which desorbs around 250 °C).

Total surface areas were measured by BET analysis of N_2_ physisorption at 77 K. The Fe, Cr doubly-promoted catalyst had the largest total surface area (approximately 130 m^2^ g^−1^). Across the series of unpromoted and promoted RANEY® nickel catalysts, metallic surface areas were 27–47 m^2^ g^−1^ as measured by CO chemisorption and 30–40 m^2^ g^−1^ as measured by H_2_ adsorption. The Cr-promoted and doubly-promoted catalysts had the smallest average pore diameters and were classified as microporous. The unpromoted and Fe-only promoted catalysts were classified as mesoporous.

The Purdue alloys contained 42 wt% Ni, while the commercial-grade Grace alloys contained approximately 48–50 wt% Ni. In spite of this difference, the set of phases present and the phase compositions were similar for the Purdue alloys compared to the Grace alloys. Also, the BET total surface areas and metallic surface areas that we report here in Table 5 for catalysts prepared from the Purdue alloys are similar to values we previously reported^127^ for catalysts prepared from the Grace alloys.

The catalytic performance of these promoted RANEY® nickel materials was investigated by us in a companion article.^133^ In that study, butyronitrile hydrogenation in methanol solvent was performed at 1–15 atm hydrogen pressure. Fe and Cr promotion substantially improved the overall catalyst performance.^133^

We encourage additional research to study the following. How does the microstructure of the precursor alloys before leaching influence the microstructures and performance of the after-leached catalysts? Is there a relationship between the promoter-rich phases in the precursor alloys before leaching and the catalyst performance after leaching? To help answer these questions, the phase structures of the precursor alloys and leached catalysts should be studied in more detail. Electron probe microanalysis (EPMA) with wavelength dispersive spectroscopy (WDS) mapping would be a useful technique to map out the concentration distributions of various chemical elements throughout an alloy or catalyst sample. Micro-diffraction using a narrowly focused electron or X-ray diffraction beam would be a valuable technique to determine the structural assignments for individual phase grains. Follow-up work to perform micro-diffraction and structure refinement for the promoter-rich phases would be especially welcomed.

## Author contributions

The project was led and supervised by W. N. D. as the major professor. The alloys were synthesized by and optical microscopy was performed by S. T. P., K. P. T., D. R. G., and T. A. M. in Purdue's School of Materials Engineering. Professor K. P. T. supervised the alloy synthesis and optical microscopy. Electron microprobe analysis was performed by C. H. and T. A. M. X-ray diffraction analysis was performed by S. T. P., D. R., and T. A. M. XPS, leaching, atomic absorption spectroscopy, and gas adsorption experiments were performed by S. T. P., Z. L., and T. A. M. in W. N. D.’s laboratory in Purdue's School of Chemical Engineering. T. A. M. analyzed the promoter phase compositions, structures, and assignments. T. A. K. and S. K. S. were the project's industrial liaisons and sponsors. T. A. M. and S. T. P. wrote the manuscript with input from various co-authors.

## Conflicts of interest

There are no conflicts of interest to declare.

## Supplementary Material

RA-OLF-D6RA05868J-s001

RA-OLF-D6RA05868J-s002

## Data Availability

The data that supports the findings of this study are available within the article and its supplementary information (SI). Supplementary information: a PDF file containing: (1) X-ray diffraction spectra of alloys, (2) optical micrographs of the Purdue alloys, (3) N_2_ physisorption at 77 K to determine total surface areas and pore volume distributions, (4) carbon monoxide chemisorption isotherms, (5) hydrogen adsorption isotherms, (6) X-ray photoelectron spectroscopy (XPS) curves, (7) hydrogen temperature program desorption (TPD) curve, (8) safety considerations. A spreadsheet containing data workup for electron microprobe analysis. See DOI: https://doi.org/10.1039/d6ra05868j.
