# Supplementary material for: The microstructures of promoted RANEY® nickel alloys and catalysts
Source: RSC Adv. 2026 Nov 4. Online ahead of print. doi: 10.1039/d6ra05868j (PMC13733667; doi:10.1039/d6ra05868j)
Supplement: Supplementary file 1 [file RA-OLF-D6RA05868J-s002.pdf]

Supplementary Information (SI) for RSC Advances.
This journal is © The Royal Society of Chemistry

Supporting Information for

The Microstructures of Promoted Raney(r) Nickel Alloys and Catalysts

Thomas A. Manz*, Seqwana T. Pryor, Kevin P. Trumble, Zhufang Liu, David R. Gaskell, Dave Roberts, Carle Hager, Sourav K. Sengupta, Theodore A. Koch, and W. Nicholas Delgass
*corresponding author email: tmanz@nmsu.edu

Contents

X-ray diffraction spectra of alloys

Optical micrographs of the Purdue alloys

N_2 physisorption at 77 K to determine total surface areas and pore volume distributions

Carbon monoxide chemisorption isotherms

Hydrogen adsorption isotherms

X-ray photoelectron spectroscopy (XPS) curves

Hydrogen temperature program desorption (TPD) curve

Safety considerations

S1. X-ray diffraction spectra of alloys

Note: XRD spectra for the unpromoted, 4 wt.% Fe promoted, 4 wt.% Cr promoted, and doubly promoted alloys are presented in Section 3 of this article's main text. The XRD spectra for the 1 wt.% Fe promoted, 2 wt.% Fe promoted, 1 wt.% Cr promoted, and 2 wt.% Cr promoted alloys are shown below.

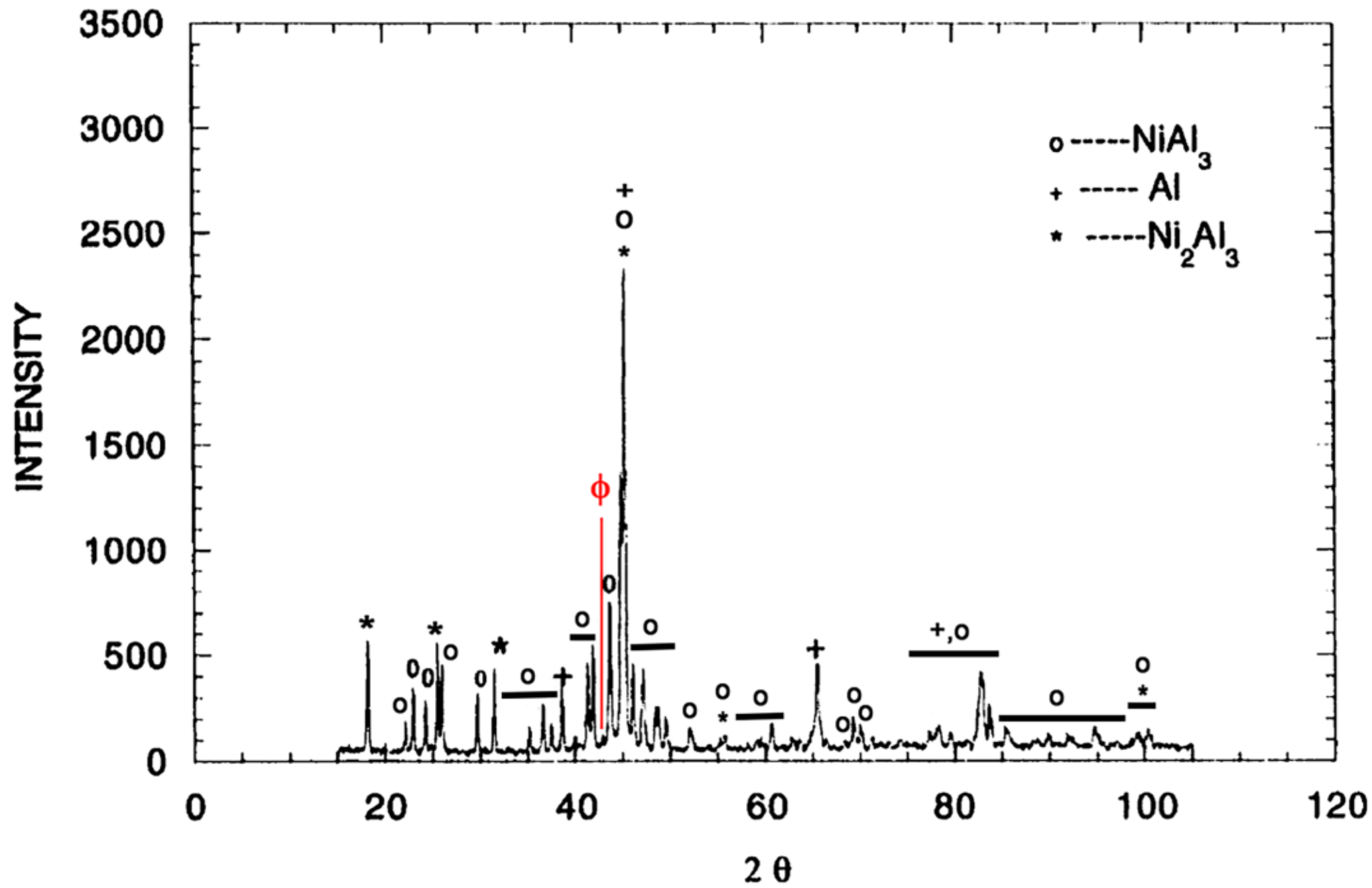

Figure S1: X-ray diffraction pattern of 1 wt.% Cr promoted Ni-Al alloy.

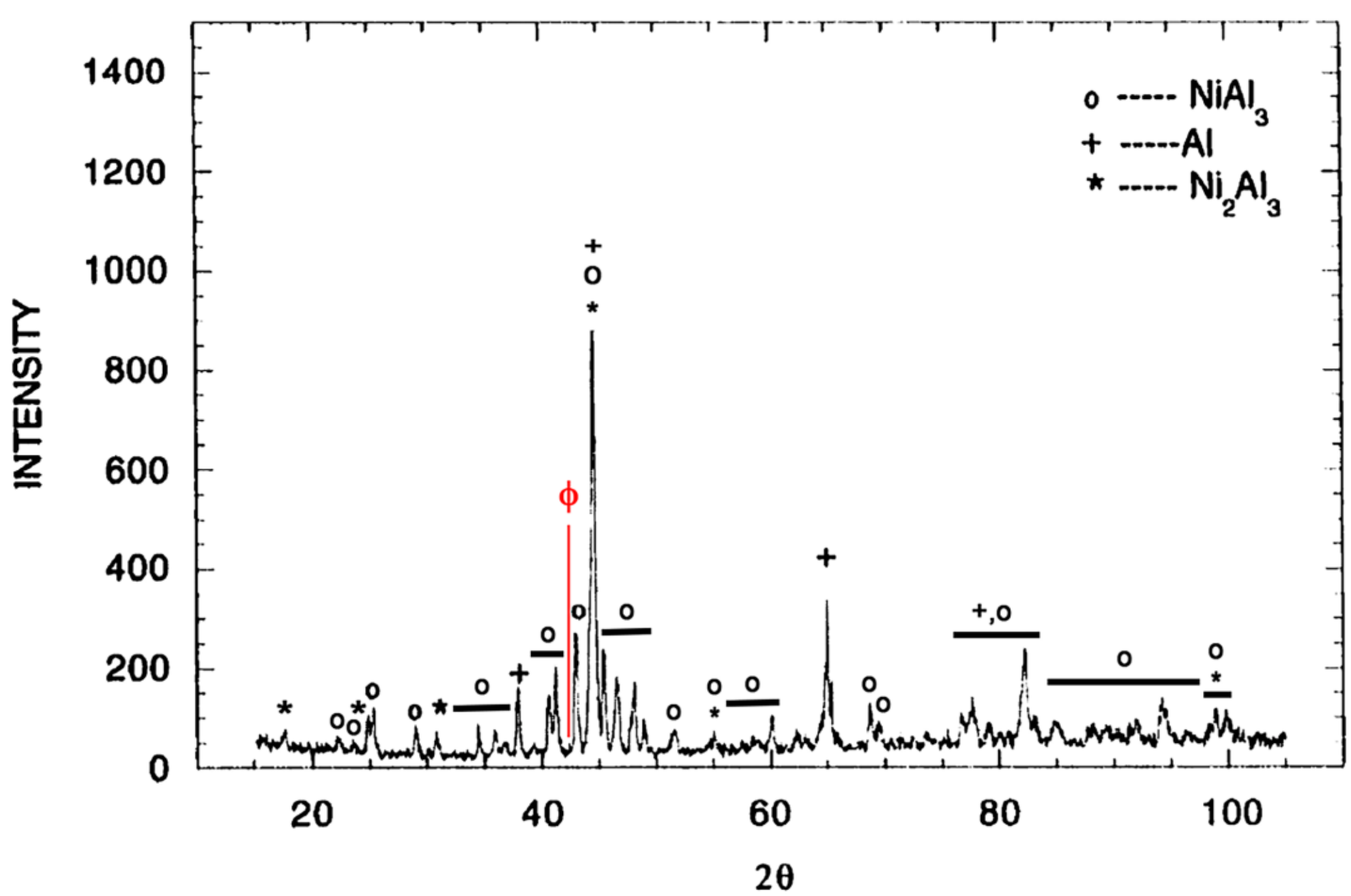

Figure S2: X-ray diffraction pattern of 2 wt.% Cr promoted Ni-Al alloy.

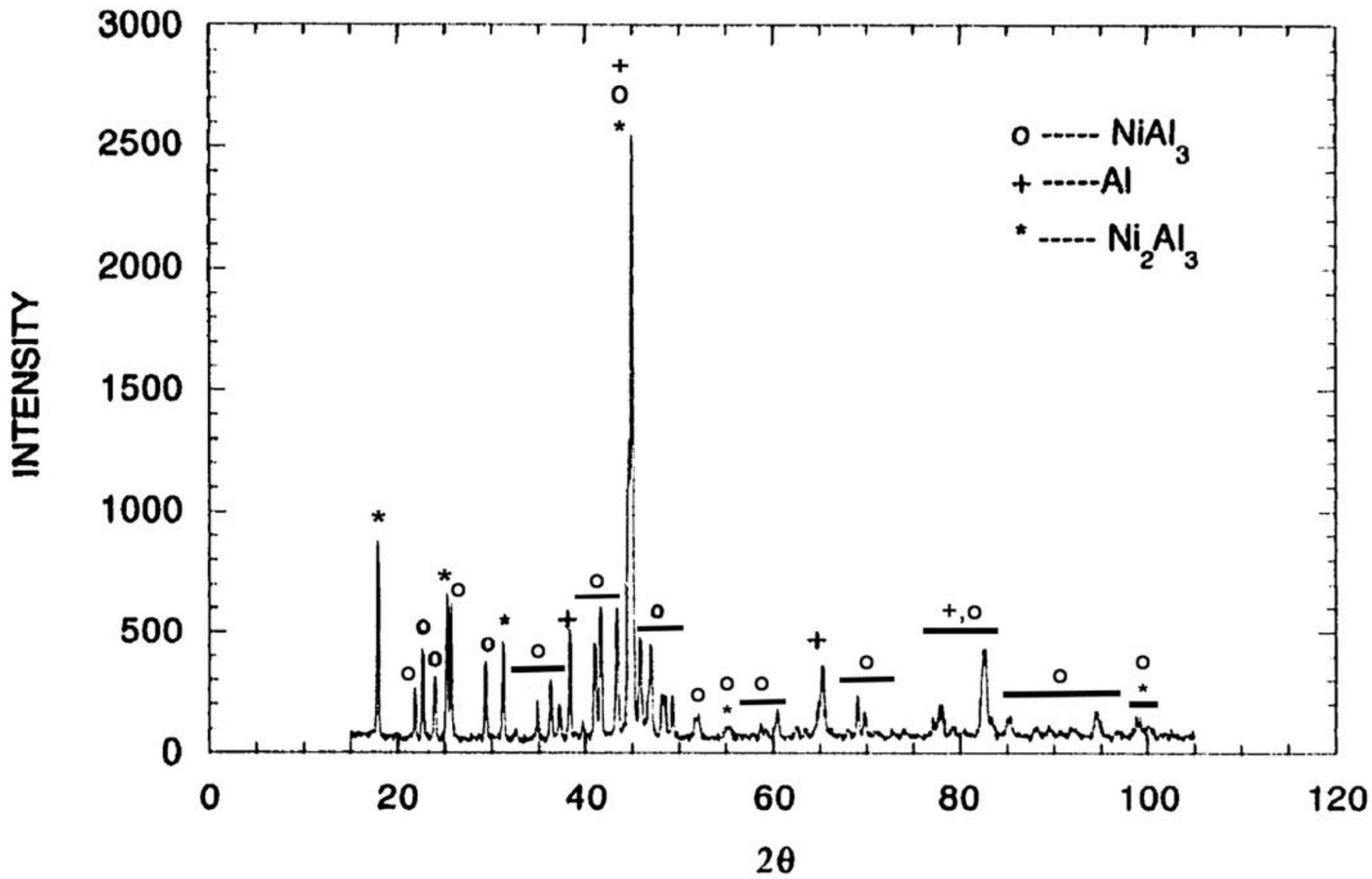

Figure S3: X-ray diffraction pattern of 1 wt.% Fe promoted Ni-Al alloy.

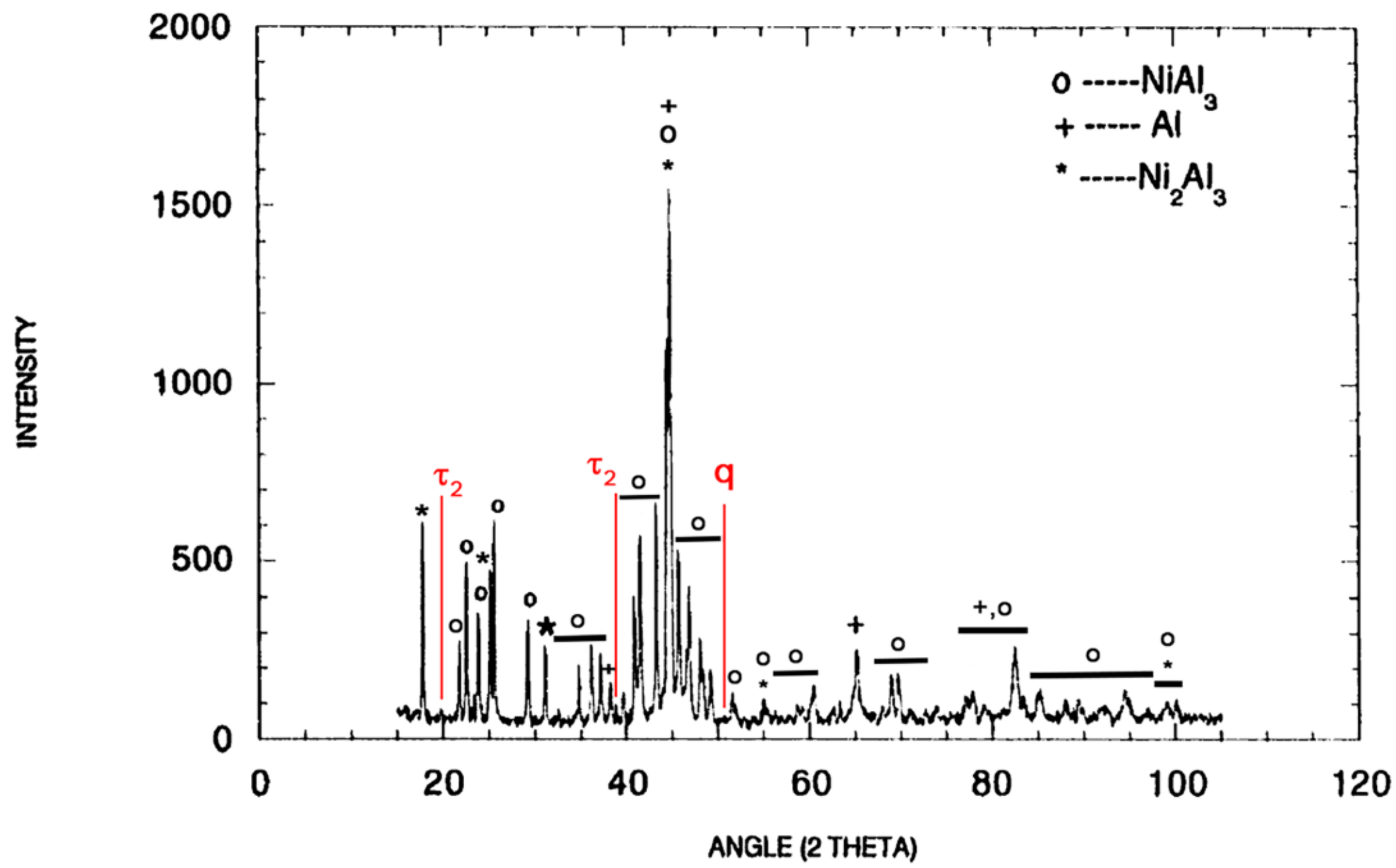

Figure S4: X-ray diffraction pattern of 2 wt.% Fe promoted Ni-Al alloy.

S2. Optical micrographs of the Purdue alloys

Note: The contrast and lighting in some of these optical micrographs is uneven, which can cause the same crystal phase to appear as a slightly different shade of grey in different parts of the image. In these images, the pure black regions are void spaces.

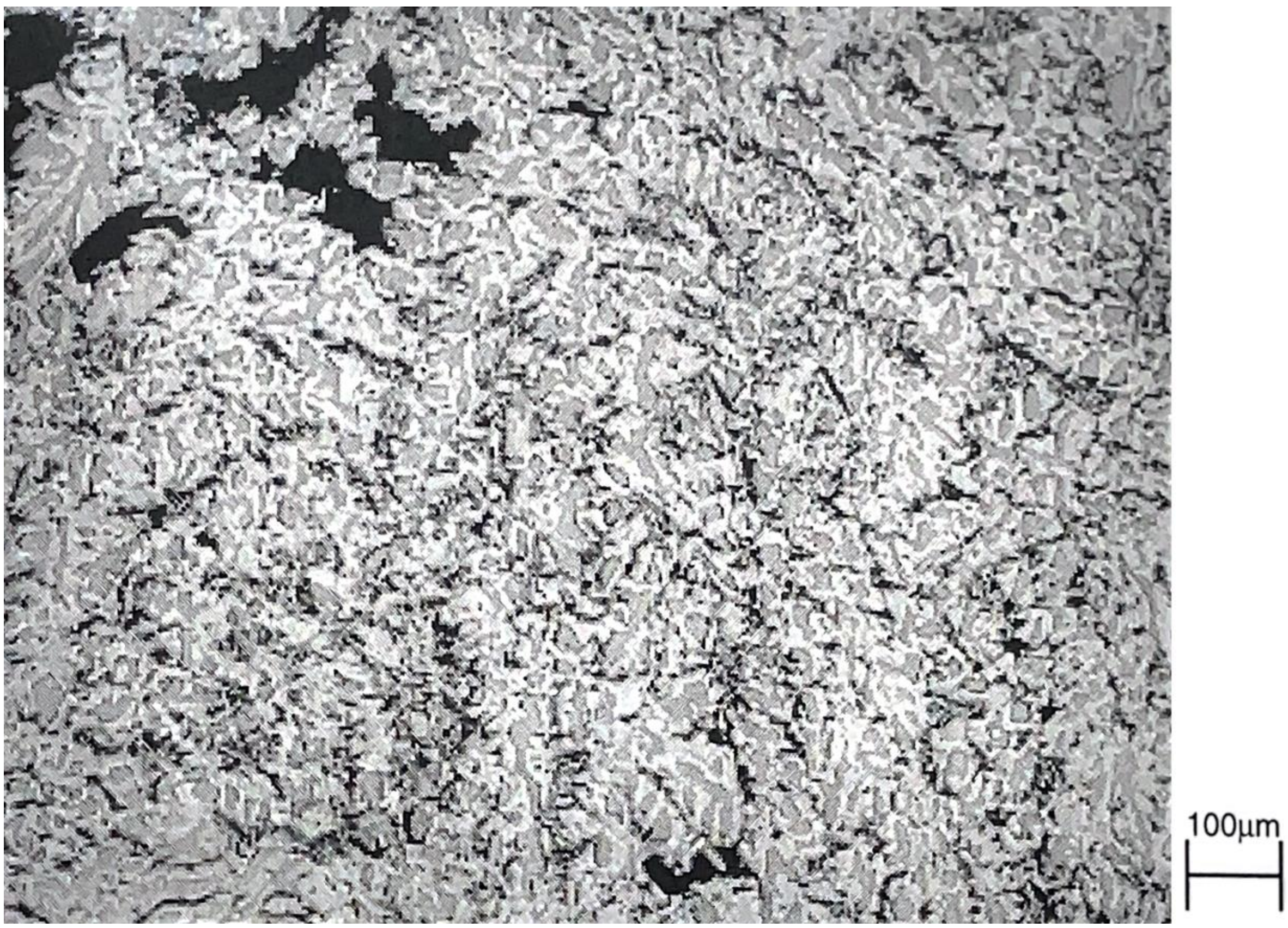

Figure S5: Optical micrograph of unpromoted Ni-Al alloy.

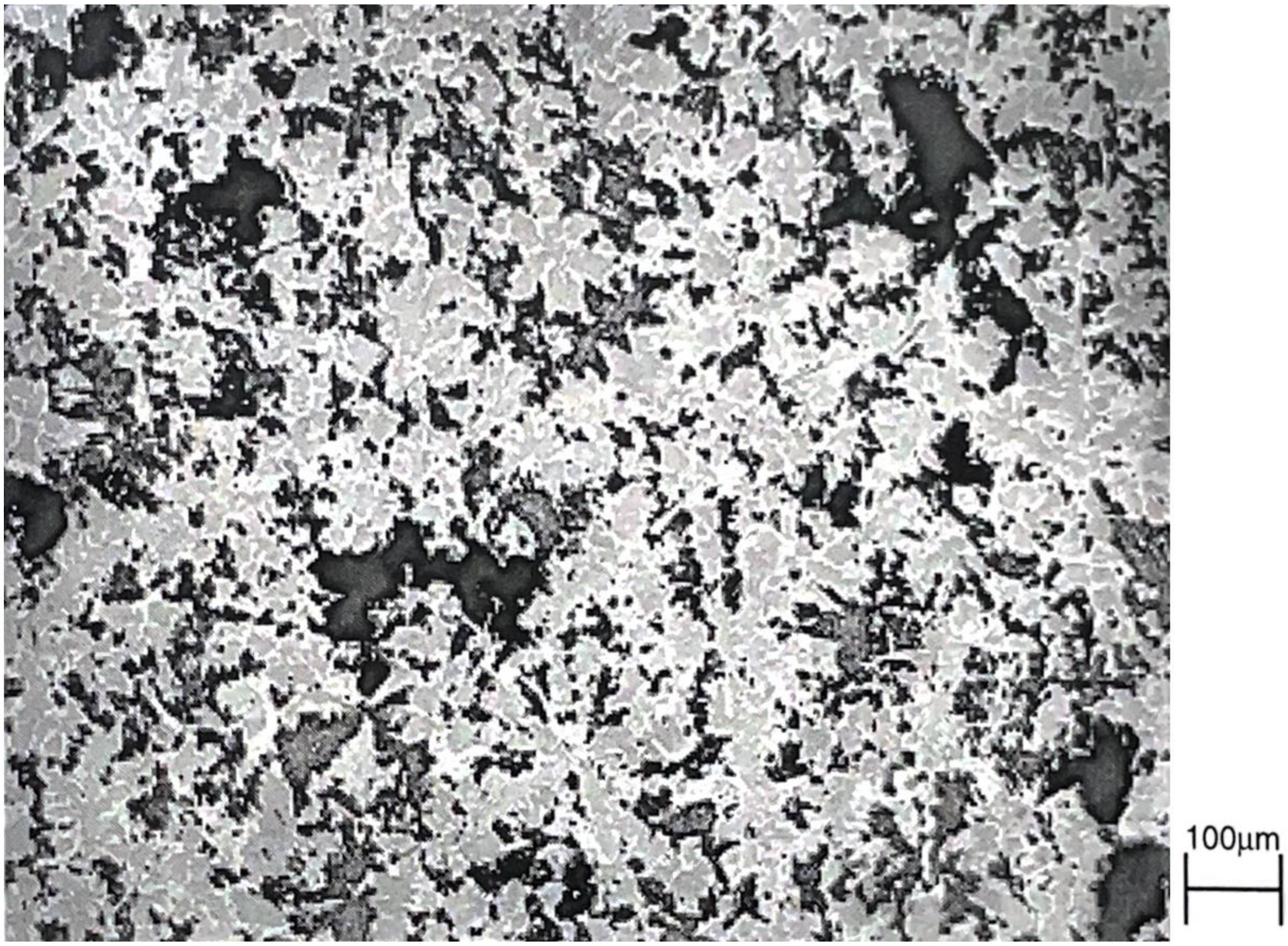

Figure S6: Optical micrograph of 1 wt.% Cr promoted Ni-Al alloy.

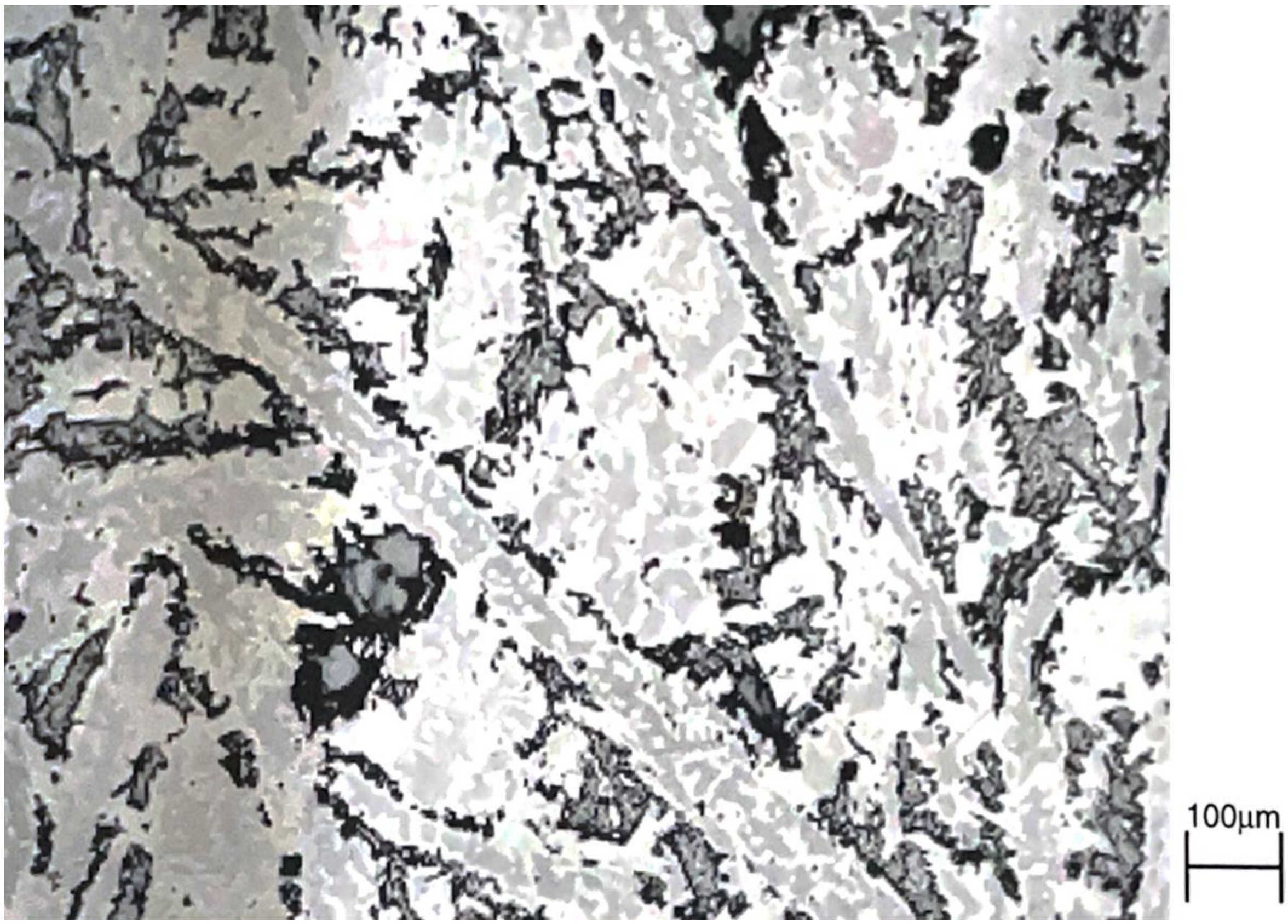

Figure S7: Optical micrograph of 2 wt.% Cr promoted Ni-Al alloy.

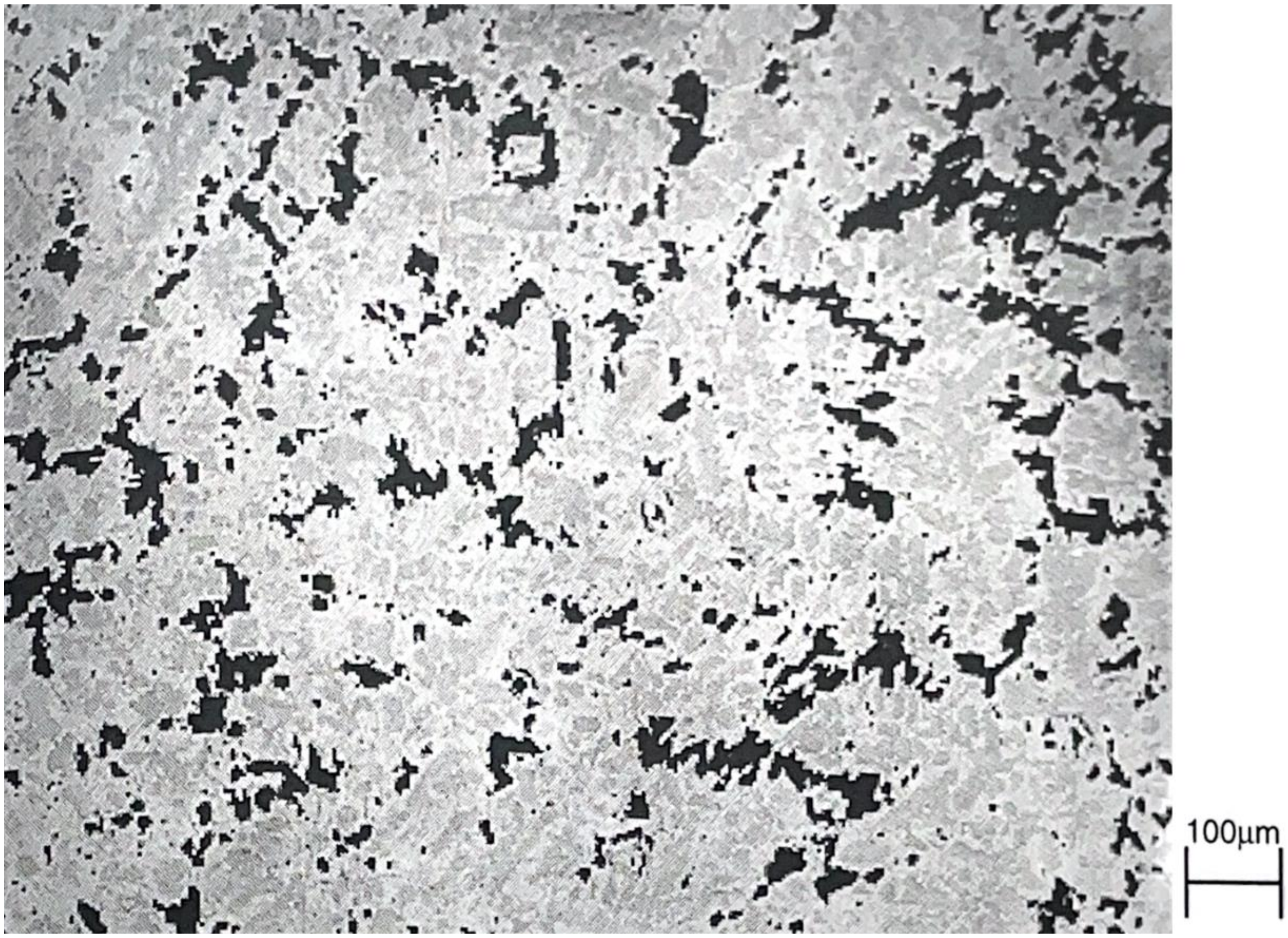

Figure S8: Optical micrograph of 4 wt.% Cr promoted Ni-Al alloy.

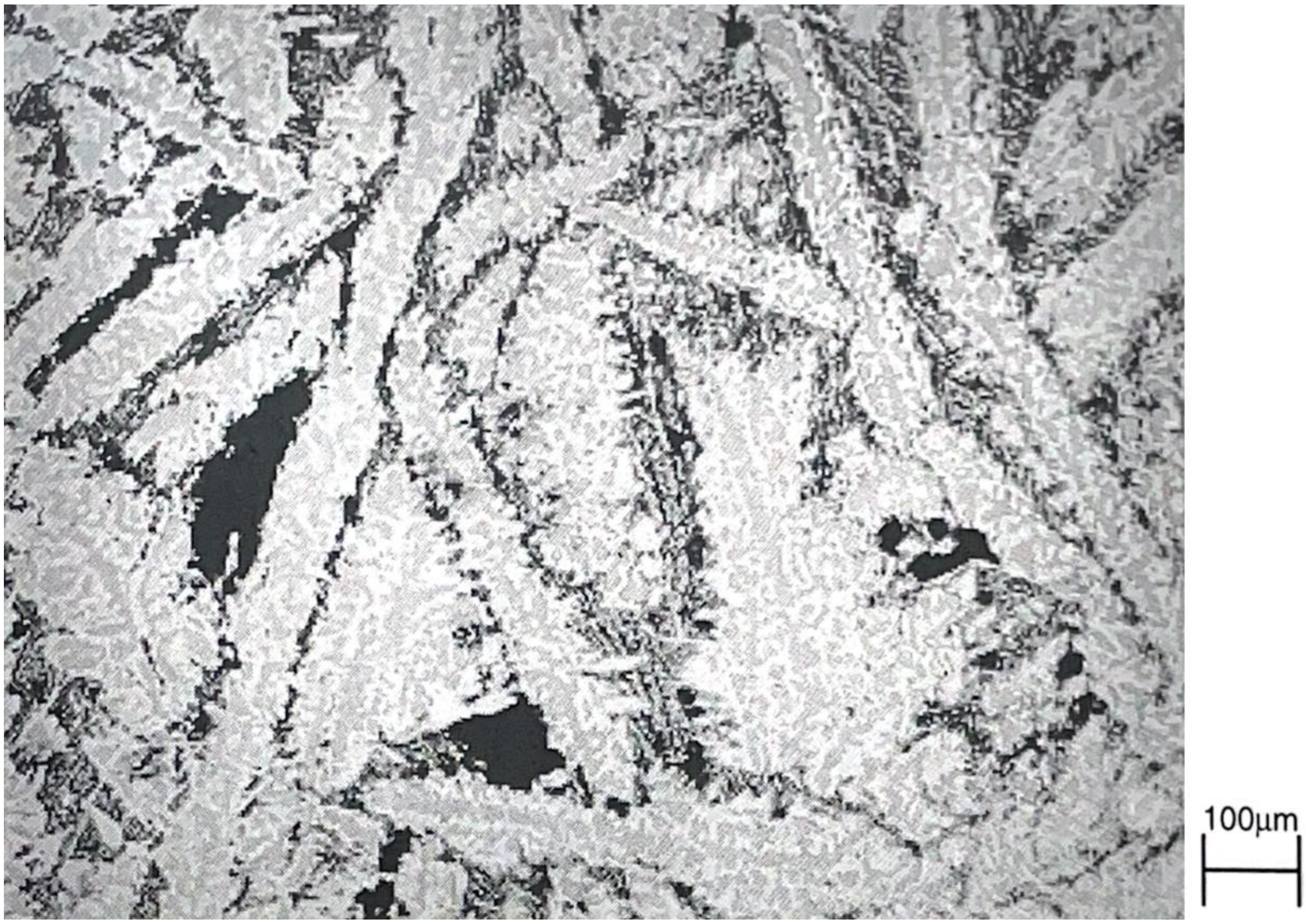

Figure S9: Optical micrograph of 1 wt.% Fe promoted Ni-Al alloy.

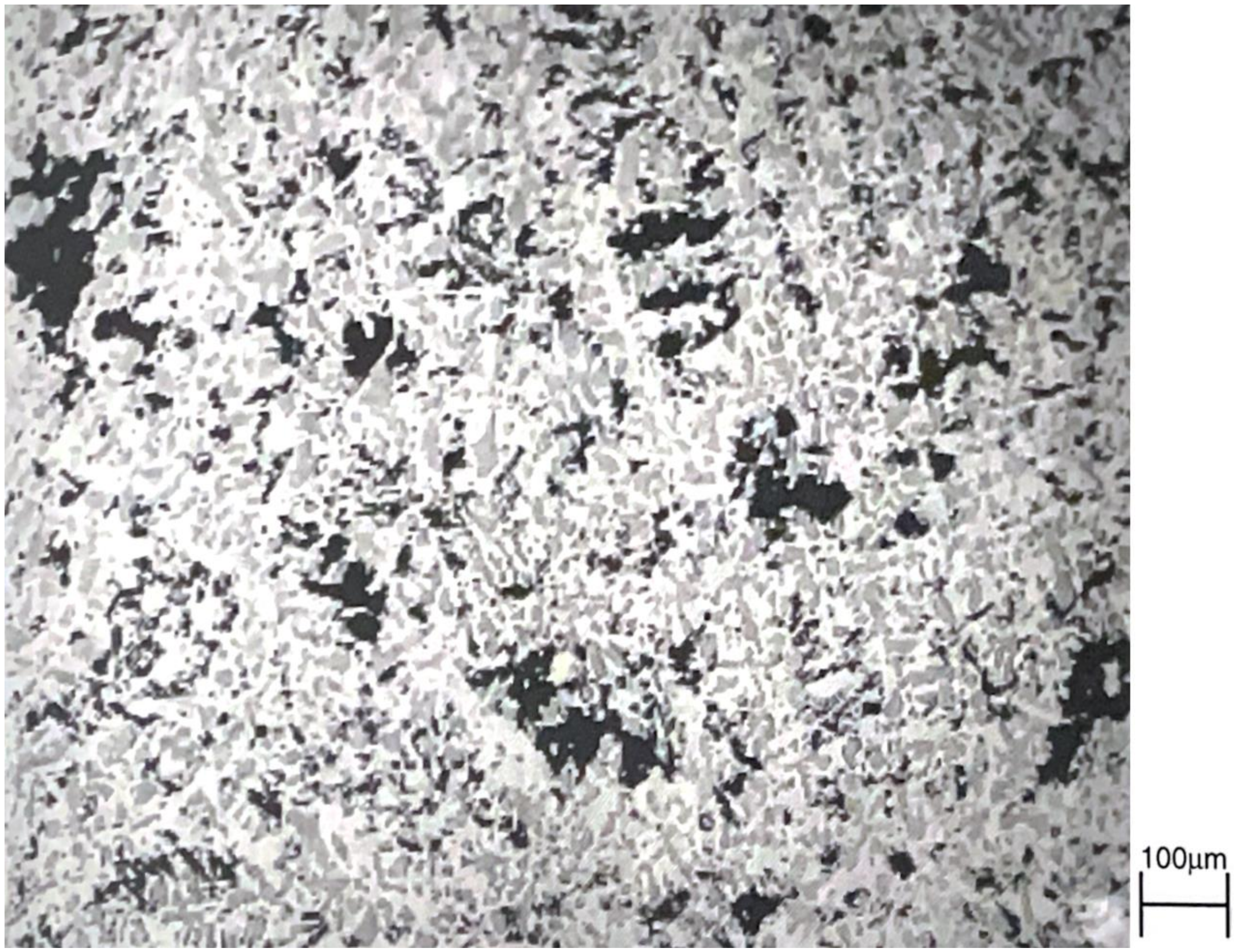

Figure S10: Optical micrograph of 2 wt.% Fe promoted Ni-Al alloy.

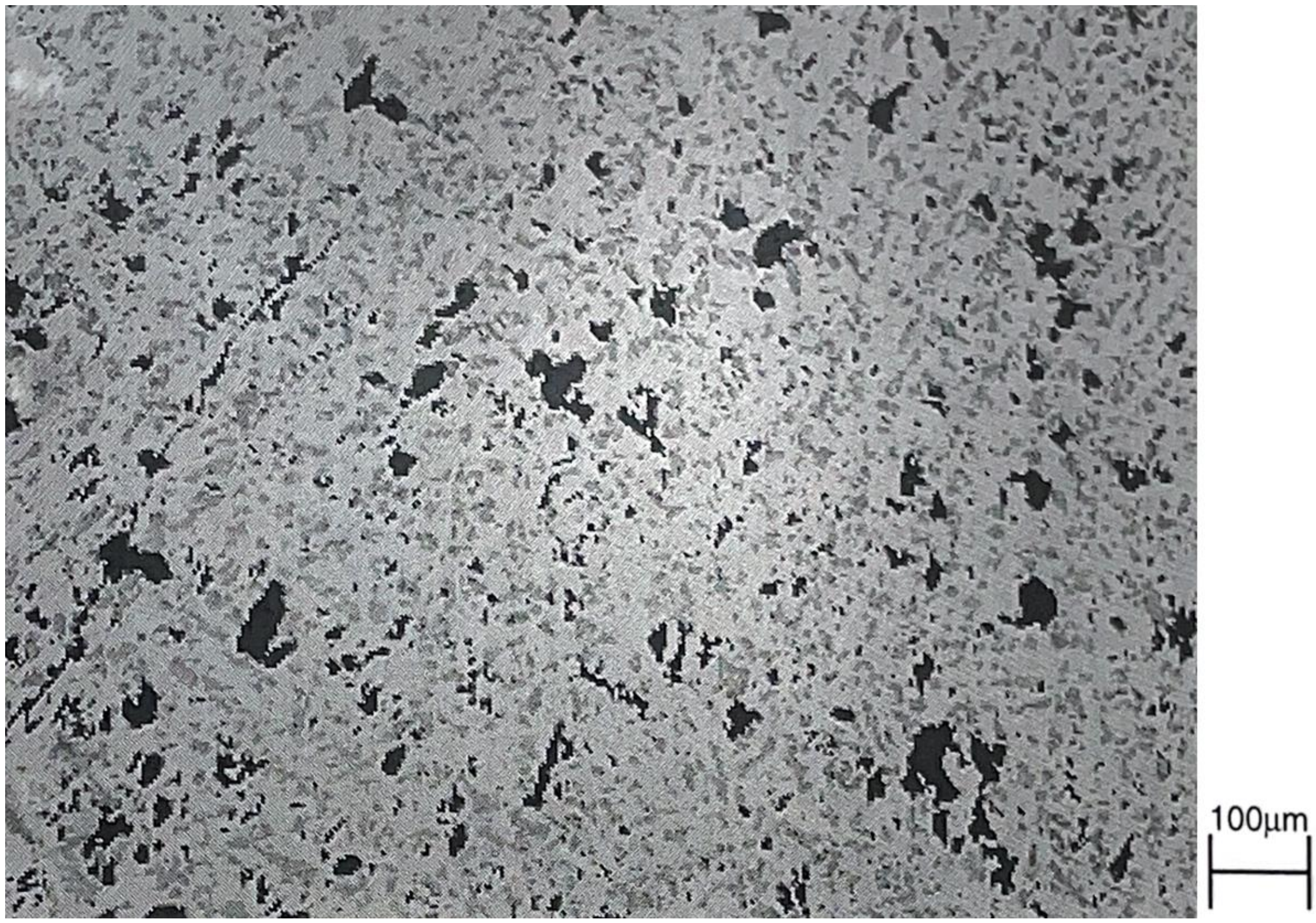

Figure S11: Optical micrograph of 4 wt.% Fe promoted Ni-Al alloy.

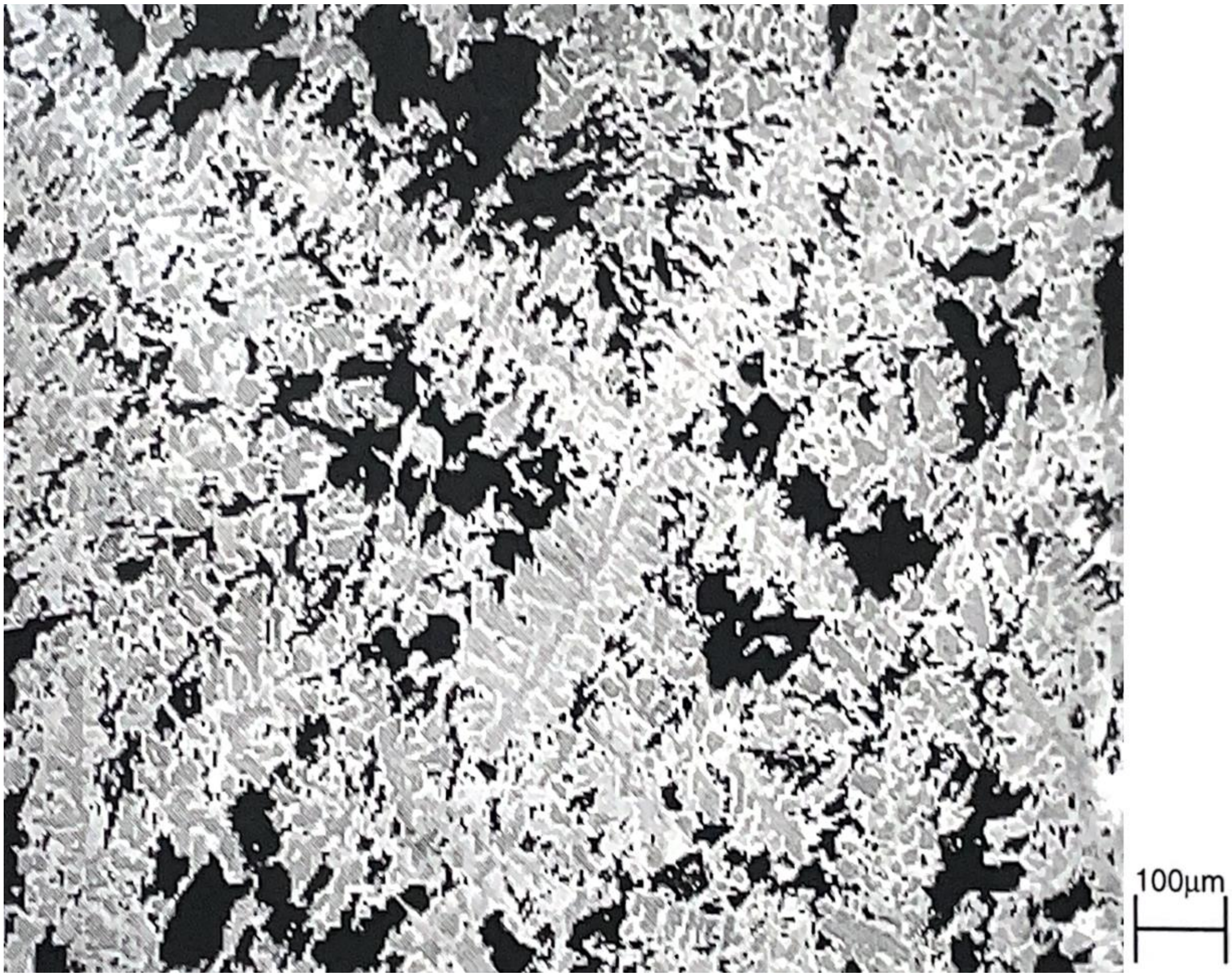

Figure S12: Optical micrograph of 1.5 wt.% Fe, 1.5 wt.% Cr promoted Ni-Al alloy.

S3. N_2 physisorption at 77 K to determine total surface areas and pore volume distributions

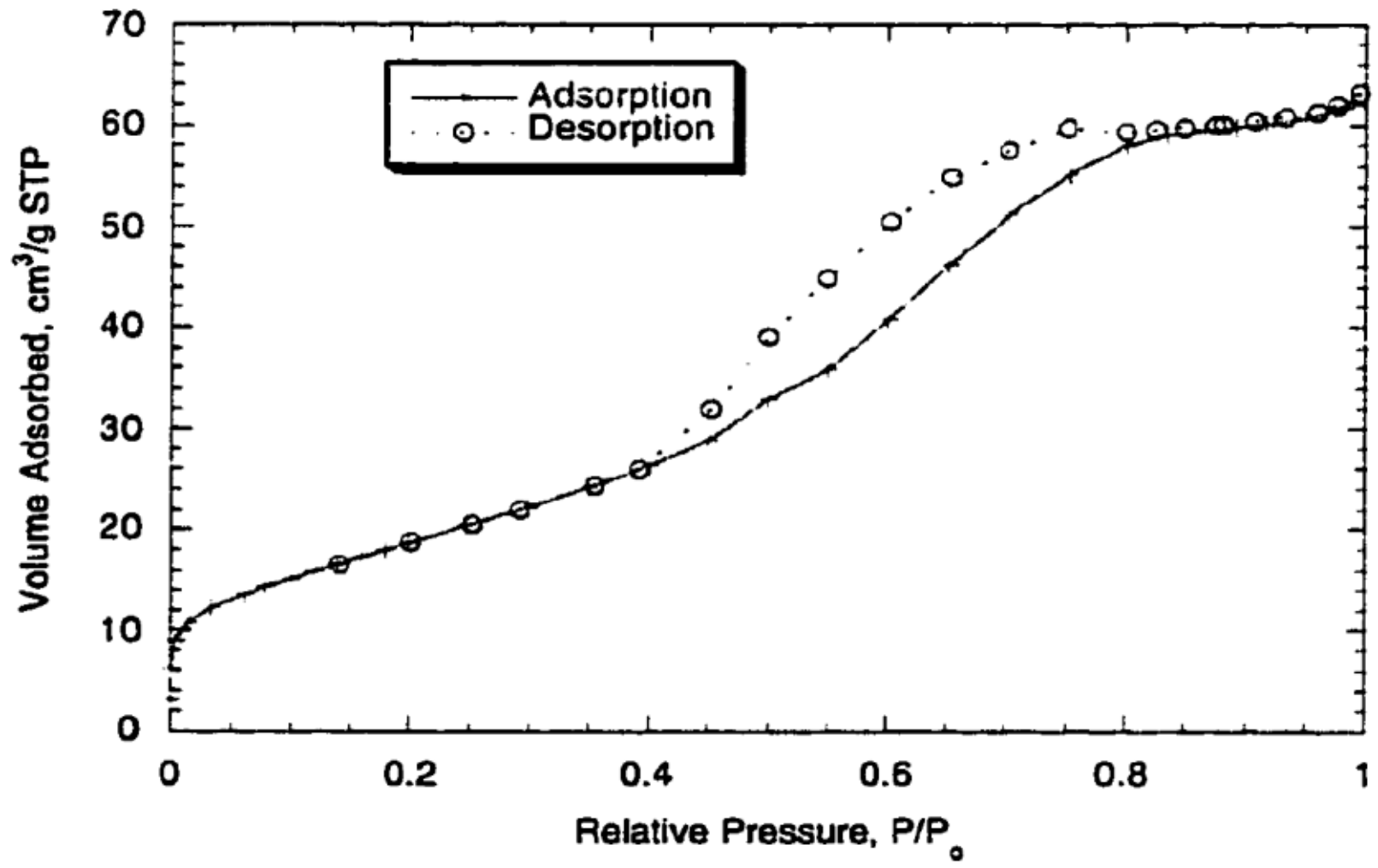

Figure S13: Unpromoted nickel catalyst N_2 adsorption and desorption isotherms at 77 K.

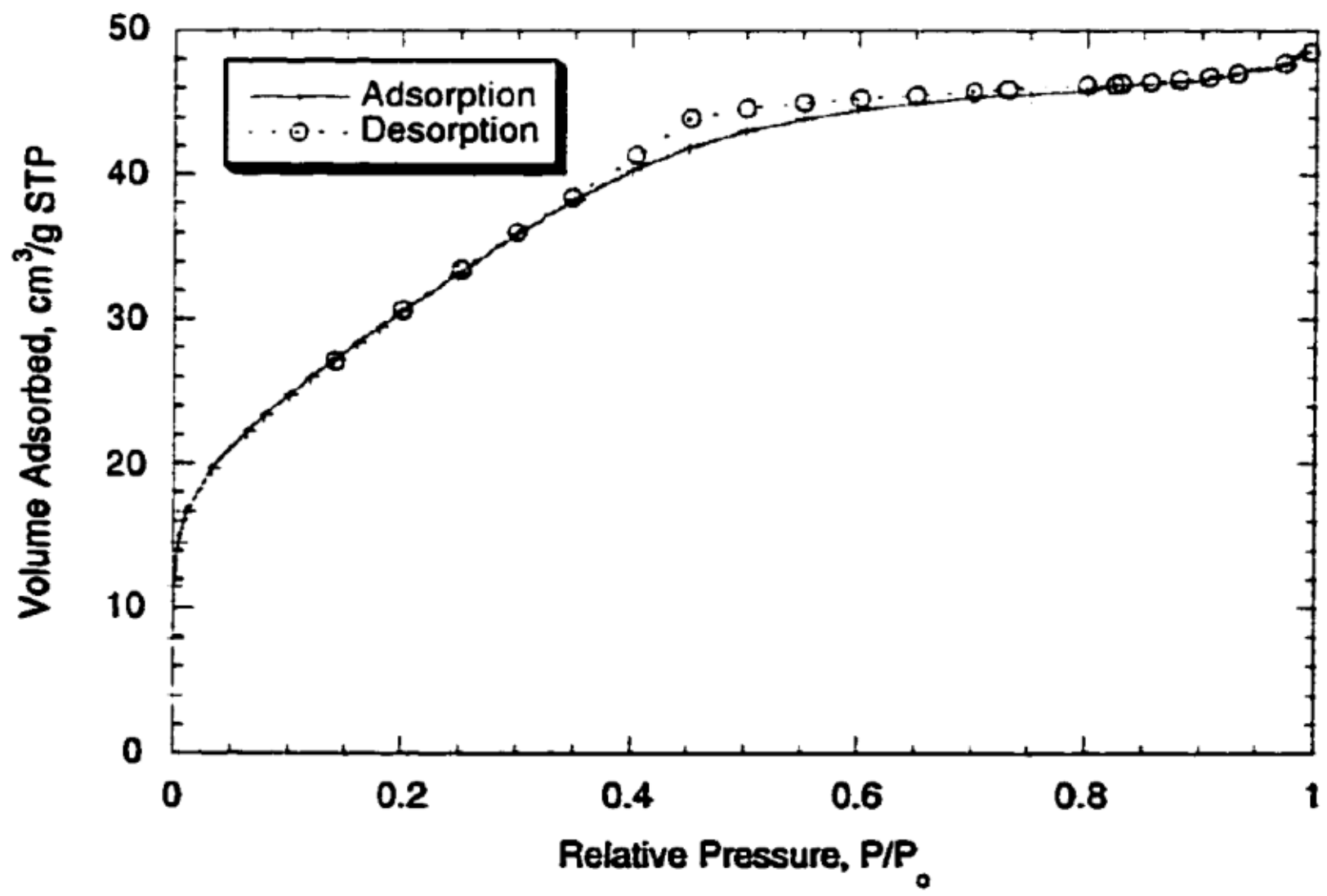

Figure S14: Low Cr nickel catalyst N_2 adsorption and desorption isotherms at 77 K.

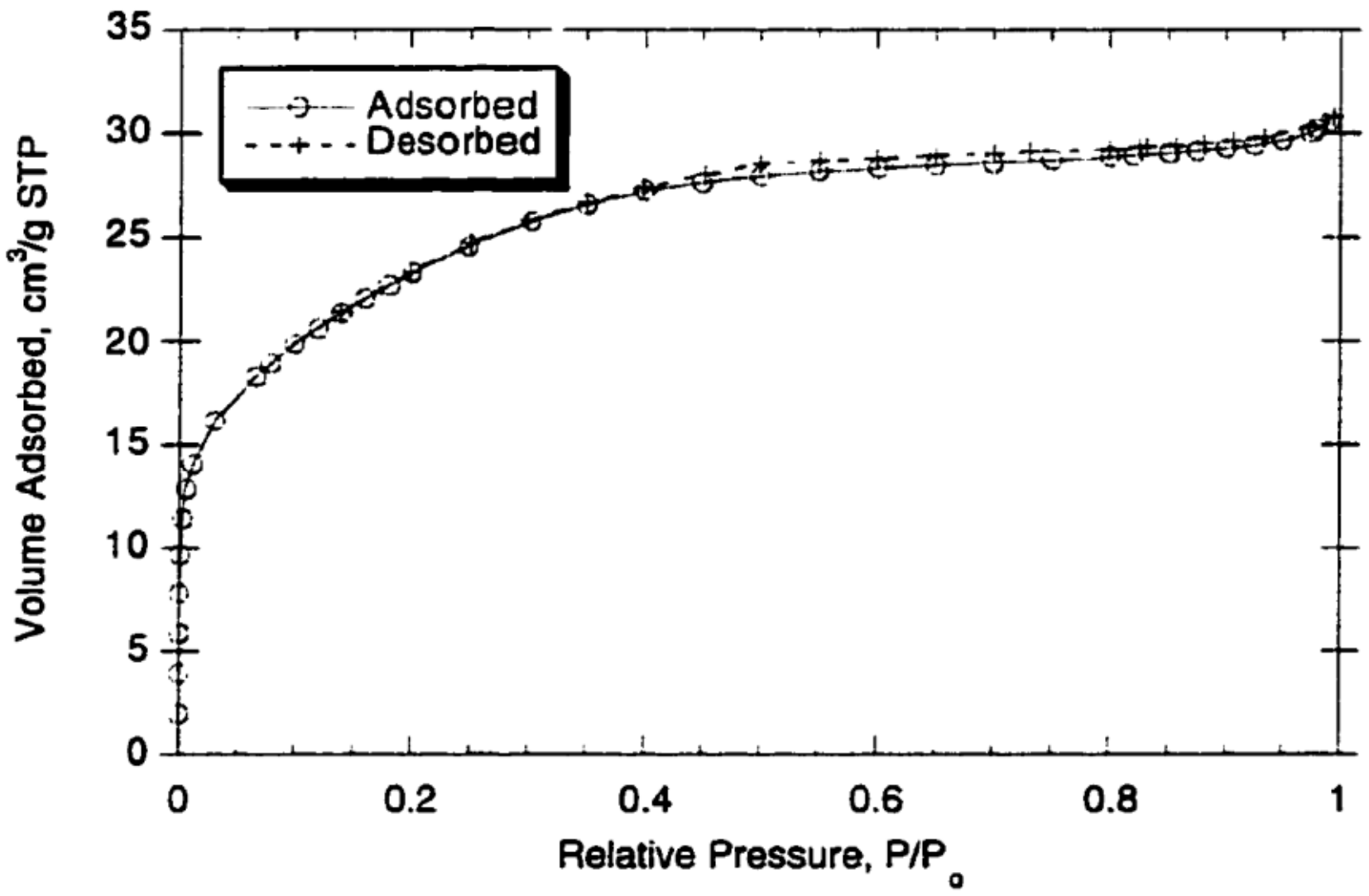

Figure S15: High Cr nickel catalyst N_2 adsorption and desorption isotherms at 77 K.

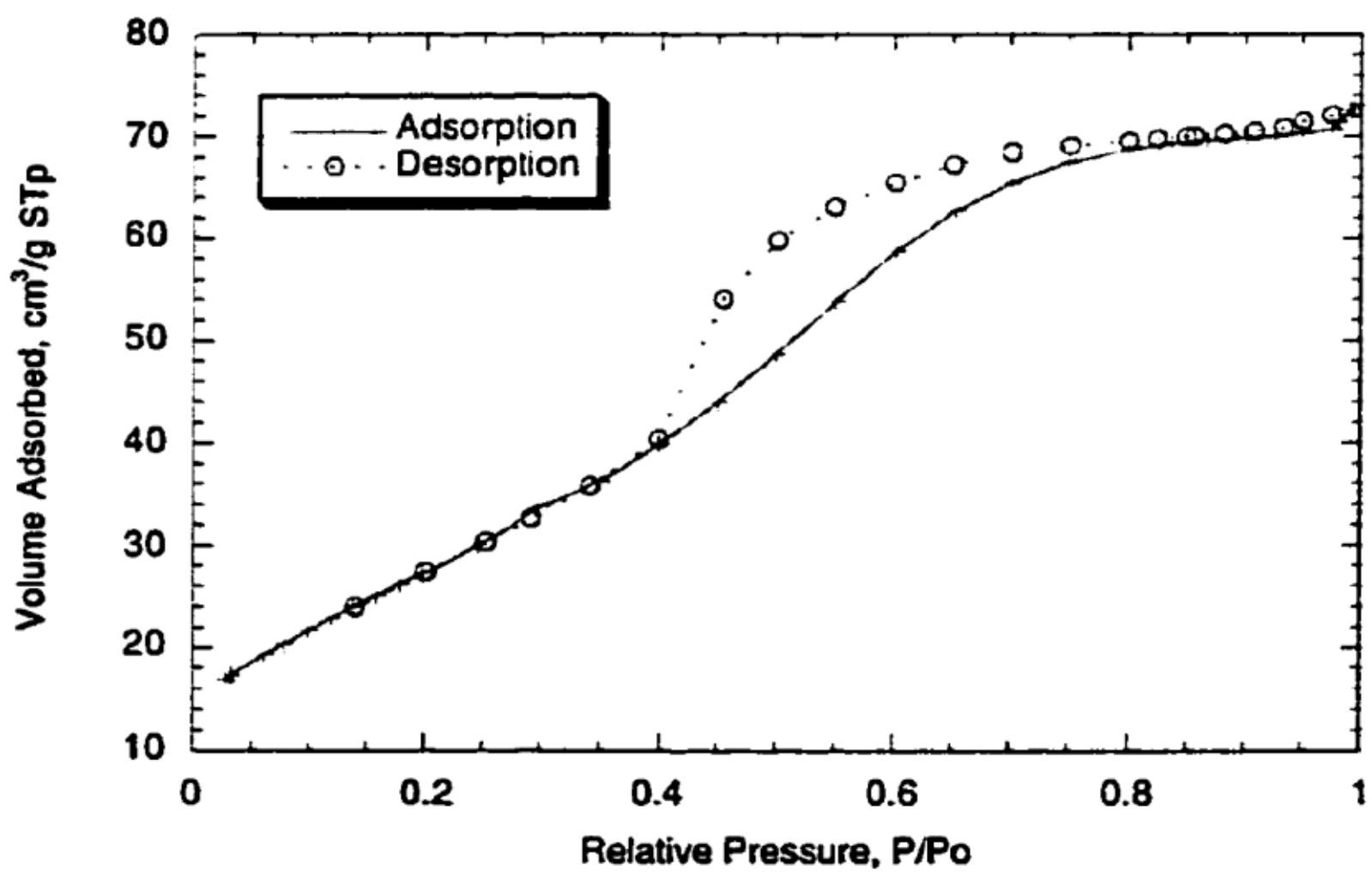

Figure S16: Low Fe nickel catalyst N_2 adsorption and desorption isotherms at 77 K.

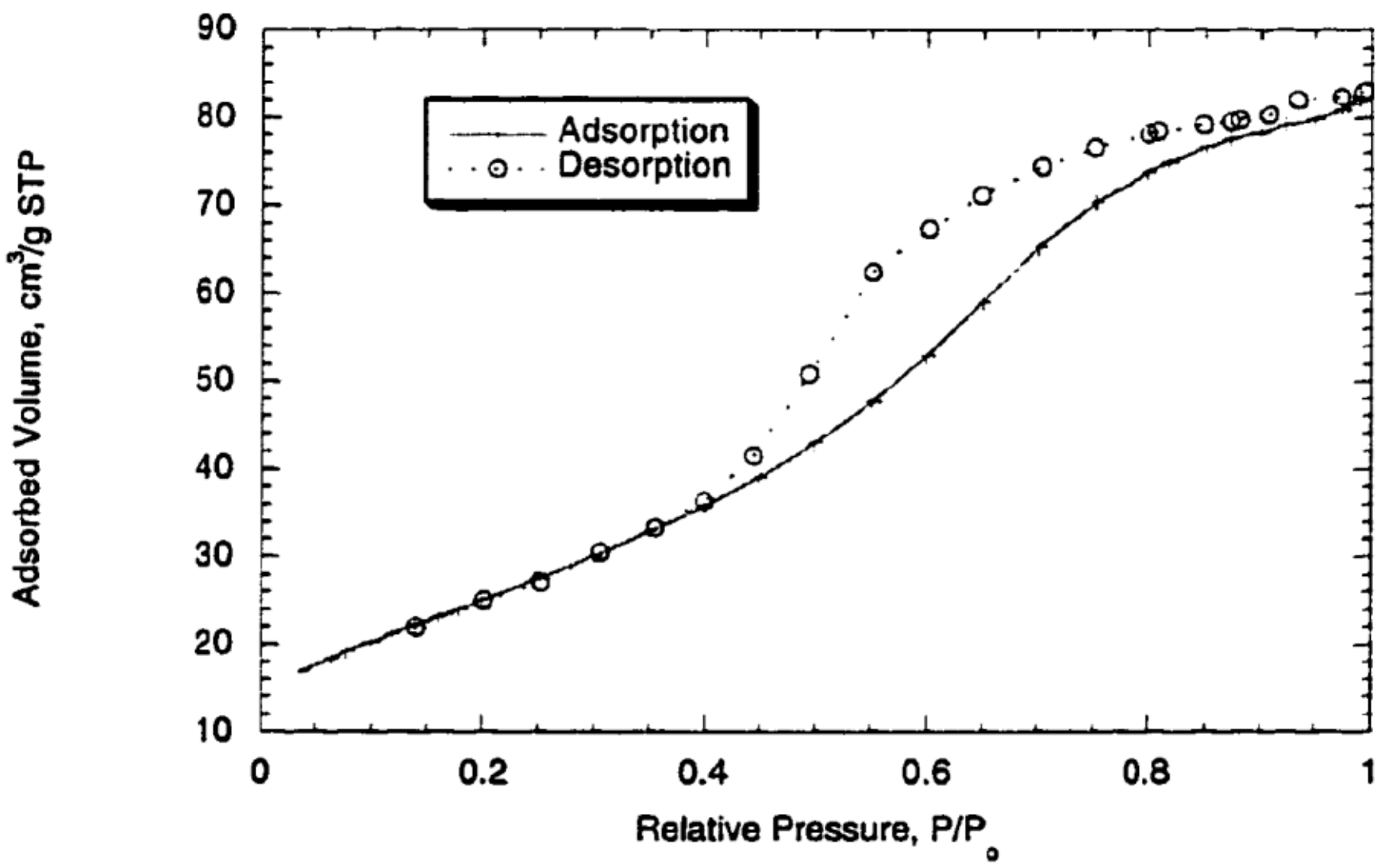

Figure S17: High Fe nickel catalyst N_2 adsorption and desorption isotherms at 77 K.

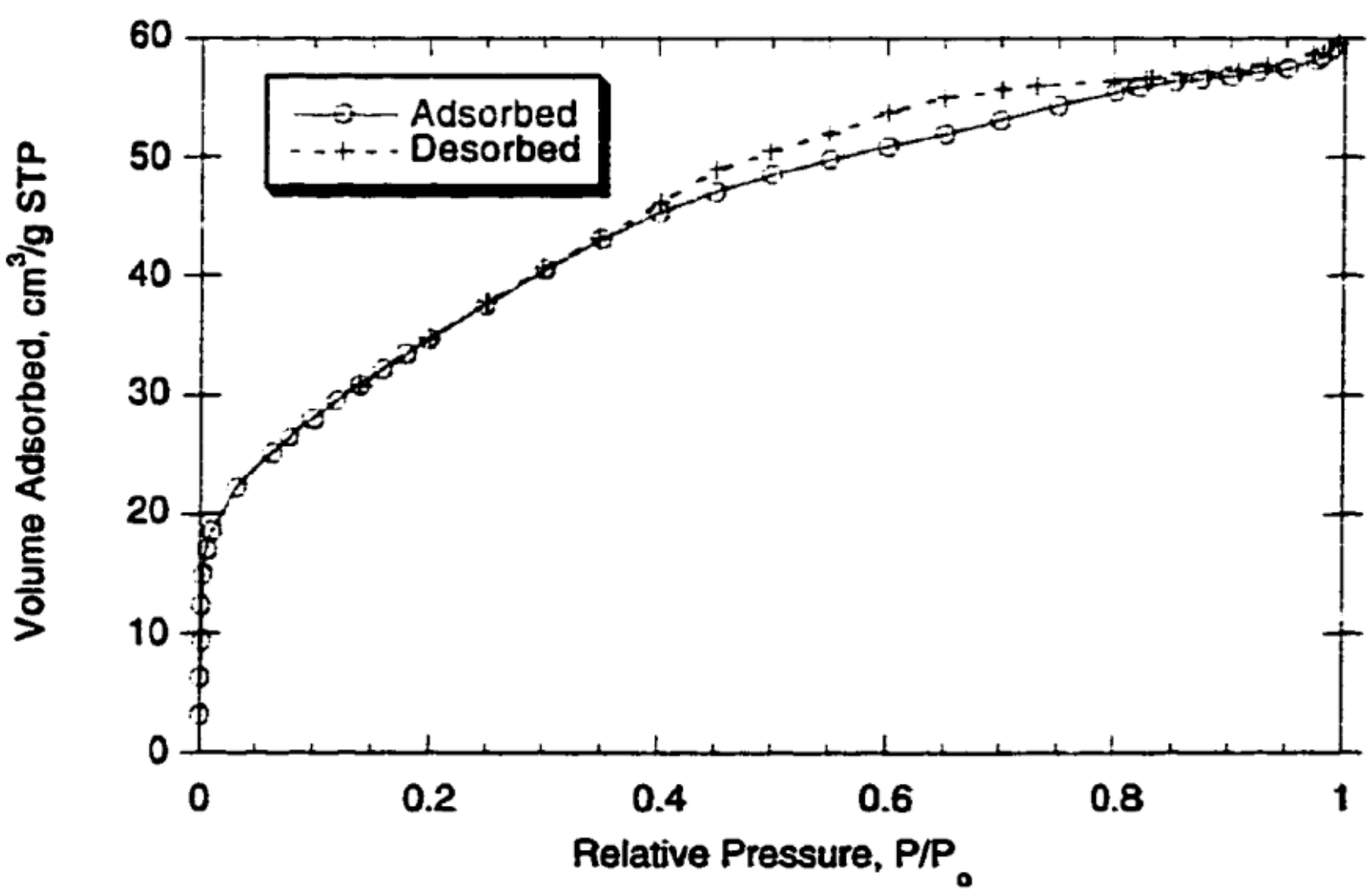

Figure S18: Cr, Fe doubly-promoted nickel catalyst N_2 adsorption and desorption isotherms at 77 K.

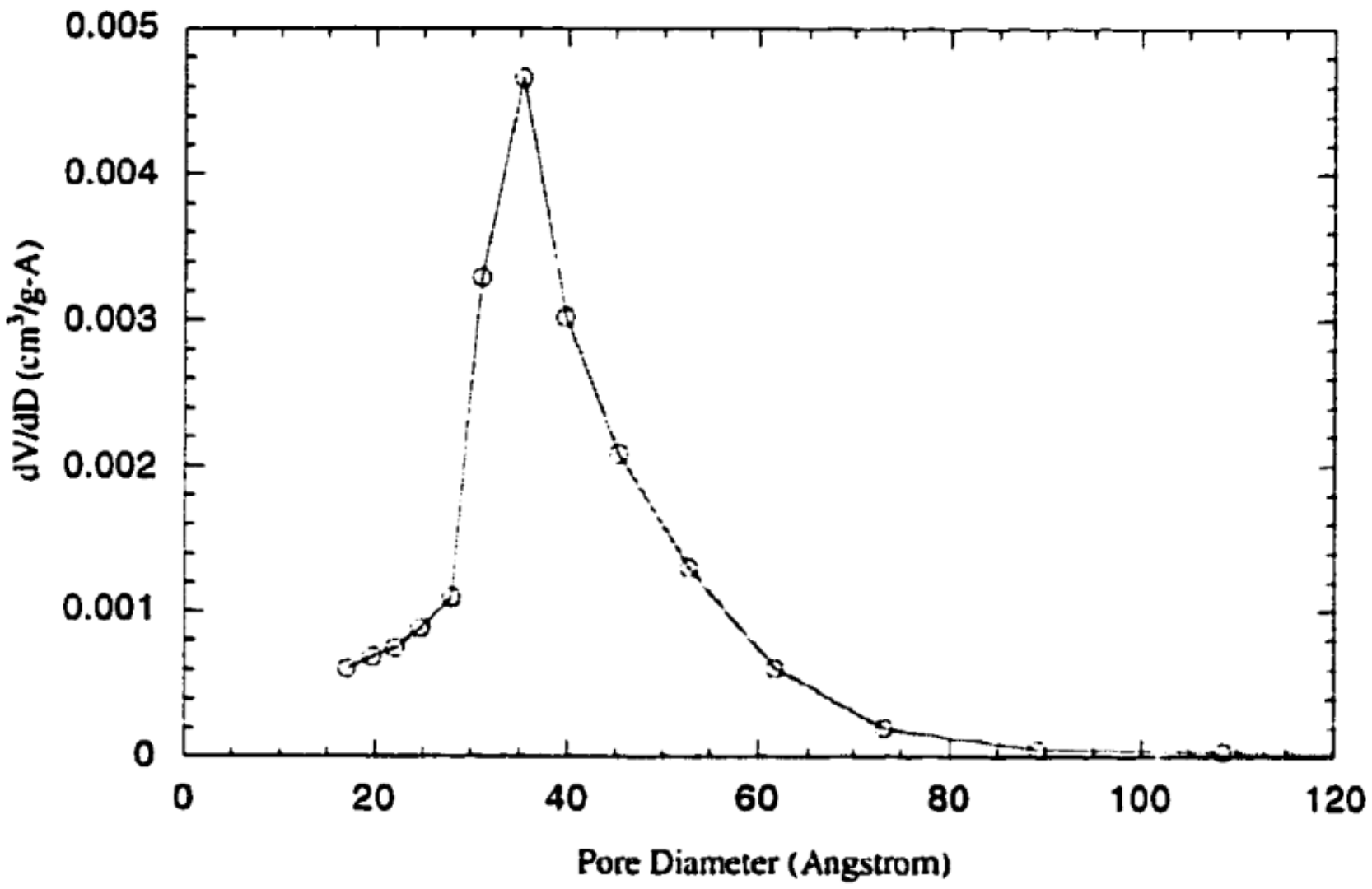

Figure S19: Distribution of pore volume versus pore diameter for the unpromoted nickel catalyst formed by leaching the Purdue alloy. This distribution was extracted from the N_2 desorption isotherm at 77 K using the Barrett, Joyner, and Halenda (BJH[S1]) method.

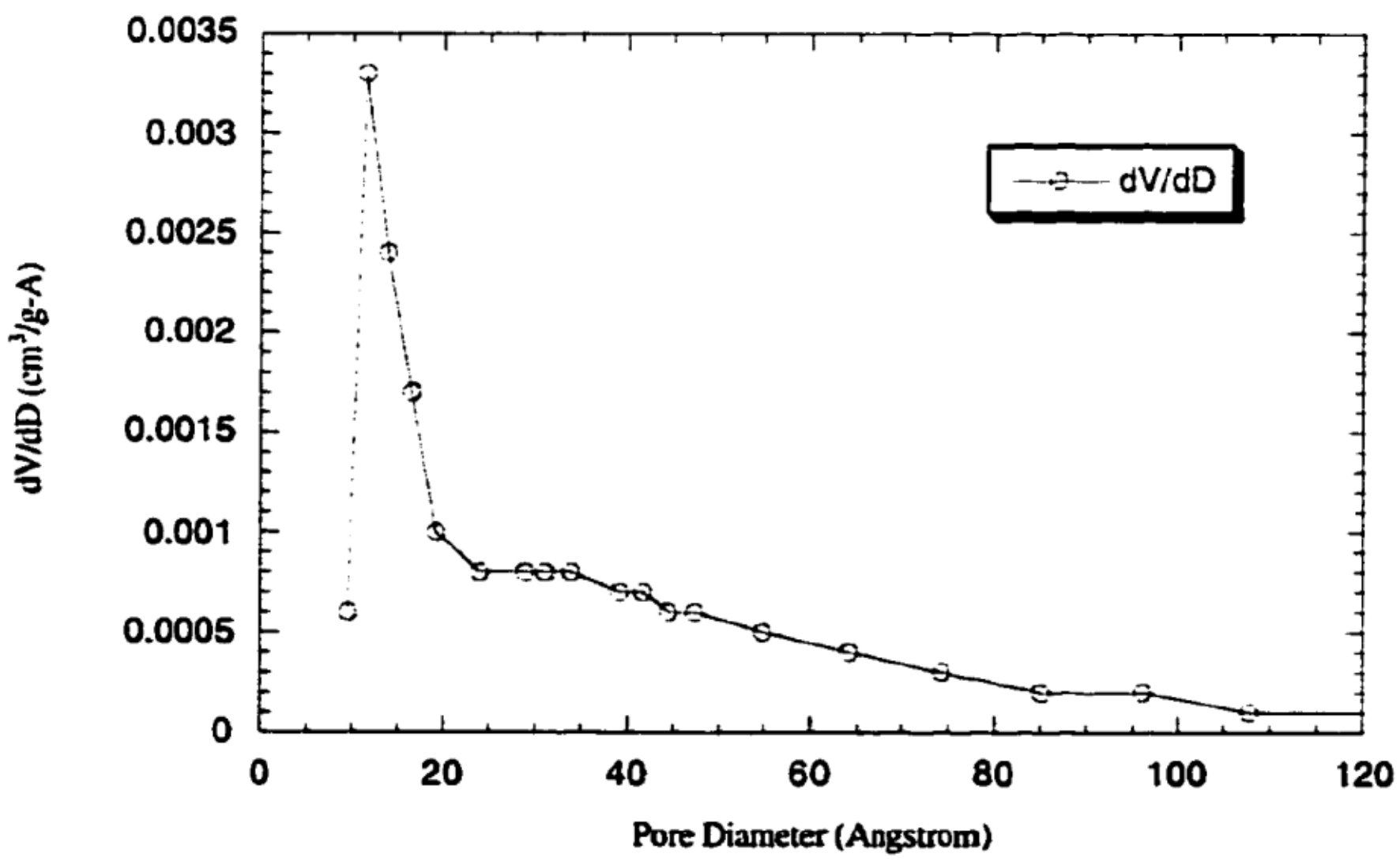

Figure S20: Distribution of pore volume versus pore diameter for the low Cr nickel catalyst formed by leaching the Purdue alloy. This distribution was extracted from the N_2 desorption isotherm at 77 K using the Barrett, Joyner, and Halenda (BJH[S1]) method.

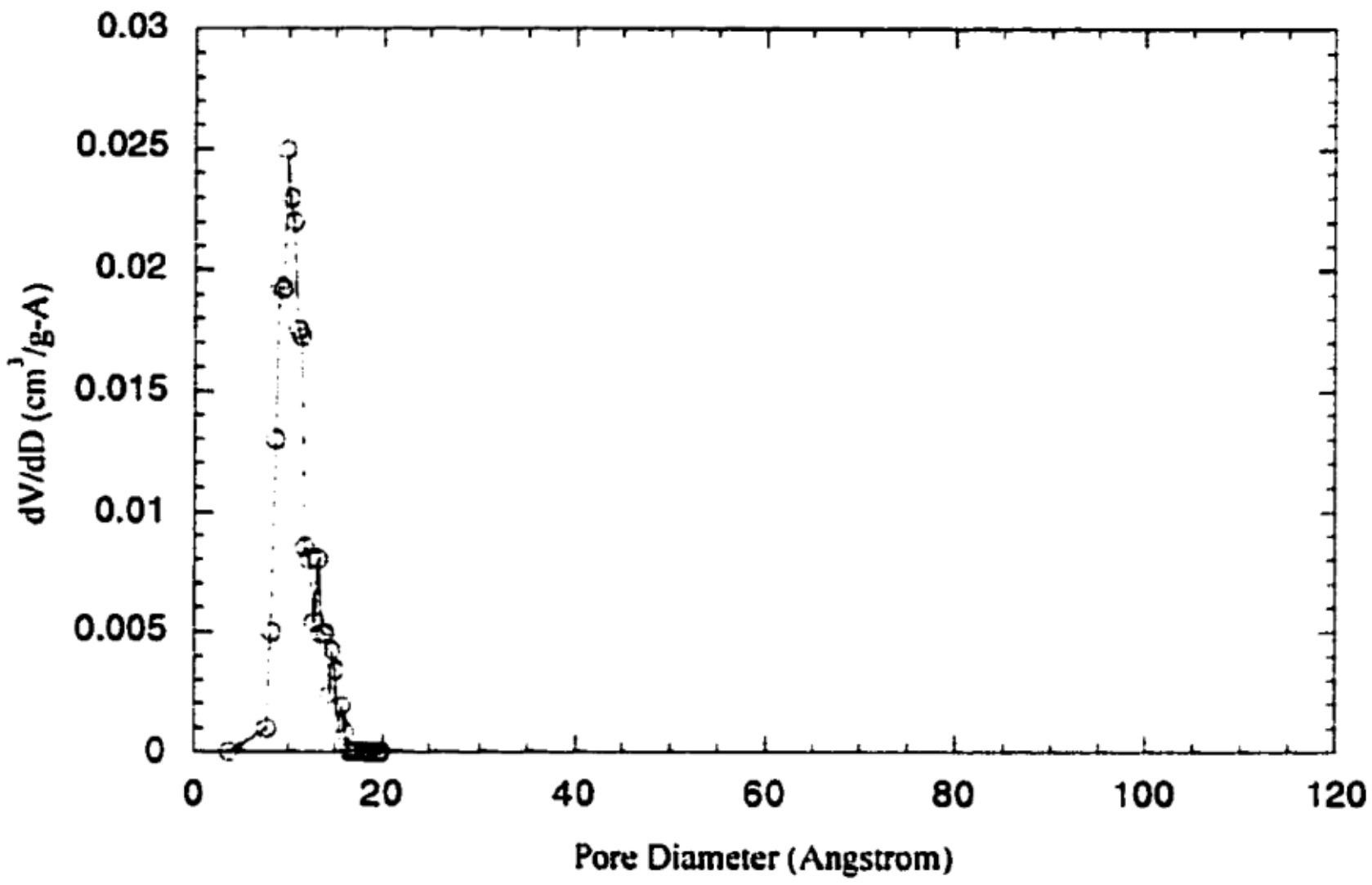

Figure S21: Distribution of pore volume versus pore diameter for the high Cr nickel catalyst formed by leaching the Purdue alloy. This distribution was extracted from the N_2 desorption isotherm at 77 K using the Barrett, Joyner, and Halenda (BJH[S1]) method.

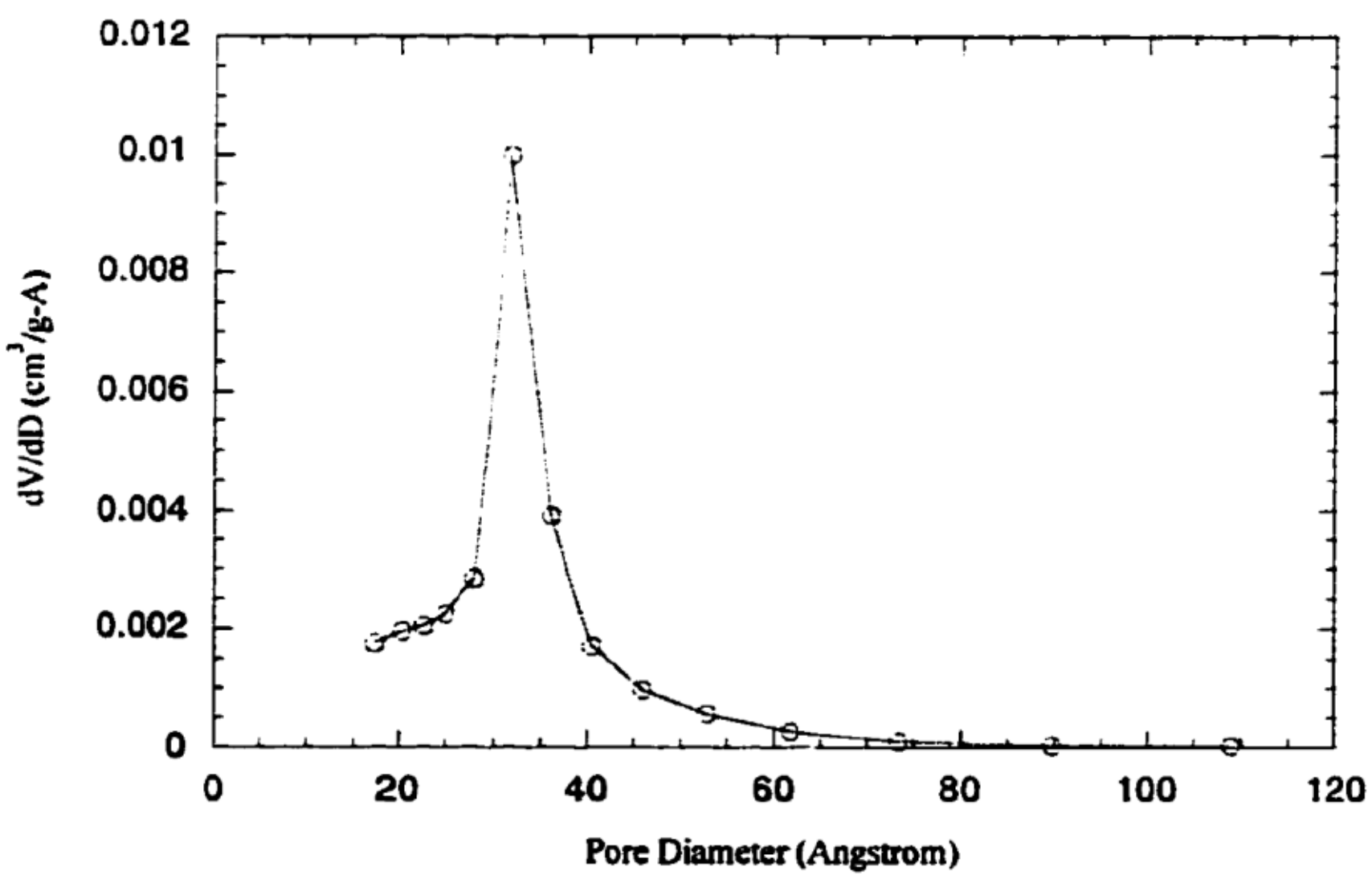

Figure S22: Distribution of pore volume versus pore diameter for the low Fe nickel catalyst formed by leaching the Purdue alloy. This distribution was extracted from the N_2 desorption isotherm at 77 K using the Barrett, Joyner, and Halenda (BJH[S1]) method.

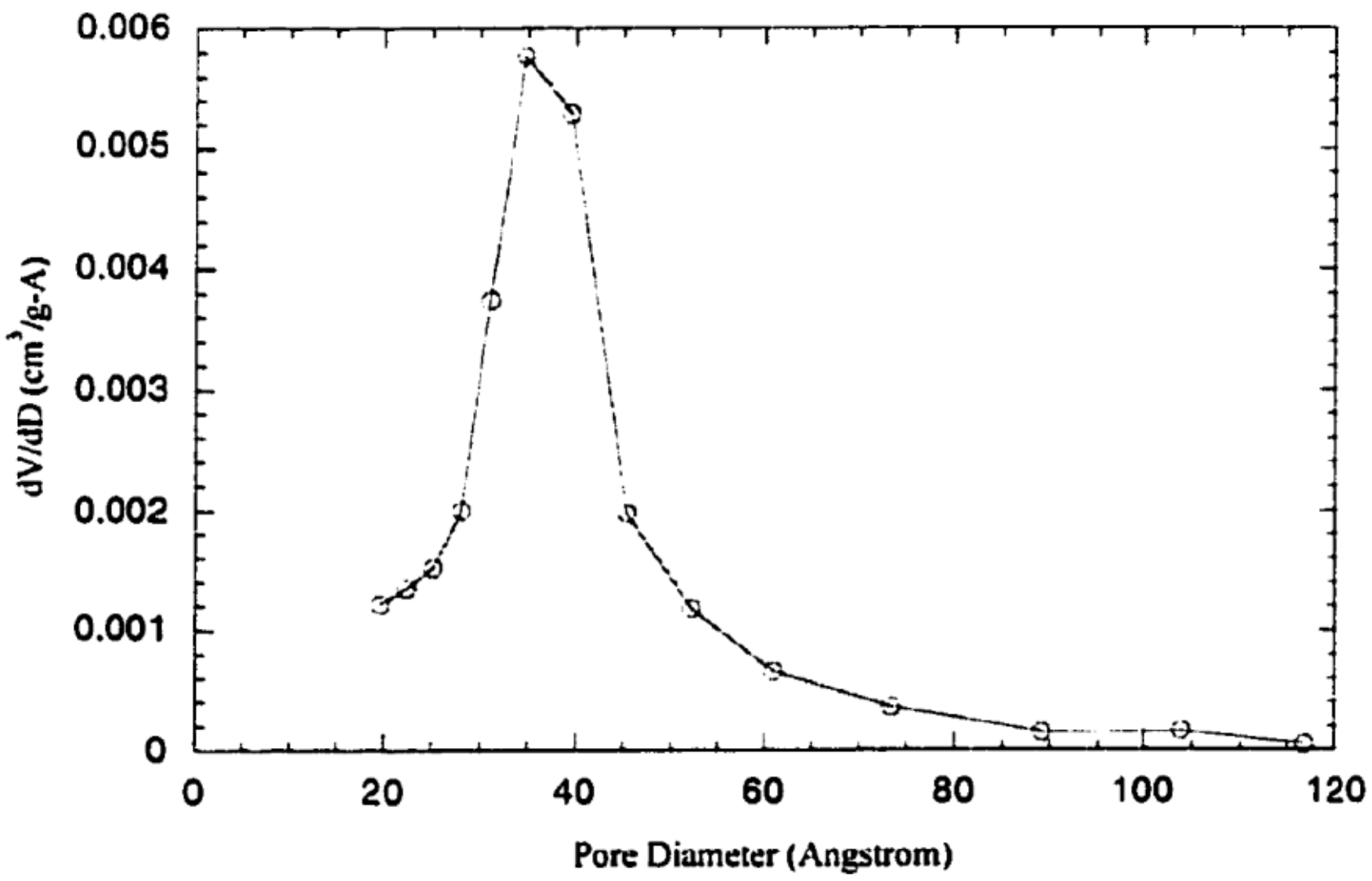

Figure S23: Distribution of pore volume versus pore diameter for the high Fe nickel catalyst formed by leaching the Purdue alloy. This distribution was extracted from the N_2 desorption isotherm at 77 K using the Barrett, Joyner, and Halenda (BJH[S1]) method.

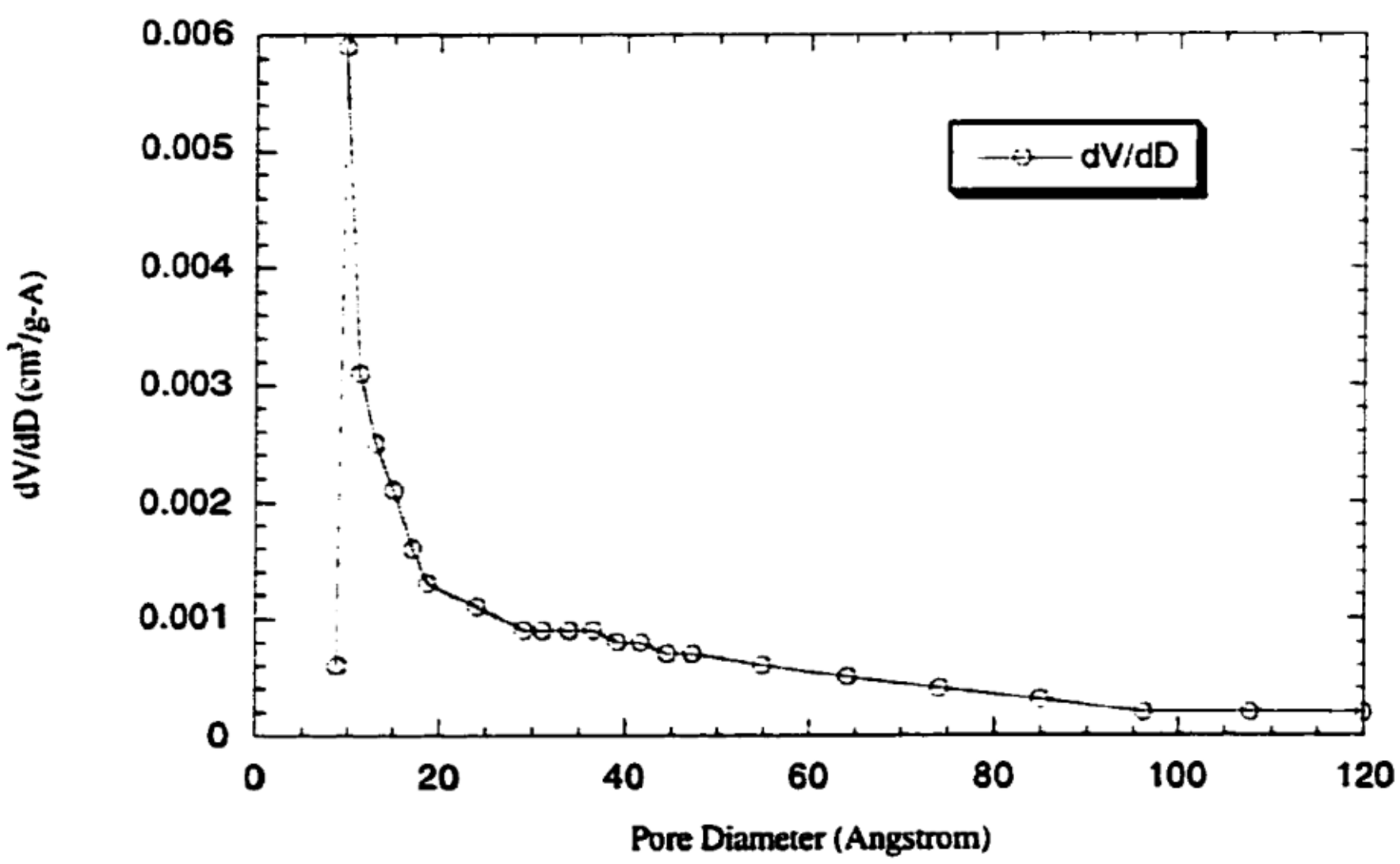

Figure S24: Distribution of pore volume versus pore diameter for the Cr, Fe doubly-promoted nickel catalyst formed by leaching the Purdue alloy. This distribution was extracted from the N_2 desorption isotherm at 77 K using the Barrett, Joyner, and Halenda (BJH[S1]) method.

S4. Carbon monoxide chemisorption isotherms

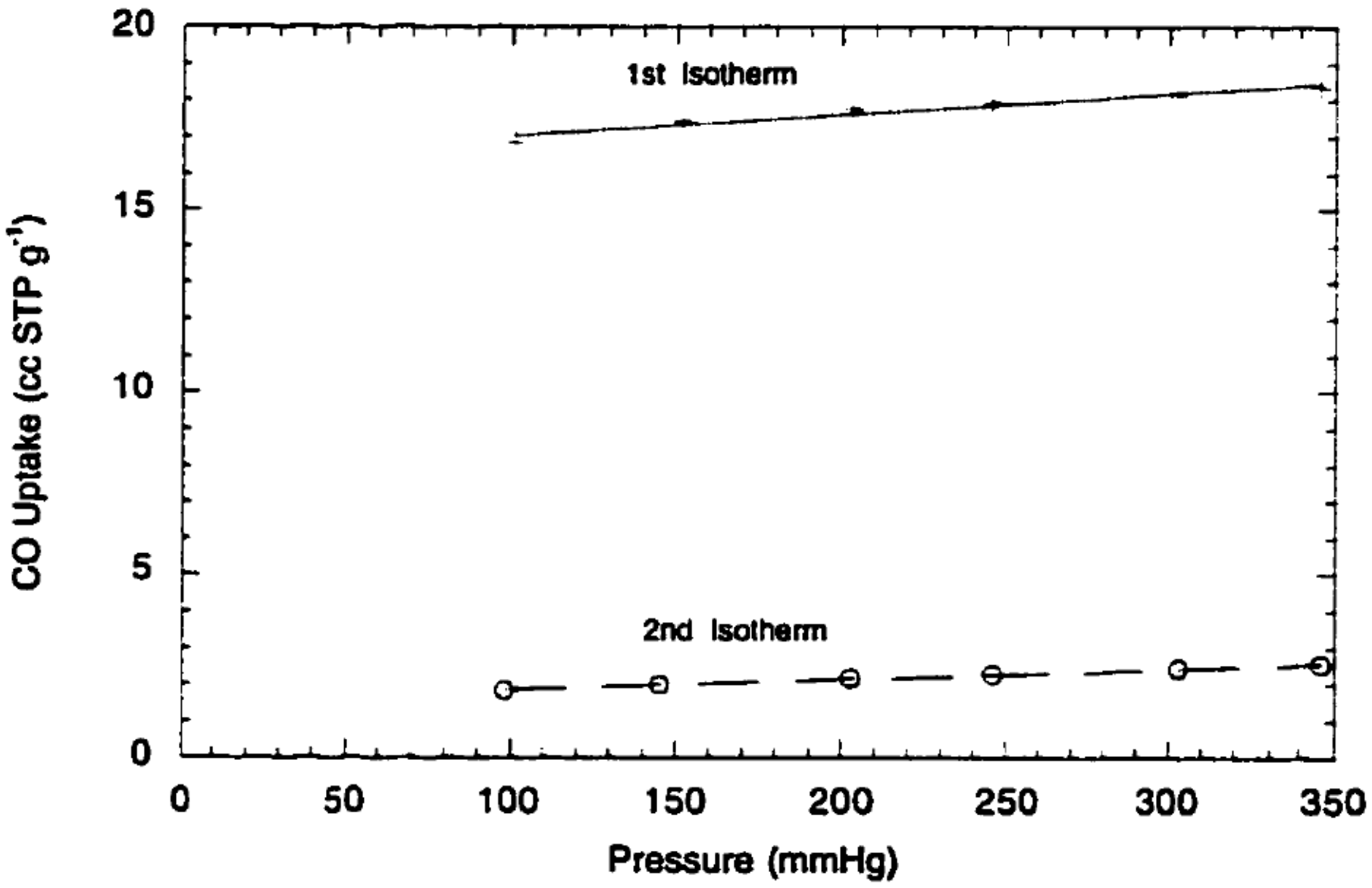

Figure S25: CO adsorption isotherm on unpromoted catalyst.

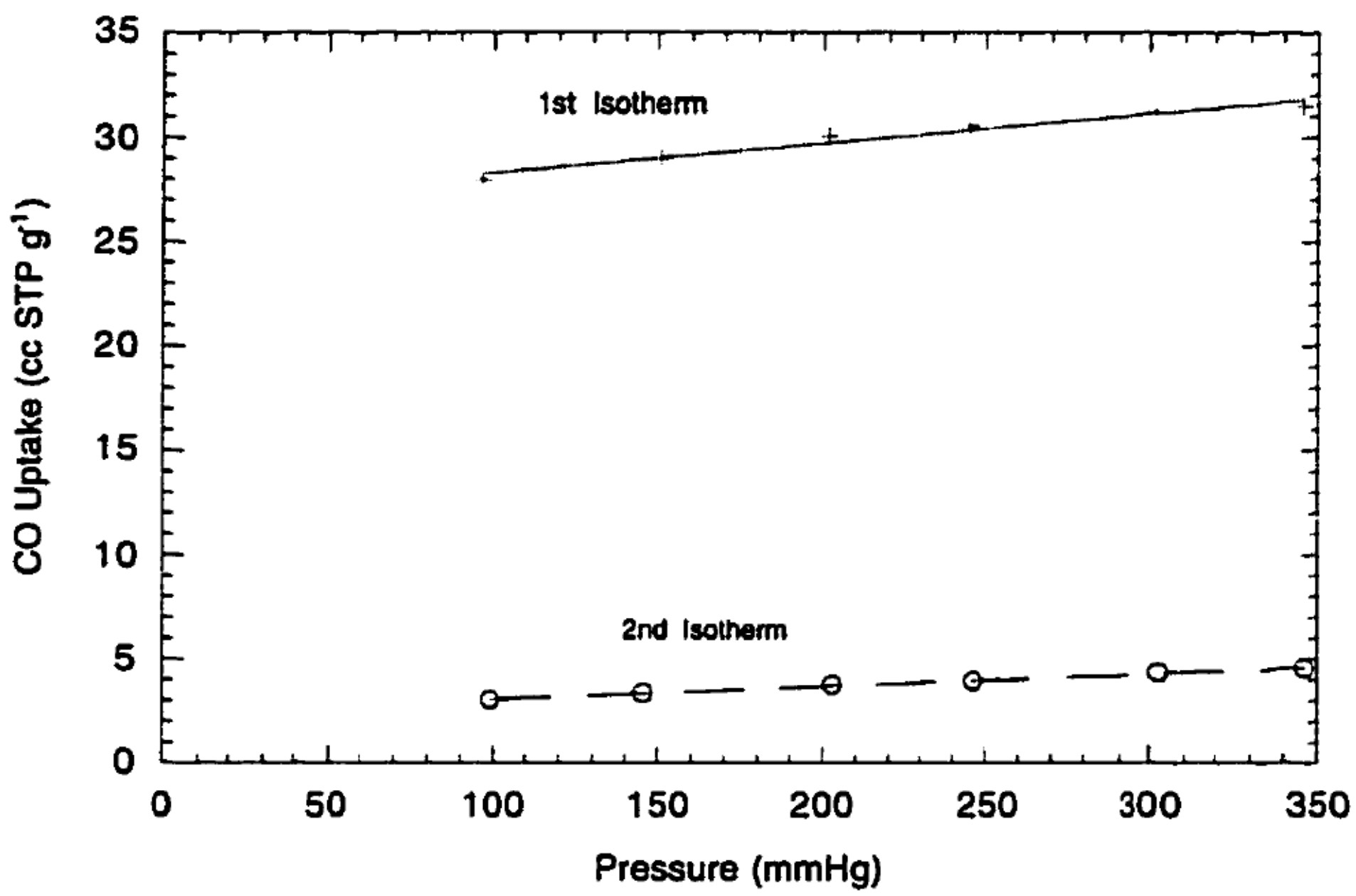

Figure S26: CO adsorption isotherm on low Cr catalyst.

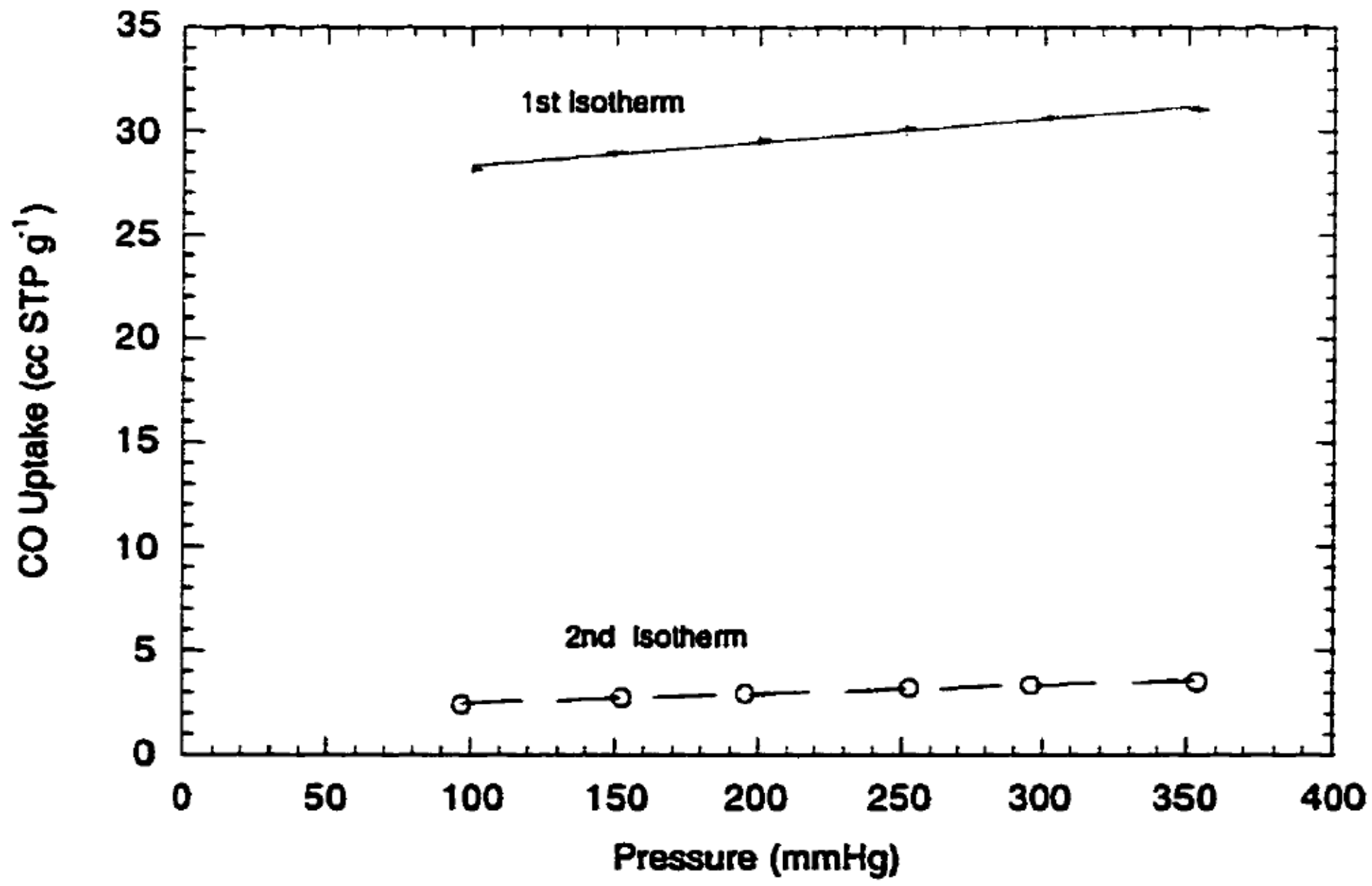

Figure S27: CO adsorption isotherm on high Cr catalyst.

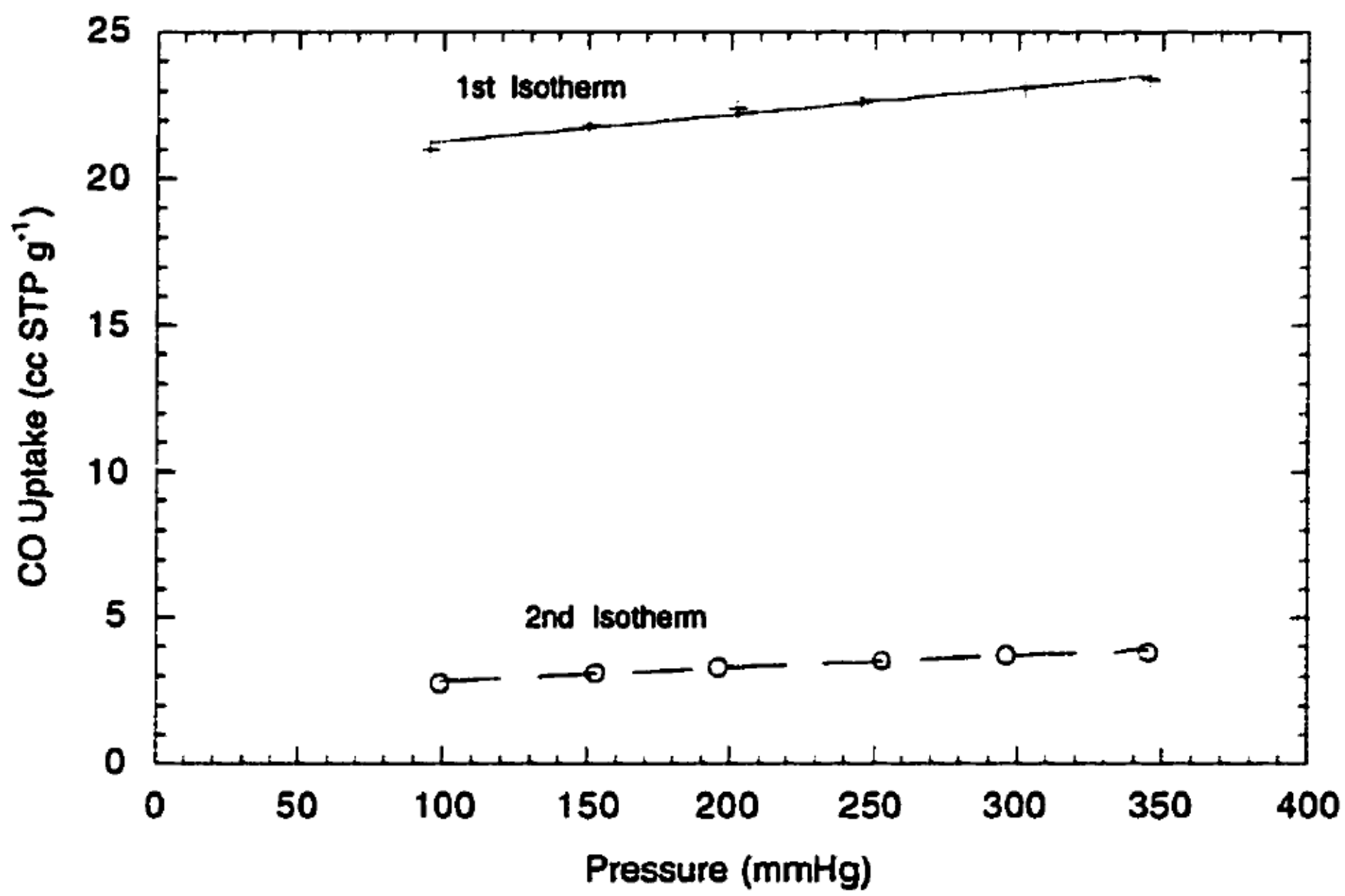

Figure S28: CO adsorption isotherm on low Fe catalyst.

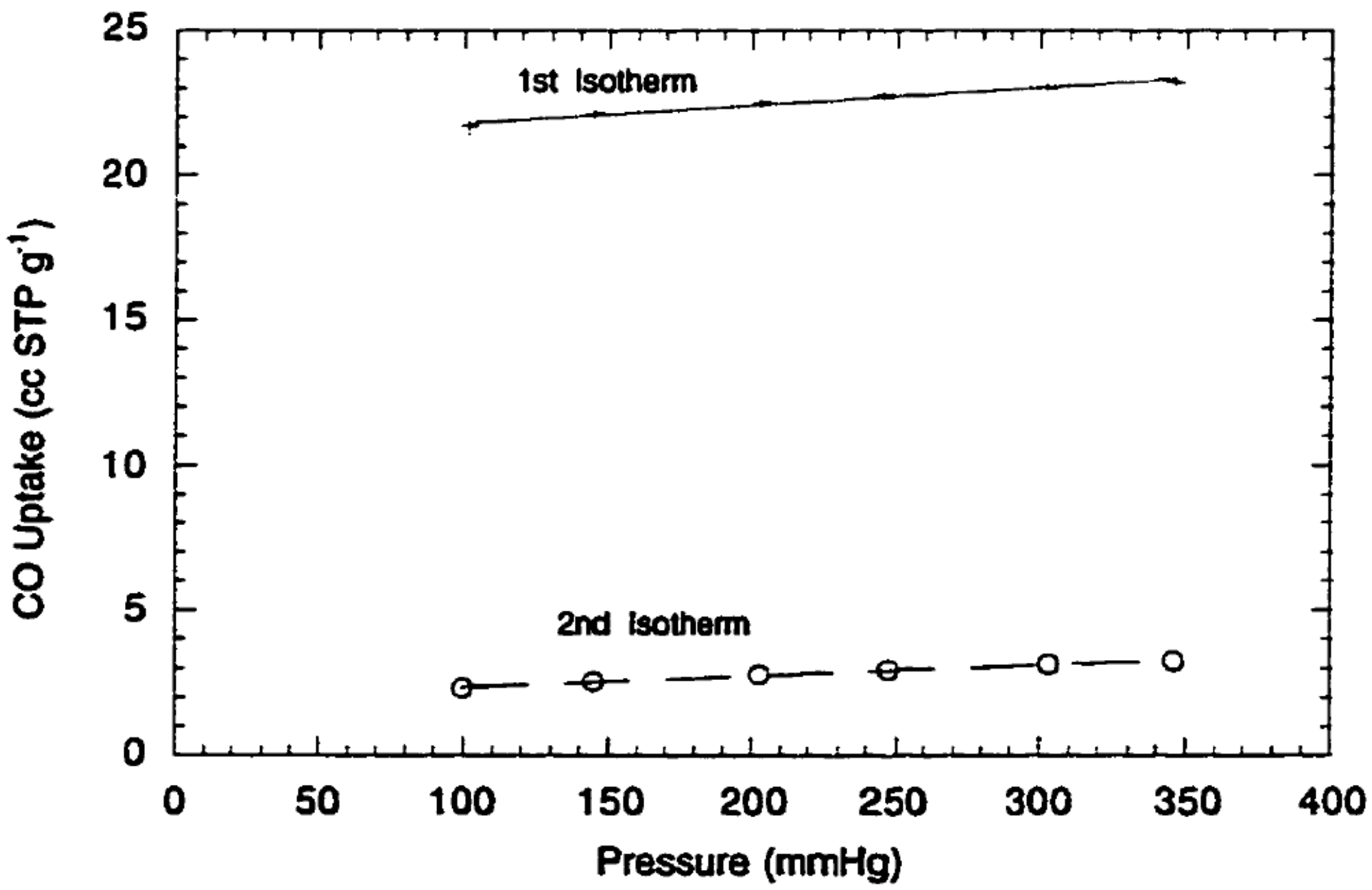

Figure S29: CO adsorption isotherm on high Fe catalyst.

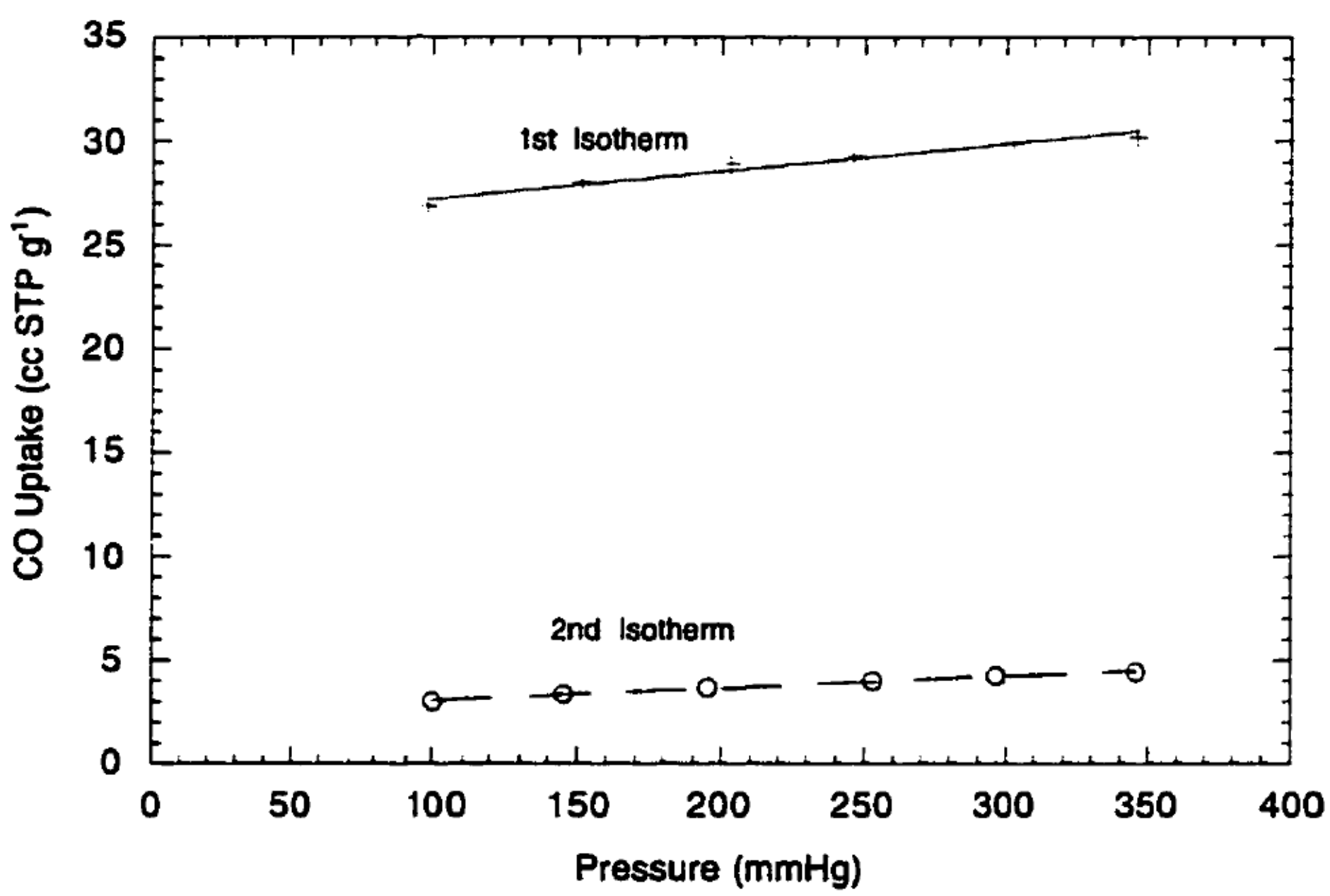

Figure S30: CO adsorption isotherm on Fe, Cr doubly-promoted catalyst.

S5. Hydrogen adsorption isotherms

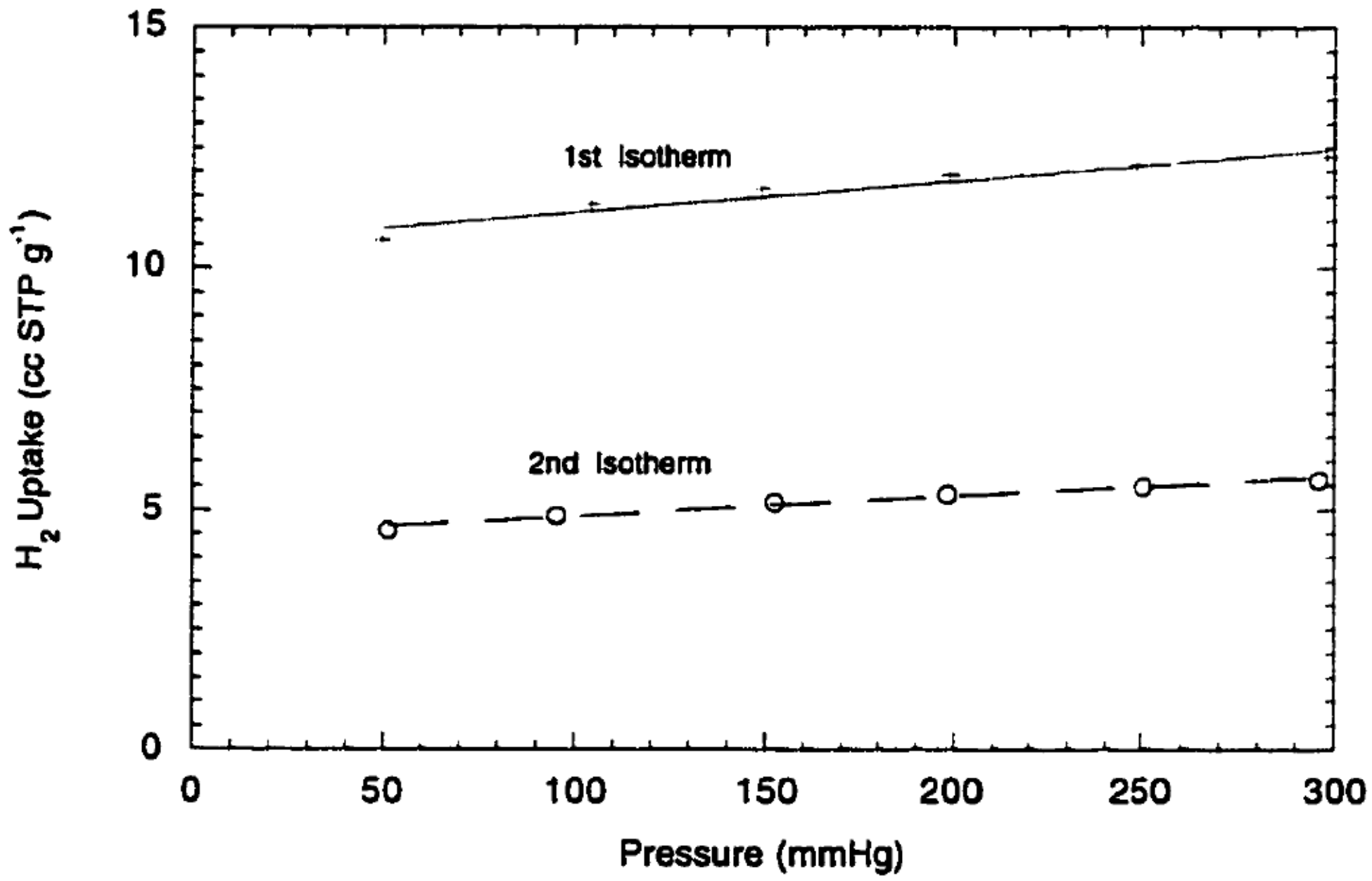

Figure S31: H_2 adsorption isotherm on unpromoted catalyst.

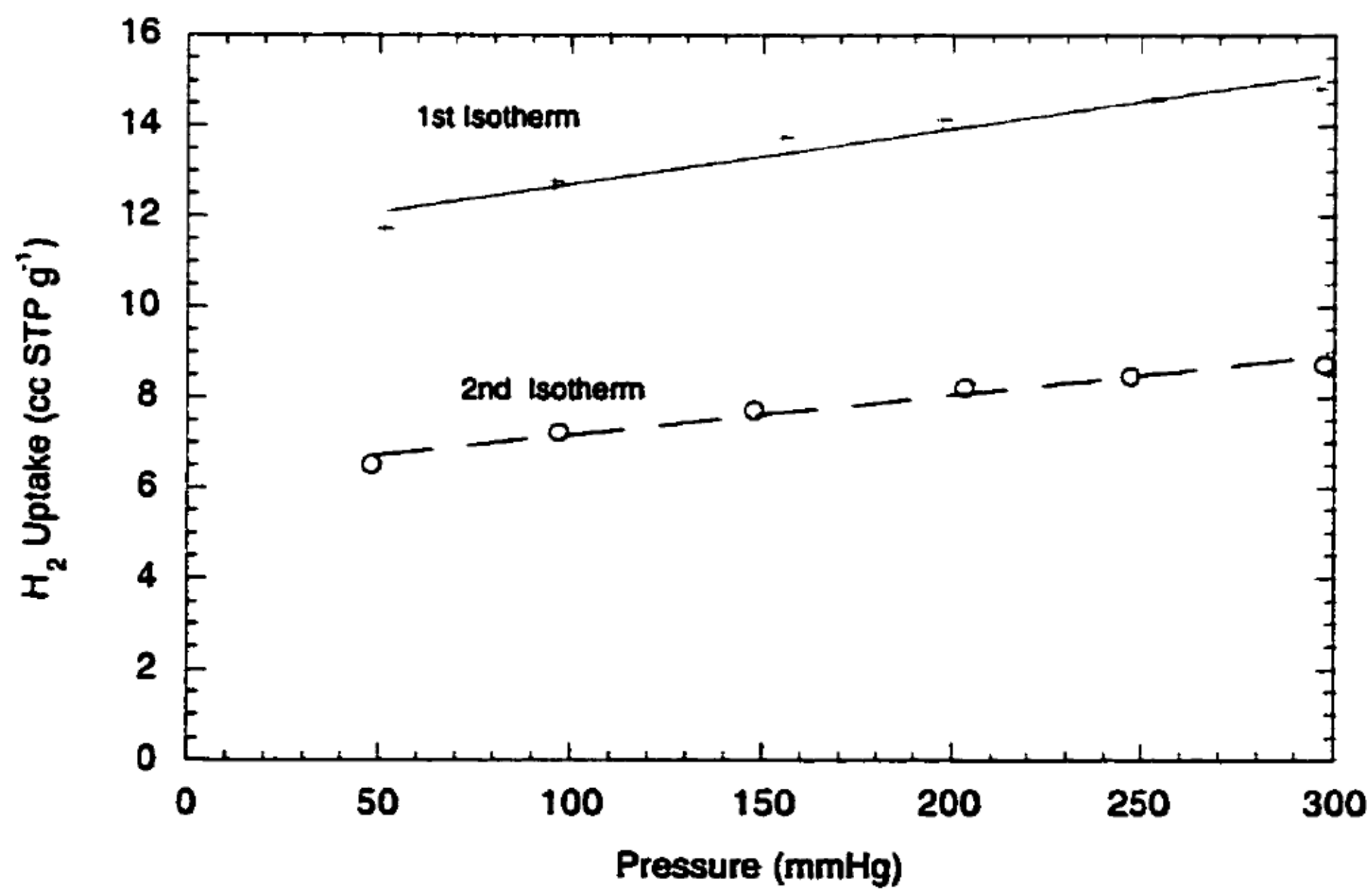

Figure S32: H_2 adsorption isotherm on low Cr catalyst.

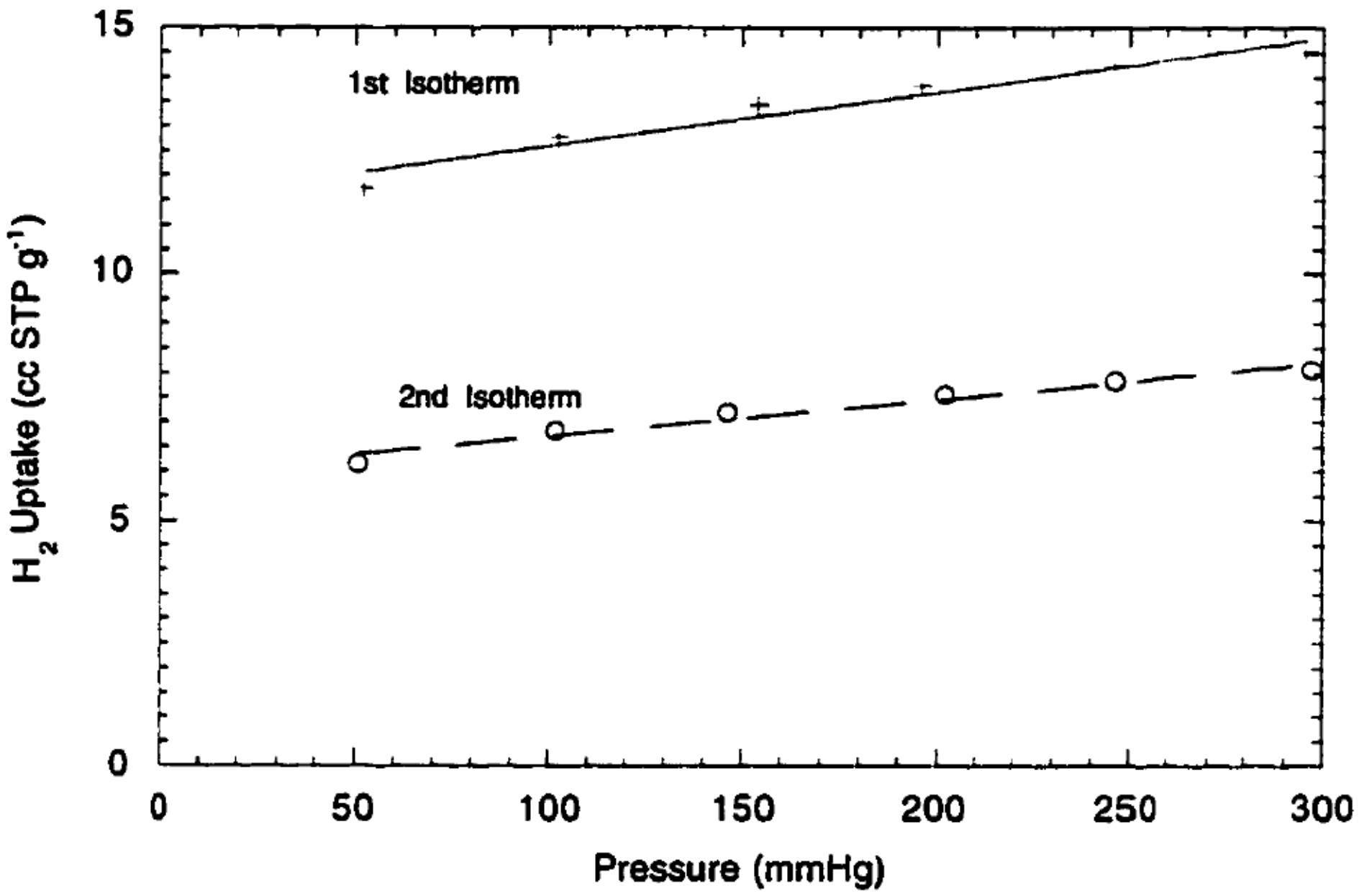

Figure S33: H_2 adsorption isotherm on high Cr catalyst.

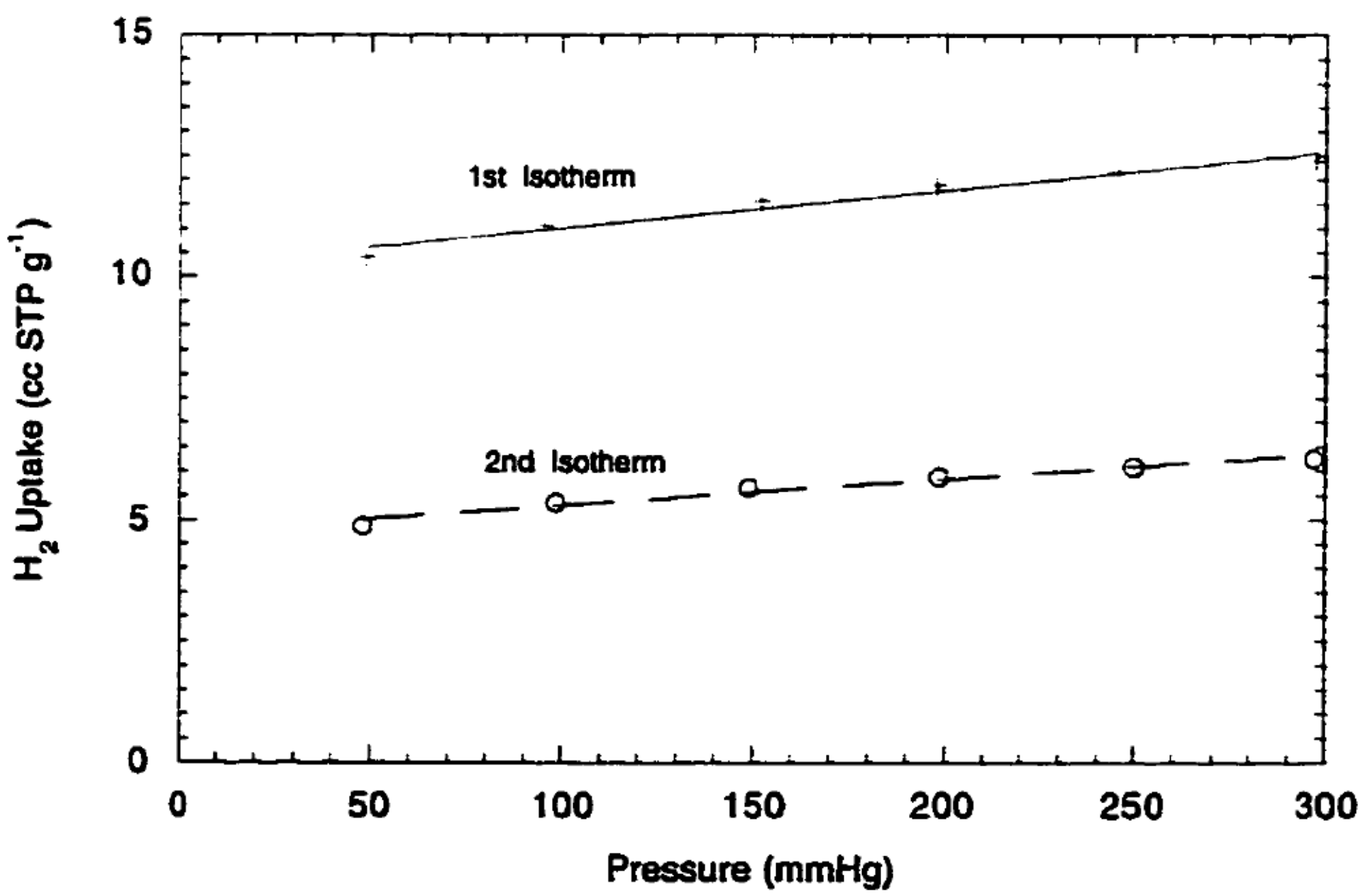

Figure S34: H_2 adsorption isotherm on low Fe catalyst.

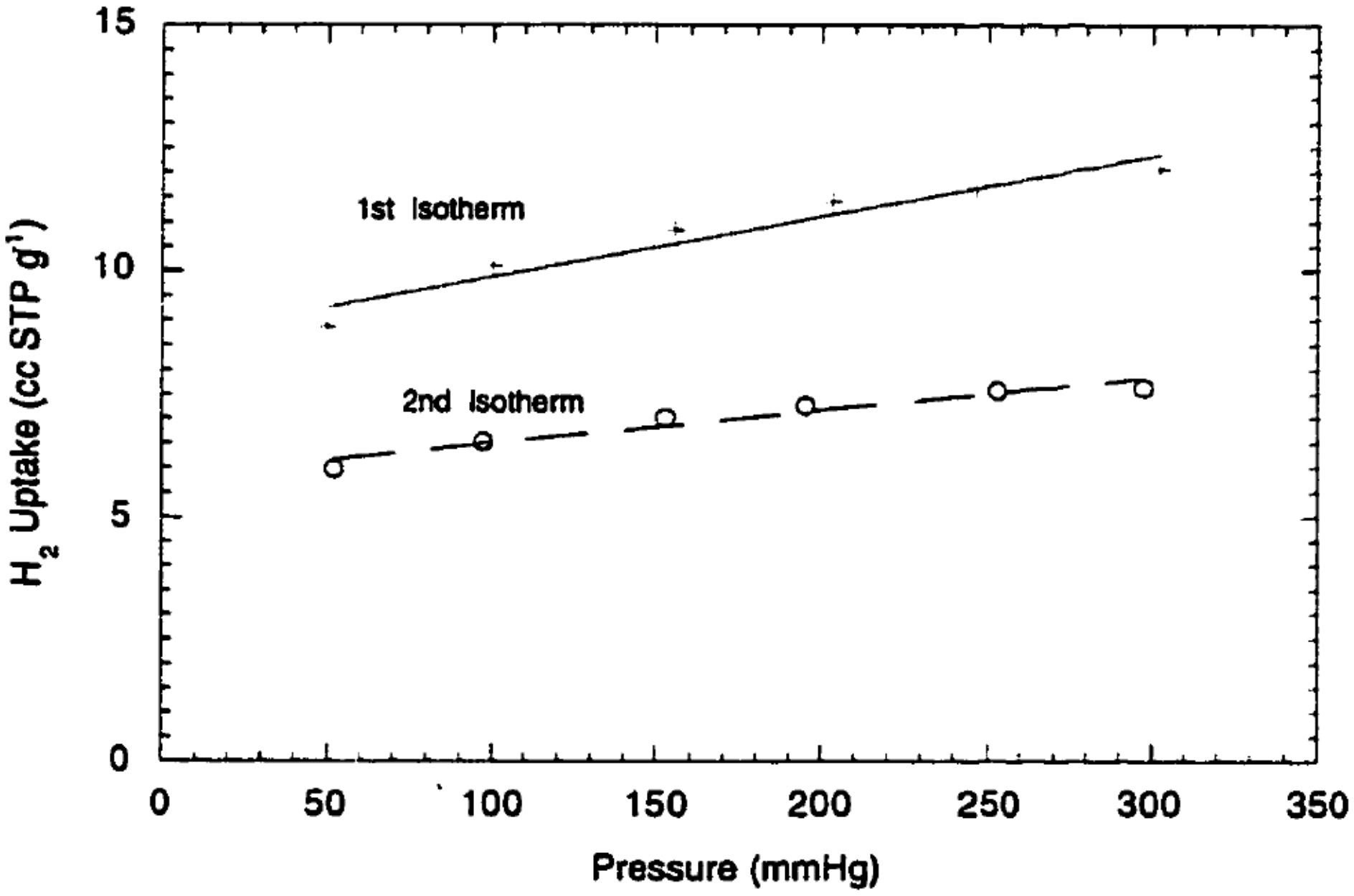

Figure S35: H_2 adsorption isotherm on high Fe catalyst.

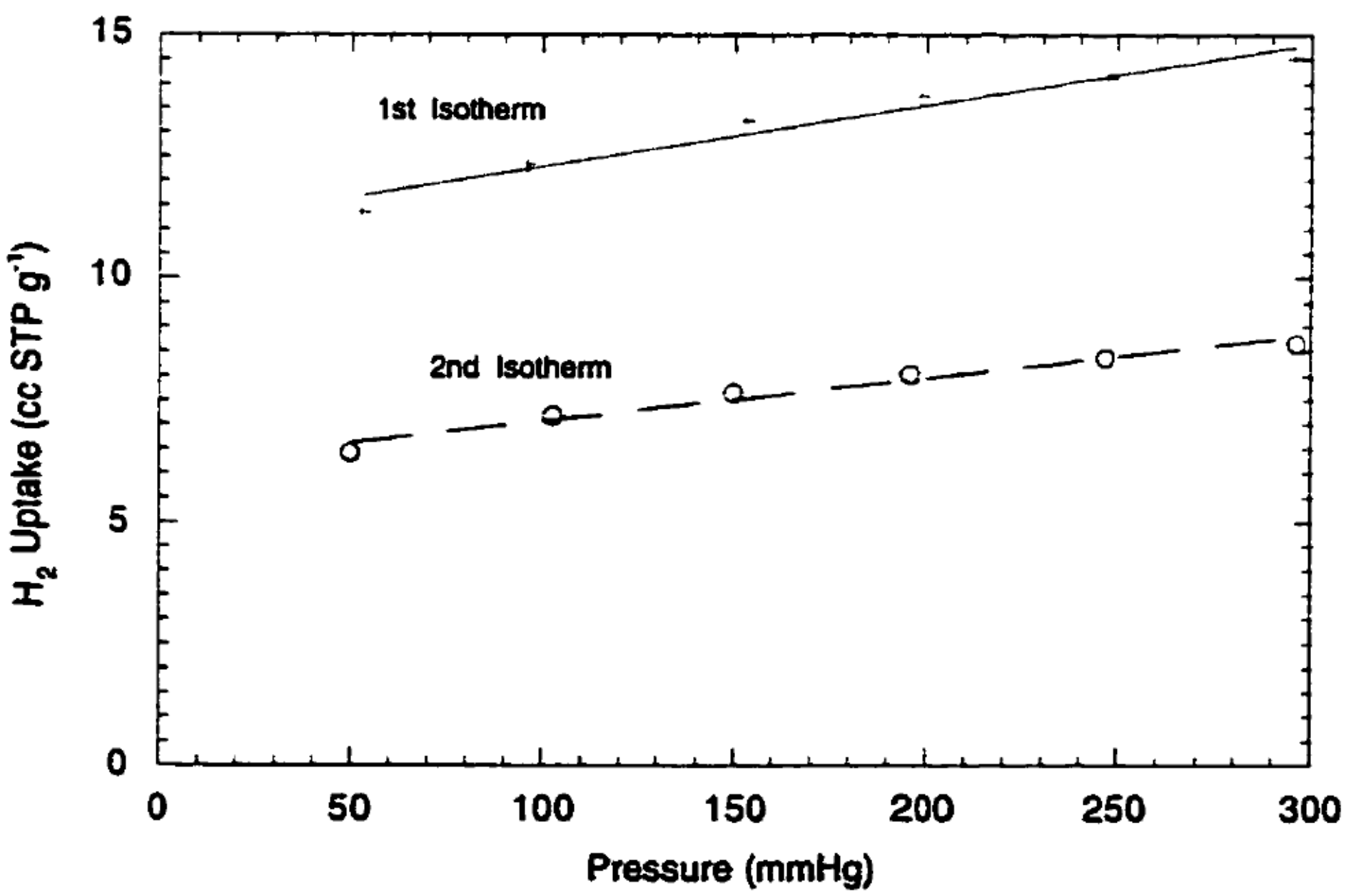

Figure S36: H_2 adsorption isotherm on Fe, Cr doubly-promoted catalyst.

S6. X-ray photoelectron spectroscopy (XPS) curves

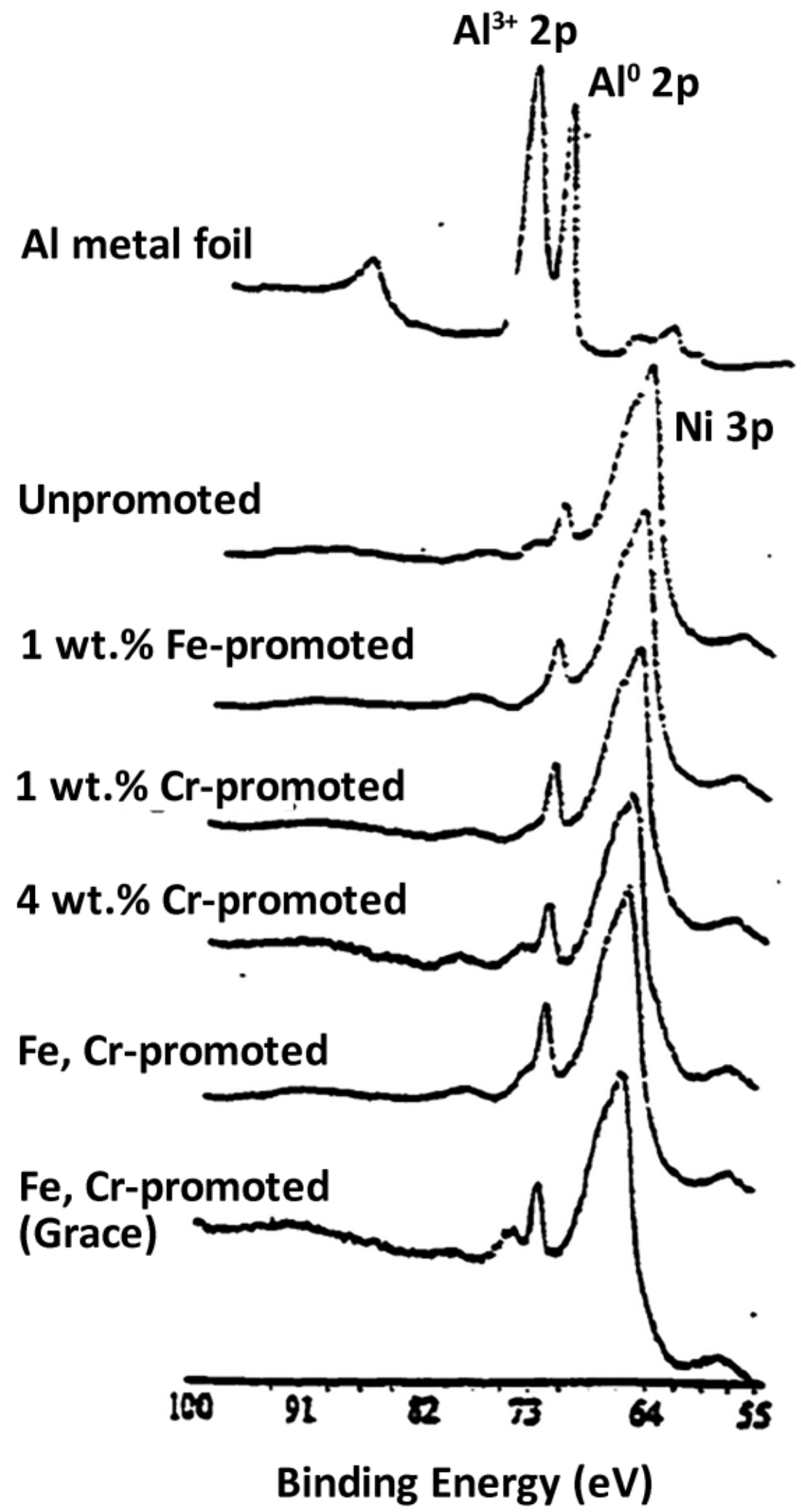

Figure S37: XPS spectra showing the chemical state of aluminum for the active catalysts.

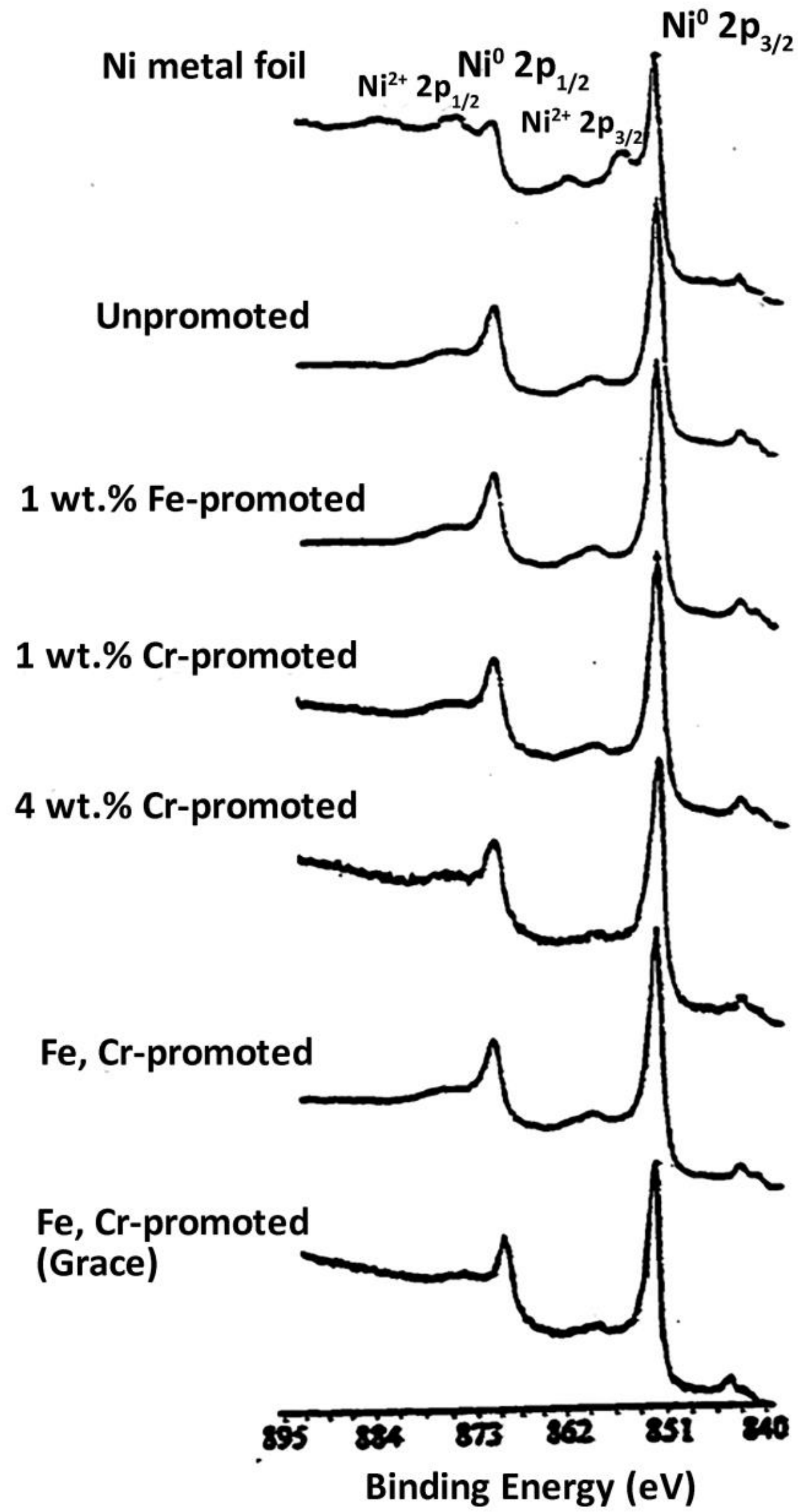

Figure S38: XPS spectra showing the chemical state of nickel for the active catalysts.

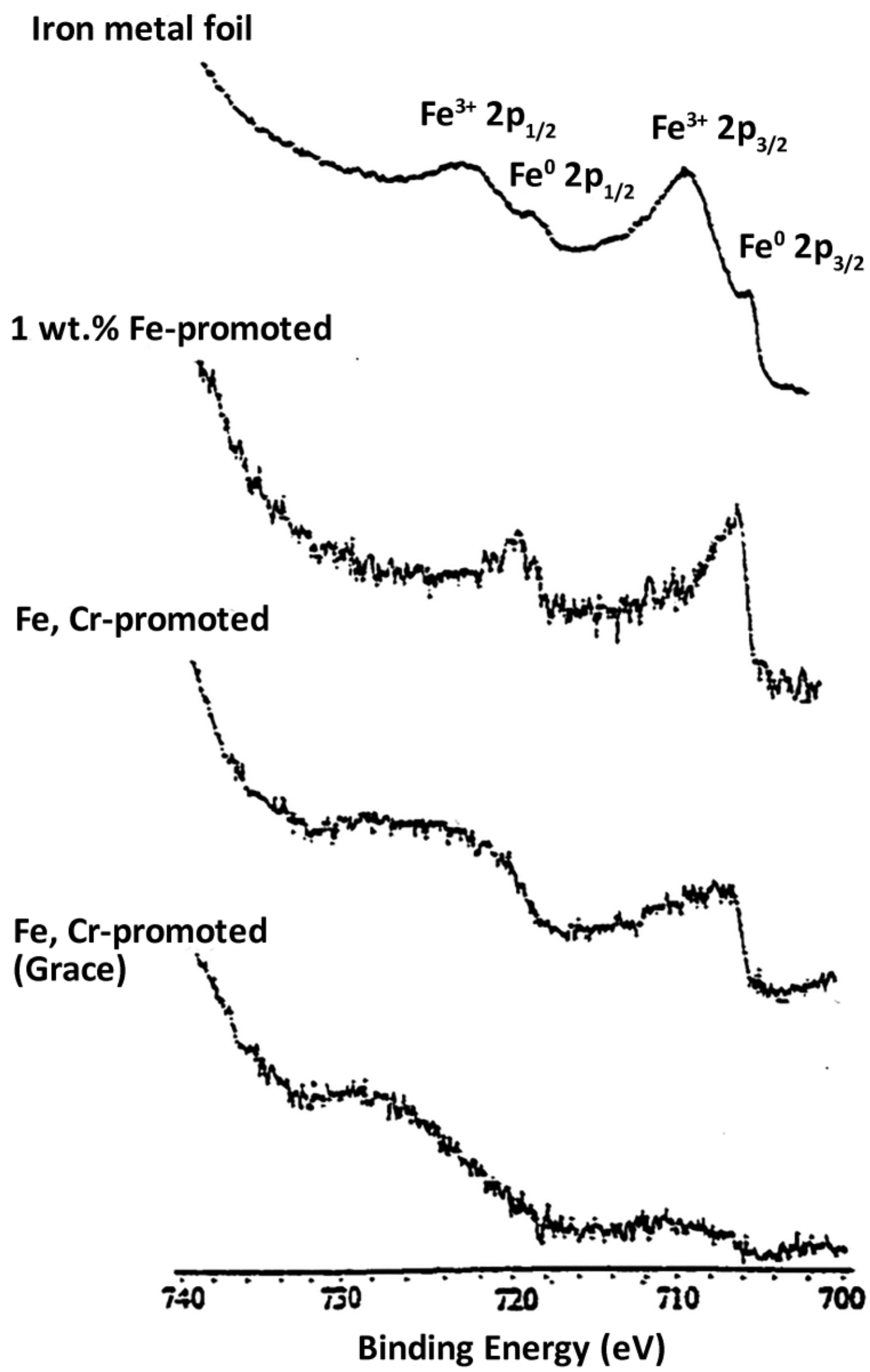

Figure S39: XPS spectra showing the chemical state of iron for the active catalysts.

Table S1: Surface and bulk composition element atomic ratios of nickel catalysts. The surface ratios were measured by x-ray photoelectron spectroscopy (XPS). The bulk ratios were measured by atomic absorption (AA) spectroscopy.

	**unpromoted catalyst**		**low Cr catalyst**		**high Cr catalyst**		**low Fe catalyst**		**doubly-promoted catalyst**	
**catalyst**	**XPS**	**AA**	**XPS**	**AA**	**XPS**	**AA**	**XPS**	**AA**	**XPS**	**AA**
**Al:Ni**	0.38	0.49	0.14	0.08	0.71	0.09	0.48	0.23	0.91	0.21
**Cr:Ni**	—	—	0.63	0.07	1.7	0.15	—	—	0.33	0.07
**Fe:Ni**	—	—	—	—	—	—	0.02	0.08	0.06	0.05

S7. Hydrogen temperature program desorption (TPD) curve

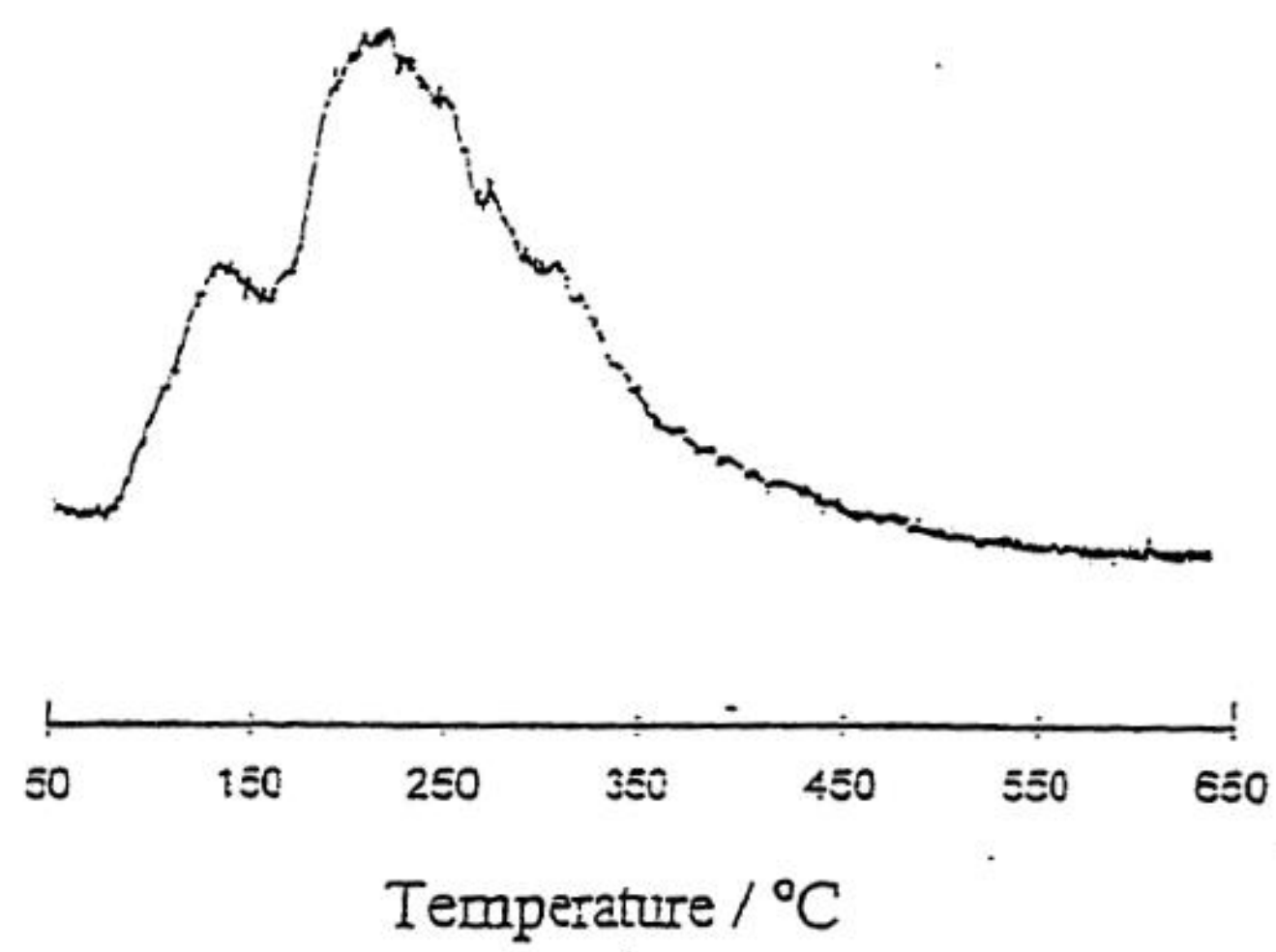

Figure S40: Temperature programmed desorption of hydrogen for unpromoted nickel catalyst.

S8. Safety considerations

The hot temperatures involved in alloy synthesis required appropriate safety measures. These safety measures included (but were not limited to) using tongs and thermal gloves when handling hot samples.

During leaching, the alloy powder was added slowly to avoid too much hydrogen gas being released at once. The leaching experiments were performed in a fume hood so the hydrogen gas produced was adequately diluted and ventilated. Precautions were taken to ensure there were no ignition sources near the hydrogen gas.

Hydrogen gas was never introduced into a closed vessel containing air. A closed vessel was always purged with an inert gas such as nitrogen before introducing hydrogen gas.

Nickel and some of its compounds are carcinogenic, so it was important to limit exposure to and inhalation of dusts, powders, and fumes containing nickel. Precautions were taken to avoid inhalation of all fine metal powders.

Safe handling of compressed gas cylinders was important. A gas cylinder was always secured and never moved unless properly capped. The cylinder valve was always closed when connecting and disconnecting gas lines. After connecting gas lines, the pipe connections were carefully checked for leaks and adjusted to fix them.

The activated catalysts were pyrophoric. To the extent feasible, direct contact with air was avoided by keeping the catalysts wet whenever they were handled under ambient conditions. The spent catalysts can be deactivated with dilute (e.g., 1 wt.% NaOCl) aqueous bleach solution before disposal.

This project involved working with metal powders, volatile solvents, strong acids, strong bases, and other toxic chemicals. The Safety Data Sheet for each chemical was consulted for specific hazards and precautions associated with each particular chemical. Appropriate safety measures including personal protective equipment such as gloves, googles, and lab coat were worn when handling all chemicals. Volatile and flammable solvents were always used in a fume hood

with adequate ventilation and stored in sealed containers when not in use. The spent chemicals were sunsetted by the environmental and safety department.

This project also involved the use of laboratory equipment containing vacuum pumps and vacuum chambers. Some of the equipment also involved working with high pressures. Appropriate precautions were taken to ensure that such equipment was used properly and that all pressure changes were implemented using the proper procedures. All laboratory equipment was operated in accordance with the manufacturer's instructions.

Reference

S1. E. P. Barrett, L. G. Joyner, and P. P. Halenda, The determination of pore volume and area distributions in porous substances .1. Computations from nitrogen isotherms, *J. Am. Chem. Soc.* 1951, **73**, 373-380, doi: 10.1021/ja01145a126.
